# *Bacteroides fragilis* capsular polysaccharide A ameliorates ulcerative colitis in rat by recovering intestinal barrier integrity and restoring gut microbiota

**DOI:** 10.3389/fphar.2024.1402465

**Published:** 2024-12-24

**Authors:** Yijia Zhong, Xiujuan Chang, Zihan Zhao, Lijun Zheng, Gaobo Kuang, Ping Li, Chenxuexuan Liu, Yuqin Fan, Zhixuan Liang, Ke Zhuang, Qiuling Xie, Yangyang Liu

**Affiliations:** ^1^ College of Life Science and Technology, Jinan University, Guangzhou, China; ^2^ Guangzhou ZhiYi Biotechnology Co. Ltd., Guangzhou, China

**Keywords:** *Bacteroides fragilis*, capsular polysaccharide A, ulcerative colitis, intestinal barrier function, intestinal microbiota

## Abstract

*Bacteroides fragilis* (*B. fragilis*) is a Gram-negative, obligate anaerobic, commensal bacterium residing in the human gut and holds therapeutic potential for ulcerative colitis (UC). Previous studies have indicated that capsular polysaccharide A (PSA) of *B. fragilis* is a crucial component for its effectiveness, possessing various biological activities such as anti-inflammatory, anti-tumor, and immune-modulating effects. We previously isolated and characterized the *B. fragilis* strain ZY-312 from the feces of a healthy breastfed infant, and extracted its PSA, named TP2. In this study, we explored the impact of TP2 on colonic inflammation and delved into its potential mechanisms. Initially, we used 2,4,6-trinitrobenzenesulfonic acid (TNBS) to induce colitis in rats and found that TP2 treatment significantly ameliorated TNBS-induced weight loss, increased clinical scores, extensive ulcers, and intestinal epithelial damage in UC rats. Further analysis revealed that TP2 effectively restored the intestinal barrier integrity in UC rats by regulating the expression of Muc-2, tight junction proteins (ZO-1, occludin, claudin-1, and claudin-2), as well as apoptosis-related proteins Bcl-2, BAX, and Cleaved-Caspase-3. Additionally, TP2 suppressed the expression of pro-inflammatory cytokines TNF-α, IL-1β, IL-6, and IL23, while promoting the secretion of anti-inflammatory cytokines IL-10 and IL-22, thereby inhibiting the occurrence of inflammation. TP2 also downregulated the phosphorylation levels of AKT and PI3K, effectively inhibiting the abnormal activation of the PI3K/AKT signaling pathway. More interestingly, 16S rRNA sequencing results showed that TP2 restored the ecological imbalance of the rat intestinal microbiota, with an increase in beneficial bacteria such as *Lactobacillus* and *Limosilactobacillus* observed in the treatment group. In conclusion, TP2 through the regulation of intestinal barrier-related cells and proteins, inhibition of apoptosis, modulation of inflammation-related cytokine levels, and control of abnormal activation of the PI3K/AKT signaling pathway, restores intestinal barrier integrity. Additionally, by reshaping the ecological imbalance of the gut microbiota, TP2 ultimately alleviates ulcerative colitis in rats.

## 1 Introduction

Ulcerative colitis (UC) is a type of inflammatory bowel disease (IBD), characterized by chronic and recurrent inflammation and ulceration of the colon and rectum walls, often associated with a certain proportion of colorectal cancer (Gros and Kaplan, 2023). The pathogenesis of UC is not fully elucidated, but increasing evidence suggests that the interaction between intestinal microbiota, mucosal barrier, and inflammatory responses plays a crucial role in the course of UC (Yan et al., 2019). Dysbiosis of the intestinal microbiota leads to rapid growth of harmful bacteria, invading the intestinal epithelial cells and causing damage to the intestinal mucosal barrier (Shen et al., 2018). This intestinal barrier damage results in the invasion of pathogens and antigens, triggering abnormal activation of the PI3K/AKT signaling pathway and inducing the upregulation of a series of pro-inflammatory factors such as TNF-α, IL-1β, IL-6, and IL-23 (Jiang et al., 2019; Peng et al., 2021; Zheng et al., 2022). The excessive expression of these pro-inflammatory cytokines promotes the infiltration of macrophages and neutrophils into the mucosa, exacerbating the inflammatory response and causing further damage to the intestinal mucosa (Neurath, 2014). Therefore, promoting intestinal barrier function, reshaping dysbiosis of the intestinal microbiota, and inhibiting the abnormal activation of the PI3K/AKT signaling pathway are important strategies for treating UC. Currently, the mainstream therapeutic drugs for UC include 5-aminosalicylic acid, glucocorticoids, immunosuppressants, and monoclonal antibodies. However, these drugs primarily offer symptomatic relief and fall short in providing long-term alleviation from intestinal inflammation in UC patients, often accompanied by various side effects. For instance, 5-aminosalicylic acid may lead to allergies, nausea, vomiting, *etc.* (Akobeng et al., 2016); glucocorticoids are prone to induce metabolic disorders and osteoporosis, weakening the immune system (Ford et al., 2011); antibody drugs often trigger immune responses in the body, leading to the production of neutralizing antibodies, rendering them ineffective (Baert et al., 2003). Therefore, there is an urgent need to develop new, safe, and effective drugs for the treatment of UC.

*Bacteroides fragilis* is a Gram-negative, rod-shaped, obligately anaerobic bacterium that acts as a commensal resident within the human colon, playing a role in maintaining the host’s health. Previously, we isolated a non-toxic strain of *Bacteroides fragilis*, *ZY-312*, from the feces of a healthy infant (Deng et al., 2016; Wang et al., 2017; Xu et al., 2018). This strain has shown therapeutic potential for various intestinal diseases, including antibiotic-associated diarrhea, *Clostridium difficile* infection, and ulcerative colitis (Li et al., 2017; Zhang et al., 2018; Deng et al., 2019; Zhou et al., 2022; Zhang et al., 2023). A substantial portion of the *B. fragilis* genome is dedicated to the synthesis of capsular polysaccharides, and the biological activity of *B. fragilis* is closely related to its highly complex and dynamic capsular structure (Comstock and Kasper, 2006; Eribo et al., 2022). The capsular polysaccharide of *B. fragilis* is the first recognized symbiotic factor that regulates the development of the host immune system, reversing functional defects in germ-free animals (Erturk-Hasdemir and Kasper, 2018). Studies by Mazmanian et al. demonstrated that capsular polysaccharides are crucial for *B. fragilis* to repair colitis. Mice colonized with wild-type *B. fragilis* or orally gavaged with PSA could avoid *Helicobacter* pylori-induced colitis, while mice colonized with a mutant strain that does not produce PSA could not escape the development of colitis (Mazmanian et al., 2008). Furthermore, PSA has been proven effective in treating various conditions such as hepatitis (Pagliuca et al., 2016), meningitis (Wang et al., 2014), melanoma (Vétizou et al., 2015), colorectal cancer (Sittipo et al., 2018), pneumonia (Johnson et al., 2018), and asthma (Johnson et al., 2015). In summary, PSA plays a vital role in maintaining host health and exhibits strong pharmaceutical potential.

We isolated and purified PSA from *ZY-312* with an average relative molecular mass of 70 kDa, and named it TP2. Our previous research preliminarily confirmed that TP2 has therapeutic efficacy for UC similar to PSA reported in other literature, and it was observed that TP2 exerts its anti-inflammatory effects *in vivo* in an undegraded form (Zheng et al., 2020). However, the specific mechanism of how TP2 alleviates UC *in vivo* in its undegraded form is still not fully understood. This study aims to further validate the anti-inflammatory effects of TP2 in TNBS-induced rats colitis model and explore its potential anti-inflammatory mechanisms. The research focuses on assessing the impact of TP2 on intestinal barrier function and the intestinal microbiota ecosystem. Experimental results indicate that TP2 significantly alleviates TNBS-induced colitis in rats. Its mechanism of action includes the restoration of intestinal barrier function, inhibition of cell apoptosis, modulation of the expression of inflammatory factors, reshaping the intestinal microbiota, and inhibition of abnormal activation of the PI3K/AKT signaling pathway. This study provides a potential new therapy for treating UC and lays the foundation for further research into the anti-inflammatory mechanisms of TP2.

## 2 Materials and methods

### 2.1 Chemicals and reagents

Capsular polysaccharide TP2 was provided by Guangzhou Zhiyi Biotechnology Co., Ltd. (Guangzhou, China), with a total polysaccharide content exceeding 98%, protein content below 1%, and nucleic acid content less than 0.5%. Following preparation, it is stored in a freezer at −20°C. 2, 4, 6-trinitro-Benzenesulfonicacid (TNBS) was purchased from Sigma-Aldrich (St. Louis, United States), and prednisolone was obtained from SinePharm (Shanghai, China). Antibodies for Claudin-1 and Muc-2 were purchased from Abcam Biotechnology (Cambridge, United Kingdom), Claudin-1, PI3K, AKT, and GAPDH from Proteintech (Chicago, IL, United States), p-PI3K(Tyr458) and p-AKT (Ser473) from CST (Danvers, MA, United States), ZO-1 and Claudin-2 from Affinity Biosciences (Jiangsu, China), and Cleaved-Caspase-3, Bcl-2, BAX from Biodragon (Beijing, China). TNF-α, INF-γ, IL6, IL10, IL-17A, IL22 enzyme-linked immunosorbent assay (ELISA) kits were purchased from MultiSciences (Lianke) Biotechnology (Hangzhou, China), and the IL23 kit was obtained from JianglaiBiology (Shanghai, China). RIPA Lysis Buffer and Enhanced BCA Protein Assay Kit were purchased from Beyotime (Shanghai, China). All other reagents used in the experiments were of analytical grade.

### 2.2 Animals

Male Wistar rats (120 g ± 10 g) were procured from Guangzhou Ruige Biological Technology Co. Ltd (Guangzhou, China) with license number SCXK (Yue) 2023–0,059. The rats were accommodated in the animal facility of Guangzhou Ruige Biological Technology Co. Ltd., at a temperature of 21°C–24°C with a 12 h light/dark cycle. The animals were housed in IVC cages, with five rats per cage, and provided with unrestricted access to water from water bottles, along with *ad libitum* access to food. All animals were adapted to the new surroundings for at least 5 days before use in experiments. The animal use protocol listed below has been reviewed and approved by the Institutional Animal Care and Use Committee (IACUC) of Ruige Biotechnology (Number: 20,230,701–001).

### 2.3 Establishment of the UC model and TP2 treatment

A total of 60 male Wistar rats, six-week-old, were used in this study and were randomly allocated into the following groups: normal control group (NC), model control group (MC), 9 mg/kg prednisolone group (PD), 1 mg/kg TP2 group (LTP2), 2 mg/kg TP2 group (MTP2), and 4 mg/kg TP2 group (HTP2), each comprising 10 rats. To establish a colitis rat model, TNBS powder dissolved in 30% ethanol was used at a concentration of 60 mg/mL. This concentration has been shown to effectively induce colitis in previous studies (Motavallian-Naeini et al., 2012). Rats were fasted for 40 h before the experiment, during which they received subcutaneous injections of 5% glucose saline (10 mL/kg, once a day). On the first day of the experiment, fasting rats were anesthetized with intraperitoneal injection of 1% pentobarbital sodium at a dose of 40 mg/kg. The model control group, the prednisolone group, and the TP2 administration group used a disposable rectal administration tube inserted into the left colic flexure (approximately 8 cm from the anus) to induce colitis with TNBS enema (0.5 mL/piece). The normal control group received 0.5 mL of normal saline. To prevent enema fluid reflux, the animals’ heads were lowered for 15 min after enema, and they were kept in the Trendelenburg position until they woke up. On the first day post-modeling, the TP2 administration group and the prednisolone group received their respective drugs, while the blank group and the model group were instilled with the same volume of purified water for 7 days. Daily records were maintained for animal weights, and fecal status was scored. On day 8, animals were euthanized by cervical dislocation, and the colon was swiftly removed and washed with 0.9% saline. Measurements included the ulceration area, weight, and colon length (from the ileocecal junction to the anal margin) to calculate the weight/length (W/L) ratio.

### 2.4 Evaluating disease activity index (DAI) and scoring for the colon

DAI score is the important indicator to assess the pathological degree of colitis. According to Ho Pan Sham et al.’ report (Sham et al., 2018), the rat DAI scores were evaluated though the following formula: DAI = (weight loss percentage score + stool score + rectal bleed score)/3 (Table 1).

**TABLE 1:** DAI score.

Score	Mucosal damage	Inflammation	Crypt loss	Pathological change range
0	None	None	None	None
1	Mucus layer	Mild	1/3	1 ∼ 25%
2	Submucosa	Moderate	2/3	26 ∼ 50%
3	Muscular and serosa	Severe	100% with intact epithelium	51 ∼ 75%
4	Muscular and serosa	Severe	100% with epithelium lose	76 ∼ 100%

### 2.5 HE and PAS staining and analysis

To evaluate histopathological changes in the colon, both hematoxylin and eosin (HE) staining and Periodic Acid-Schiff (PAS) staining were conducted. Rat small intestinal tissue was fixed in 4% paraformaldehyde for 24 h, embedded in paraffin, and cut into 4 μm-thick slices for HE staining. Colonic mucosal damage was observed under a light microscope, and histopathological evaluation was based on the severity of inflammation, crypt loss, and mucosal damage, as outlined in Table 2 (Rahmani et al., 2020). PAS staining was performed following the manufacturer’s instructions to assess the structure of colonic goblet cells.

**TABLE 2:** The standard for HS evaluation.

Score	Weight loss (%)	Stool consistency	Rectal bleed
0	< 1	Well-formed pellets	No blood
1	1 ∼ 5	Loose stools	Slight redness in stools
2	5 ∼ 10	Pasty and semi-formed stools	Clear redness in stools
3	10 ∼ 20	Diarrhea	Severe redness in stools
4	> 20	Liquid stools	Rectal prolapse

### 2.6 Western blot analyses

The colon tissues were cut into small pieces and lysed in ice-cold RIPA lysis buffer with a protease and phosphatase inhibitor cocktail. Subsequently, The tissue was lysed by an ultrasonic cell disruption apparatus. The protein concentration was determined using a BCA kit and then heated with loading buffer. Protein samples were separated by 10% gel electrophoresis and transferred to PVDF membranes. After blocking with 5% skim milk for 1 h at room temperature, the membranes were washed with Tris-Buffered Saline with Tween 20 (TBST) three times and then incubated with primary antibodies (1:1,000) overnight at 4°C refrigerator. After incubation with secondary antibodies (1:2000) for 1 h at room temperature, the membranes were washed with TBST three times, and the proteins were detected by ECL using ChemiDoc Imaging System (Biorad). GAPDH was used as an internal reference.

### 2.7 Measurement of cytokine level in the colon by ELISA

Cytokine concentrations in colon tissue were quantified using ELISA. Colon tissue was homogenized in a PBS solution, centrifuged at 3,500 rpm for 20 min at 4°C, and the supernatant was collected and stored at −80°C for further analysis. Levels of cytokines (IL-1β, IL-6, IL-10, IL-17A, IL-22, IL-23, TNF-α, IFN-γ) were determined using ELISA kits following the manufacturer’s protocol.

### 2.8 Stool DNA extraction and 16s RNA pyrosequencing

The Rat feces were collected, and total DNA was extracted using the QIAmp DNA microbiome kit (Qiagen). The 16S rRNA gene (V3-V4 region) was sequenced using the Illumina MiSeq system. The effective tags were clustered into operational taxonomic units (OTUs) with a similarity of ≥97% using Usearch software (version 11.0.667). Chimeric sequences and singleton OTUs (with only one read) were removed, after which the remaining sequences were sorted into each sample based on the OTUs. The tag sequence with the highest abundance was selected as a representative sequence within each cluster. Bacterial and fungal OTU representative sequences were classified taxonomically by blasting against the RDP Database and UNITE fungal ITS Database, respectively. All alpha diversity indices (including Chao1,Simpson, and Shannon indices) was calculated with Mothur software (version 1.30) and beta diversity were analyzed by R vegan package (version 2.5–6). Difference comparison was used to identify features with significantly different abundances between groups using LefSe (version 1.1.0). PICRUSt (v1.1.4) was utilized to predict functional profiles based on Kyoto Encyclopedia of Genes and Genomes (KEGG).

### 2.9 Statistical analysis

All data are presented as mean ± SEM. Statistical data analysis and graph creation were performed using GraphPad Prism10 software (Ver. 8.2.0). For each figure, one-way ANOVA was used to evaluate the statistical significance of differences among group means. If significant differences were detected by ANOVA, Tukey’s *post hoc* test was applied to perform pairwise comparisons between each group’s mean and the means of all other groups. Significance levels were defined as follows: *p*-value < 0.05 is considered as significant, *p* < 0.01 as very significant, *p* < 0.001 as highly significant, *p* < 0.0001 as extremely significant and *p* > 0.05 as not significant (NS), respectively.

## 3 Result

### 3.1 TP2 administration ameliorates the symptoms of TNBS-Induced colitis

**FIGURE 1:**
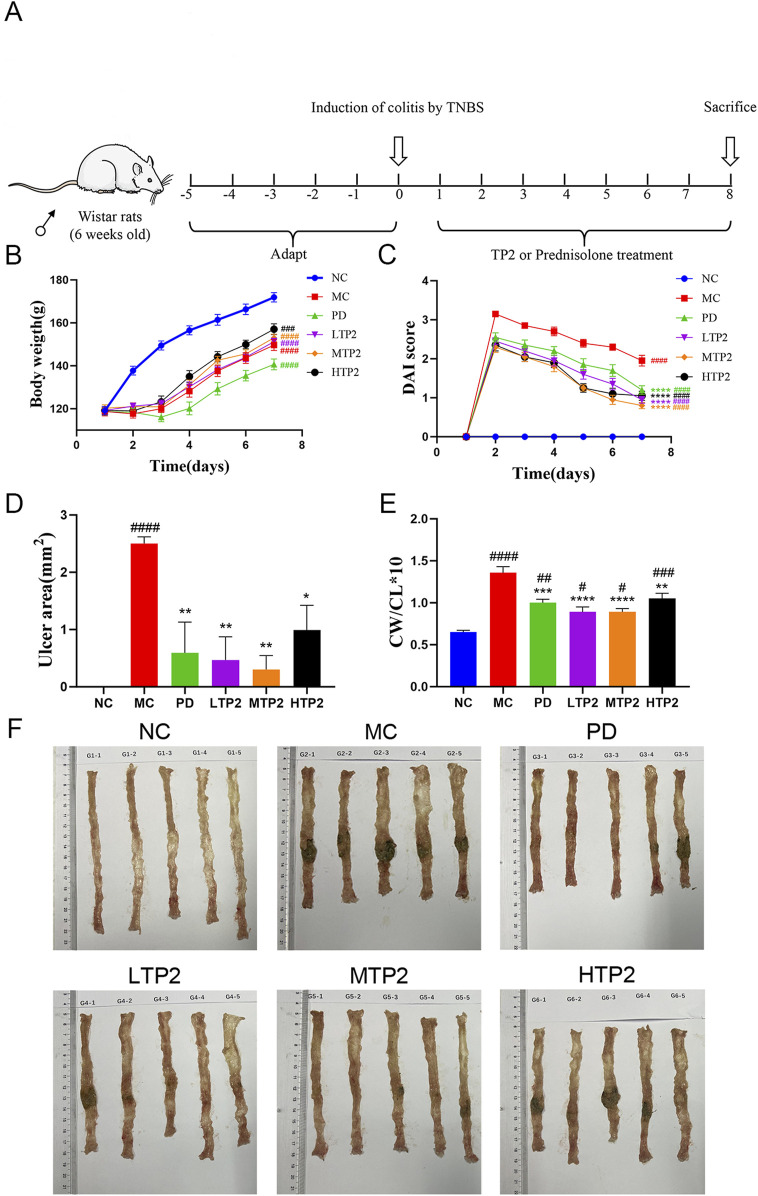
Experimental design and TP2 alleviate symptoms of Colitis. **(A)** The treatment regime of TP2 **(B)** Body weight **(C)** DAI score **(D)** Ulcer area **(E)** Colon weight/Colon length **(F)** Representative colon from each treatment group. Results reflected as mean ± SEM. ###*p* < 0.001, ####*p* < 0.0001 comparison to NC group. *p < 0.05, **p < 0.01, ***p < 0.001, ****p < 0.0001 comparison to MC group.

UC symptoms were assessed based on factors such as body weight, DAI, and colon-related characteristics. In comparison to the NC group, the final body weight of rats in all modeled groups significantly decreased, while the DAI scores in the MC group rats significantly increased. Rats in the LTP2, MTP2, HTP2 and PD groups exhibited a significant decrease in DAI scores compared to the MC group (*p* < 0.001) (Figures 1B, C). Additionally, we evaluated colon tissue ulcer area, colon length, and colon weight/colon length (CW/CL). The ulcer area in the MC group was significantly larger than in the NC group (*p* < 0.0001), whereas the LTP2, MTP2, HTP2 and PD groups showed a significant reduction in ulcer area compared to the MC group (*p* < 0.01, *p* < 0.01, *p* < 0.05 and *p* < 0.01, respectively) (Figures 1D, F). Colon length in the MC group was significantly shorter than in the NC group (Fig. F), while the LTP2 and HTP2 groups exhibited significantly longer colon lengths than the MC group. The CW/CL in the LTP2, MTP2, HTP2 and PD groups was significantly lower than in the MC group (*p* < 0.0001, *p* < 0.0001, *p* < 0.01 and *p* < 0.001, respectively) (Figure 1E). Thus, TP2 alleviates the symptoms of TNBS-induced colitis, indicating its potential as a therapeutic agent for UC.

### 3.2 TP2 alleviates histological changes of colon

**FIGURE 2:**
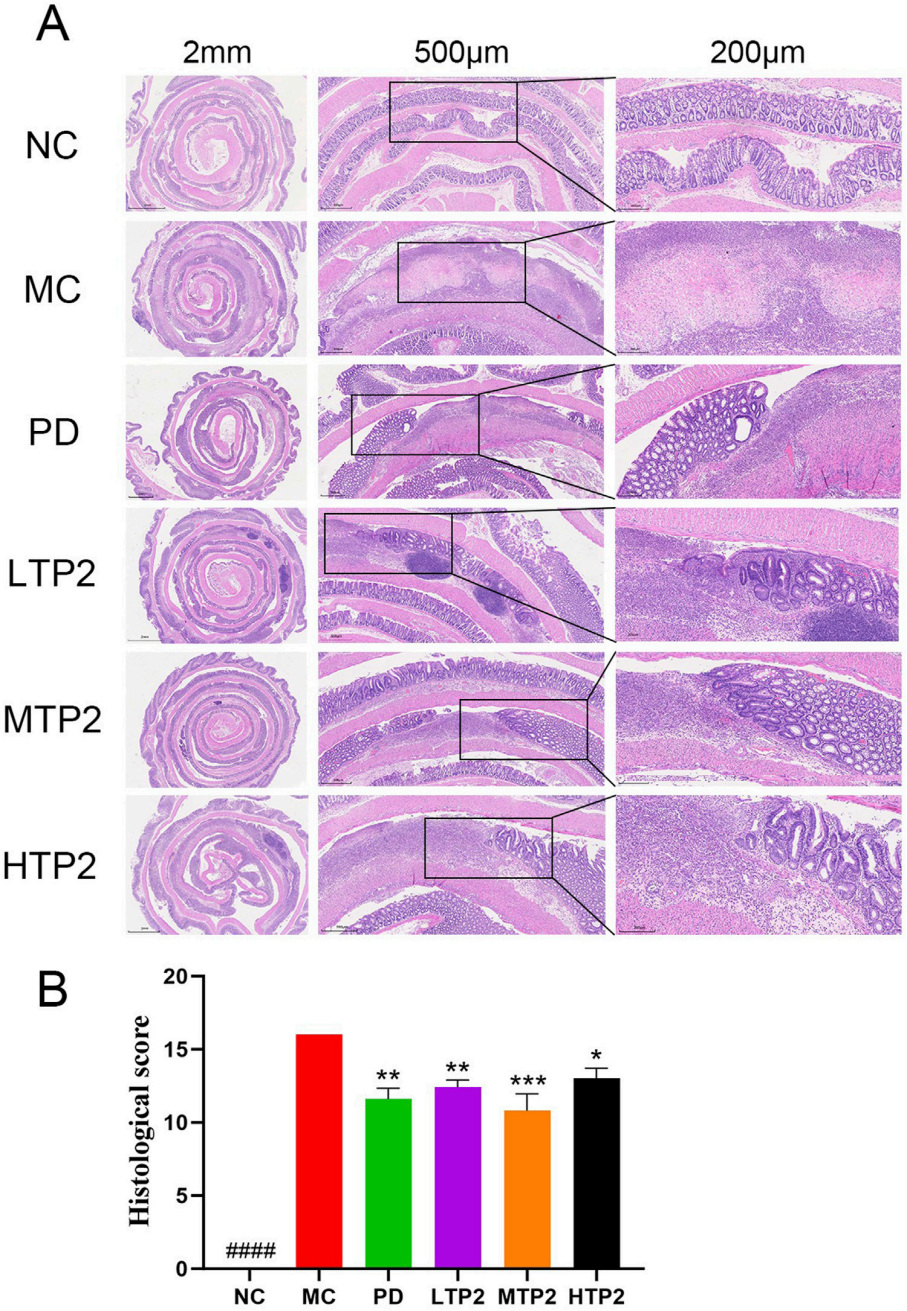
TP2 ameliorated colonic pathological damage in UC rats. **(A)** HE staining of colon tissue sections **(B)** Histopathological scores. ####*p* < 0.0001 comparison to NC group. *p < 0.05, **p < 0.01, ***p < 0.001, comparison to MC group. All data are presented as mean ± SEM (n = 5 rats per group).

We histologically assessed the function of TP2 inrestoring intestinal epithelial integrity by HE staining. In the NC group, the intestinal wall exhibited normal tissue architecture with intact mucosal structure, clear crypt architecture, and evenly distributed glands. The MC group showed the disappearance of normal colonic wall structure, accompanied by partial cell necrosis. Crypt cells, goblet cells, and the majority of glands were largely absent, with a noticeable infiltration of inflammatory cells. In comparison to the MC group, the LTP2, MTP2 and PD groups displayed a mostly intact colonic structure with fewer inflammatory lesions. There was a slight loss of crypt cells and goblet cells (Figure 2A). These results were corroborated by histological score analysis. The MC group had significantly higher scores than the NC group, indicating severe colonic damage induced by TNBS-induced ulcerative colitis. Both the LTP2, MTP2 HTP2 and PD groups had significantly lower scores than the MC group (*p* < 0.01, *p* < 0.001, *p* < 0.05 and *p* < 0.01, respectively), indicating TP2 significantly reduced histopathological damage (Figure 2B).

### 3.3 TP2 protectes the integrity of barrier damage in rat

**FIGURE 3:**
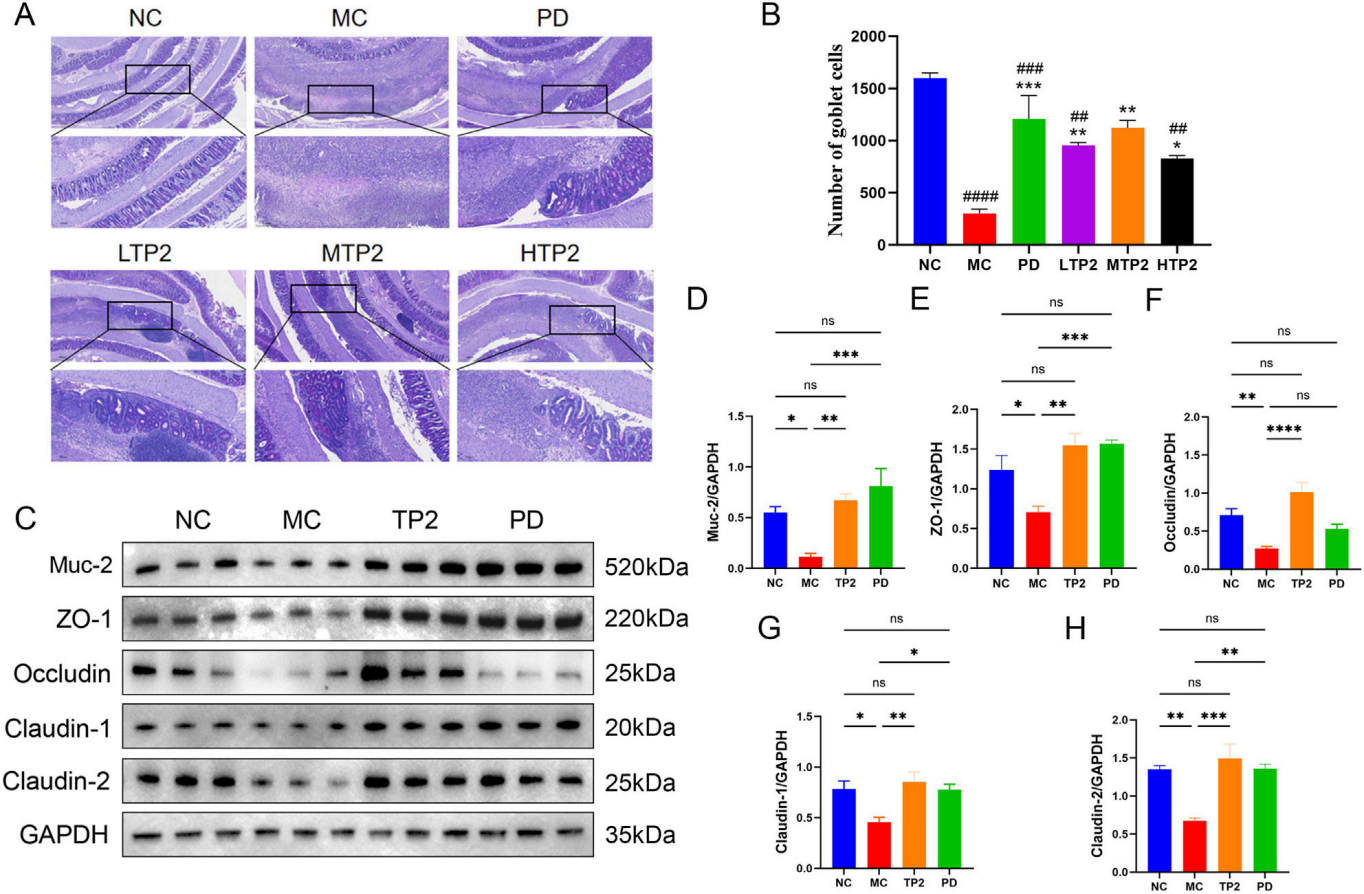
Therapeutic effects of TP2 on goblet cells and the intestinal barrier. **(A)** Representative PAS staining images **(B)** Quantification of goblet cells based on PAS staining. The relative protein expression of **(C)** Western blotting analysis of Muc-2, ZO-1, Occludin, Claudin-1 and claudin-2 in Colon tissue between groups **(D)** Muc-2 **(E)**ZO-1, **(F)** Occludin, **(G)** Claudin-1 and **(H)** Claudin-2 were normalized to GAPDH. ##*p* < 0.01, ###*p* < 0.001, ####*p* < 0.0001 comparison to NC group. *p < 0.05, **p < 0.01, ***p < 0.001 comparison to MC group. All data are presented as mean ± SEM (n = 5 rats per group).

The intestinal mucosal barrier serves as the host’s first line of defense, protecting the intestinal epithelium from invasion (Pelaseyed et al., 2014). Structural weakening of the colonic mucosal barrier promotes the development of ulcerative colitis (van der Post et al., 2019). We utilized PAS staining and Western blot analysis to evaluate the protective effects of TP2 on the intestinal mucosal barrier. The results indicate severe damage to goblet cells in the MC group, with a significant decrease in Muc-2 expression. Treatment with TP2 and PD significantly alleviated the TNBS-induced reduction in goblet cells and Muc-2 secretion (Figures 3A, B). Considering that the MTP2 group demonstrated optimal protective efficacy in clinical symptoms, HE staining, and PAS staining in colitis rats, we selected the MTP2 group for further analysis in subsequent mechanistic investigations (In the following discussions, TP2 will be used to represent this group).

Furthermore, intestinal tight junction proteins are crucial components of the intestinal barrier, preventing the leakage of harmful substances from the intestinal lumen (Buckley and Turner, 2018; Chelakkot et al., 2018). To investigate the protective effect of TP2 on TNBS-induced tight junction disruption, we measured the expression of ZO-1, Occludin, Claudin-1, and Claudin-2. The results showed a significant decrease in the expression of ZO-1, Occludin, Claudin-1, and Claudin-2 in the MC group compared to the NC group. TP2 and PD treatment, however, upregulated the expression levels of these proteins (Figures 3C–H). These findings suggest that TP2 restores the damaged intestinal barrier function by protecting goblet cells, upregulating Muc-2 expression, and enhancing the expression of tight junction proteins.

### 3.4 TP2 suppressed apoptosis in colon tissues

**FIGURE 4:**
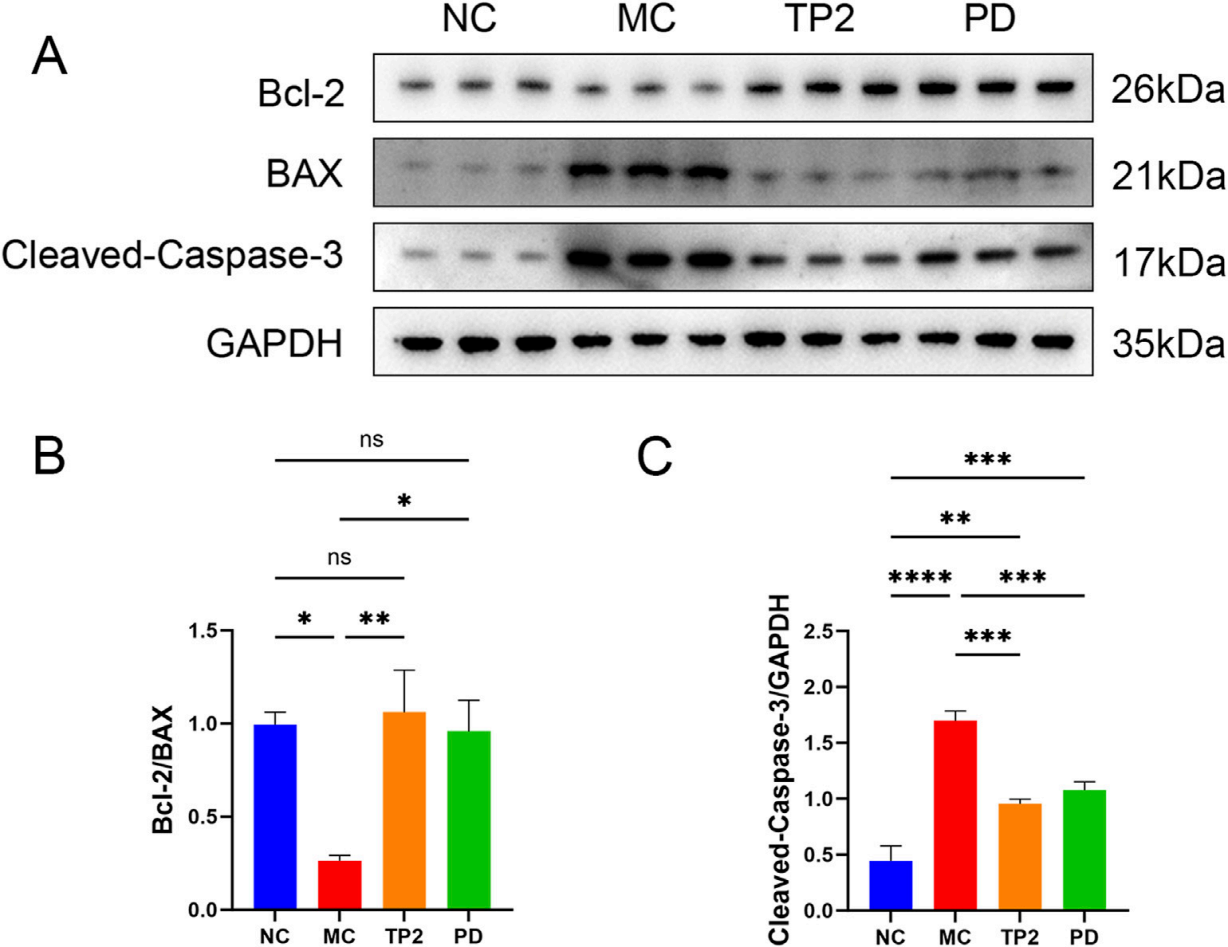
TP2 suppressed apoptosis in colon tissues **(A)** Western blotting analysis of Bcl-2, BAX and Cleaved-Caspase-3 in Colonic tissues **(B)** The relative protein expression levels of Bcl-2/BAX **(C)** The relative protein expression levels of Cleaved-Caspase-3/GAPDH. *p < 0.05, **p < 0.01, ***p < 0.001, ****p < 0.0001 and ns *p* > 0.05. All data are presented as mean ± SEM (n = 5 rats per group).

To assess apoptosis in colonic tissues, we investigated several proteins associated with apoptosis. Firstly, the proto-oncoproteins Bcl-2 and Bax were detected. As shown in Figure 4A, the expression of the anti-apoptotic protein Bcl-2 was downregulated, while the pro-apoptotic protein Bax was upregulated, resulting in a marked reduction in the Bcl-2/Bax ratio in the colonic tissues of TNBS-induced UC rats (*p* < 0.05). Additionally, Cleaved-Caspase-3 was selected to evaluate the effect of TP2 on TNBS-induced apoptosis. Under the challenge of TNBS, the expression of Cleaved-Caspase-3 significantly increased (*p* < 0.0001). By contrast, TP2 and PD treatment remarkably abrogated the downregulation of the Bcl-2/Bax ratio (*p* < 0.01, *p* < 0.05) (Figure 4B) and reduced Cleaved-Caspase-3 expression (*p* < 0.001, *p* < 0.001) (Figure 4C). In summary, TP2 suppressed apoptosis in colon tissues in TNBS-induced UC rats.

### 3.5 TP2 regulated cytokines and inflammatory factors in colon tissue

**FIGURE 5:**
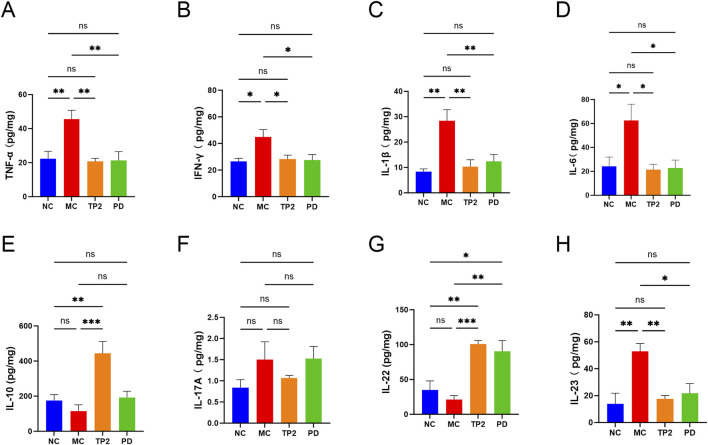
Effects of TP2 on inflammatory cytokines in colonic tissues. **(A)** TNF-α, **(B)** INF-γ, **(C)**IL-1β, **(D)** IL-6, **(E)** IL-10, **(F)** IL-17A, **(G)** IL-22 and **(H)** IL-23. *p < 0.05, **p < 0.01, ***p < 0.001 and ns *p* > 0.05. All data are presented as mean ± SEM (n = 5 rats per group).

To gain a deeper understanding of the mechanism by which TP2 alleviates colitis, we utilized ELISA technology to assess typical pro-inflammatory and anti-inflammatory cytokines in rats’ colonic tissues, including TNF-α, IFN-γ, IL-1β, IL-6, IL-10, IL-17A, IL-22, and IL-23. As depicted in Figure 5, the MC group showed significantly increased levels of TNF-α (*p* < 0.05), IFN-γ (*p* < 0.05), IL-1β (*p* < 0.01), IL-6 (*p* < 0.01), and IL-23 (*p* < 0.01), indicating serious inflammation in UC rats. In comparison to the MC group, TP2 significantly enhanced the expression of IL-10 (*p* < 0.0001)and IL-22 (*p* < 0.05) in the colonic tissues of colitis-induced rats. Simultaneously, it reduced the levels of TNF-α (*p* < 0.055), IL-1β (*p* < 0.05), IL-6 (*p* < 0.05) and IL-23 (*p* < 0.01), while TP2 did not exhibit a significant impact on IFN-γ and IL-17 A. These research findings suggest that TP2 can promote the expression of anti-inflammatory cytokines and inhibit the secretion of pro-inflammatory cytokines, thereby mitigating colitis.

### 3.6 TP2 inhibited PI3K/AKT signaling pathway in colonic tissues

**FIGURE 6:**
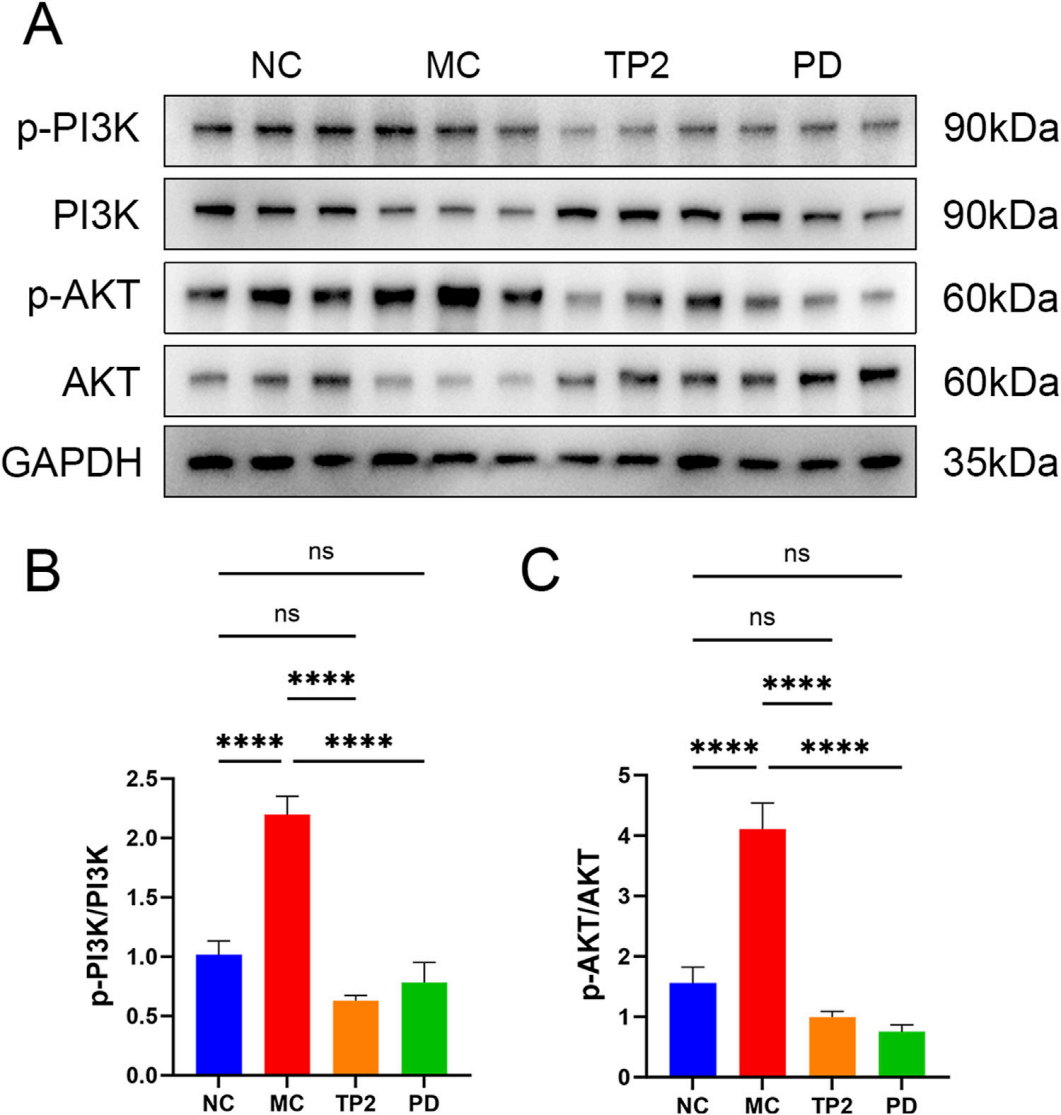
TP2 ameliorates TNBS-induced colitis relying on PI3K/AKT signaling pathway. **(A)** Protein levels of phosphor-PI3K, total PI3K, phosphor-AKT and total PI3K in colonic tissues were assessed by Western blotting **(B)** Representative protein levels of p-PI3K/PI3K **(C)** Representative protein levels of p-AKT/AKT. ****p < 0.0001 and ns *p* > 0.05. All data are presented as mean ± SEM (n = 5 rats per group).

Immunological dysregulation stands as a pivotal pathogenic factor in UC, manifested through an aberrant balance in the expression of pro-inflammatory and anti-inflammatory cytokines. The classical PI3K/AKT signaling pathway plays a crucial role in modulating diverse cellular processes, encompassing proliferation, differentiation, apoptosis, and inflammation-related responses. Numerous investigations underscore the interconnectedness of cytokine expression and closely associated proteins with the PI3K/AKT signaling pathway in the progression of UC (Huang et al., 2011; Chen et al., 2017; Li et al., 2022a). Consequently, we conducted Western blot analysis to scrutinize the influence of TP2 on the PI3K/AKT signaling pathway. The data revealed no discernible disparities in the total expression levels of PI3K and AKT across the experimental groups. Nevertheless, a noteworthy elevation in the expression levels of p-PI3K and p-AKT was observed in the MC group, signifying the induction of PI3K/AKT signaling pathway activation by TNBS (Figure 6A). Remarkably, treatment with TP2 and prednisolone effectively reversed this observed trend (Figures 6B, C). This aligns with the established regulatory effects of TP2 on inflammation, tight junction proteins, and cellular apoptosis. In summary, our research findings substantiate the proposition that TP2 mitigates TNBS-induced colonic injury by impeding the activation of the PI3K/AKT pathway.

### 3.7 TP2 reinstate gut microbiota dysbiosis

**FIGURE 7:**
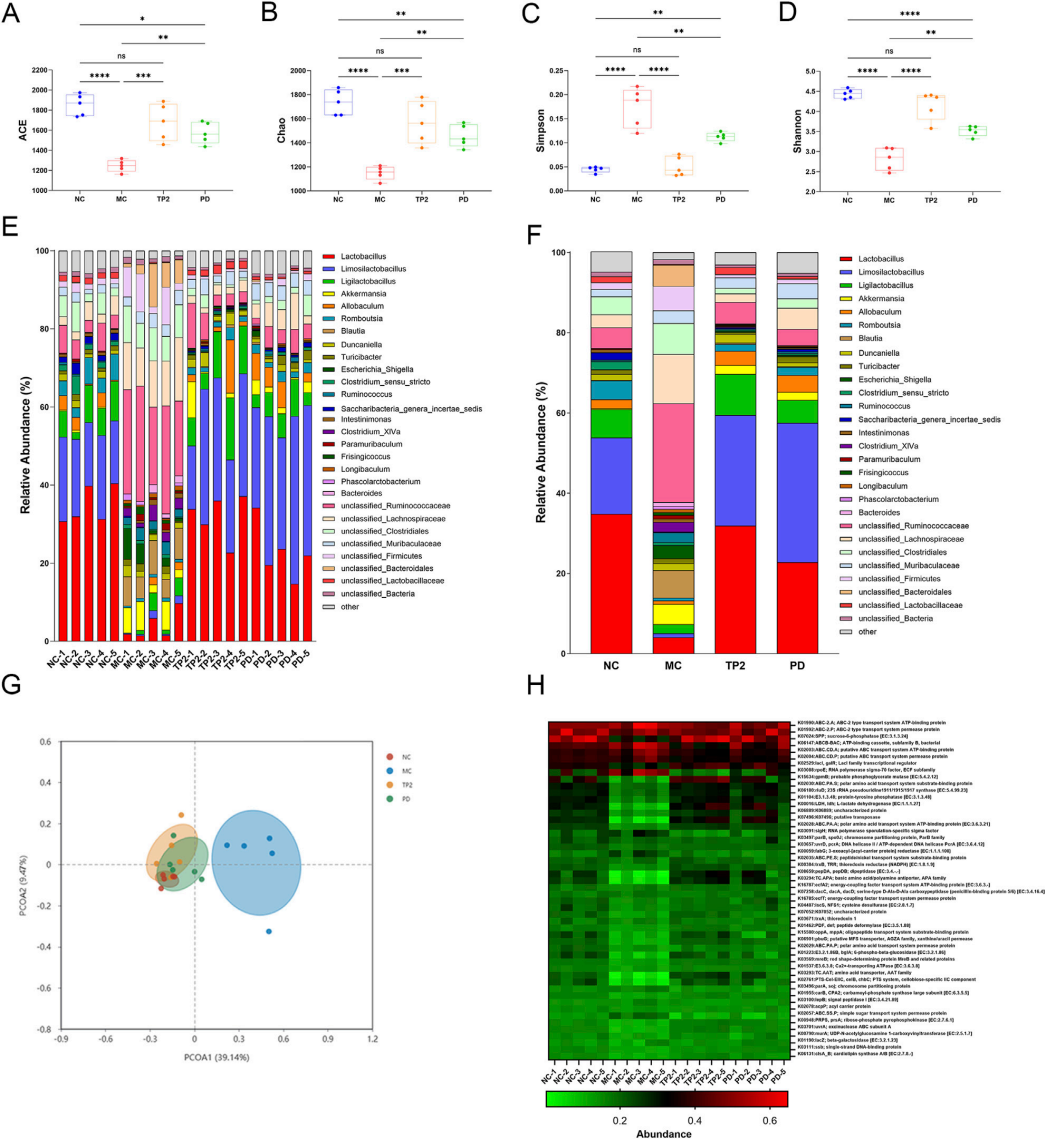
Effects of TP2 on intestinal microbiota in TNBS-induced rtas. **(A)** ACE **(B)** Chao **(C)** Simpsom **(D)** Shannon **(E)** Relative abundances of microbial genera in each sample **(F)** Relative abundances of microbial genera in each group **(G)** PCoA analysis **(H)** Relative abundances of microbial genera in each group **(H)** Microbial community functions against KEGG database between each group predicted by PICRUSt. *p < 0.05, **p < 0.01, ***p < 0.001, ****p < 0.0001 and ns *p* > 0.05. Values are mean ± SEM (n = 5 rats per group).

To investigate the changes in the structure and function of rat intestinal flora after drug administration, we performed 16S rRNA gene amplicon sequencing. Alpha diversity analysis at the OTU level showed that multiple indices (ACE, Chao, Shannon, Simpson) differed significantly in different groups (Figures 7A–E), the results of Principal Co-ordinates Analysis (PCoA) at the OTU level showed the same trend (Figure 7G), in which the MC group showed a significant separation compared to the NC group, and the TP2 group showed clustering with the PD and NC groups. The mean relative abundance at the genus level showed that TNBS reduced the abundance of major genera such as *Lactobacillus, Limosilactobacillus, Ligilactobacillus* and *Akkermansia* compared to the NC group, with the opposite trend in the TP2 and PD groups relative to the MC group (Figures 7E, F). These results suggest that TP2 treatment restored TNBS-induced disruption of UC intestinal microbiota. In addition, Functional prediction analysis of KEGG for bacteria and archaea was performed using PICRUSt (Figure 7G), Functional abundance heatmap showed that the abundance of probable phosphoglycerate mutase [EC:5.4.2.12], protein-tyrosine phosphatase [EC:3.1.3.48], probable phosphoglycerate mutase [EC:5.4.2.12] and L-lactate dehydrogenase [EC:1.1.1.27] was different in MC group and NC group, however, the abundance was similar in TP2 group and PD group and NC group. This may predict an important role for these KEGG ontologys (KOs) in the pathogenesis of inflammatory bowel disease.

## 4 Discussion

The TNBS-induced model is widely recognized for its efficacy in replicating the pathological features of UC (Morris et al., 1989; Zhu et al., 2019; Bilsborough et al., 2021). Extensive research has consistently shown that TNBS can reliably induce a UC-like condition, characterized by inflammation predominantly affecting the mucosal and submucosal layers. This includes the formation of ulcers, crypt abscesses, goblet cell depletion, and significant inflammatory cell infiltration closely resembling the clinical and histopathological presentation of human UC (Silva et al., 2019). In this experiment, UC was induced in rats by administering a TNBS/ethanol mixture solution through the rectum. On the 2nd day of modeling, rats in the model group exhibited typical physiological symptoms of colitis, including weight loss, diarrhea, sticky stools with blood, and even anal bleeding. On the 8th day during dissection, the colon tissues of rats in the model group were significantly shortened, with large areas of ulcers observed. HE staining revealed the disappearance of colonic crypt structures, severe inflammation, and partial tissue necrosis. PAS staining and Western blotting demonstrated a significant decrease in the number of colonic goblet cells and mucin expression. These findings confirm the successful establishment of the UC rat model.

**FIGURE 8:**
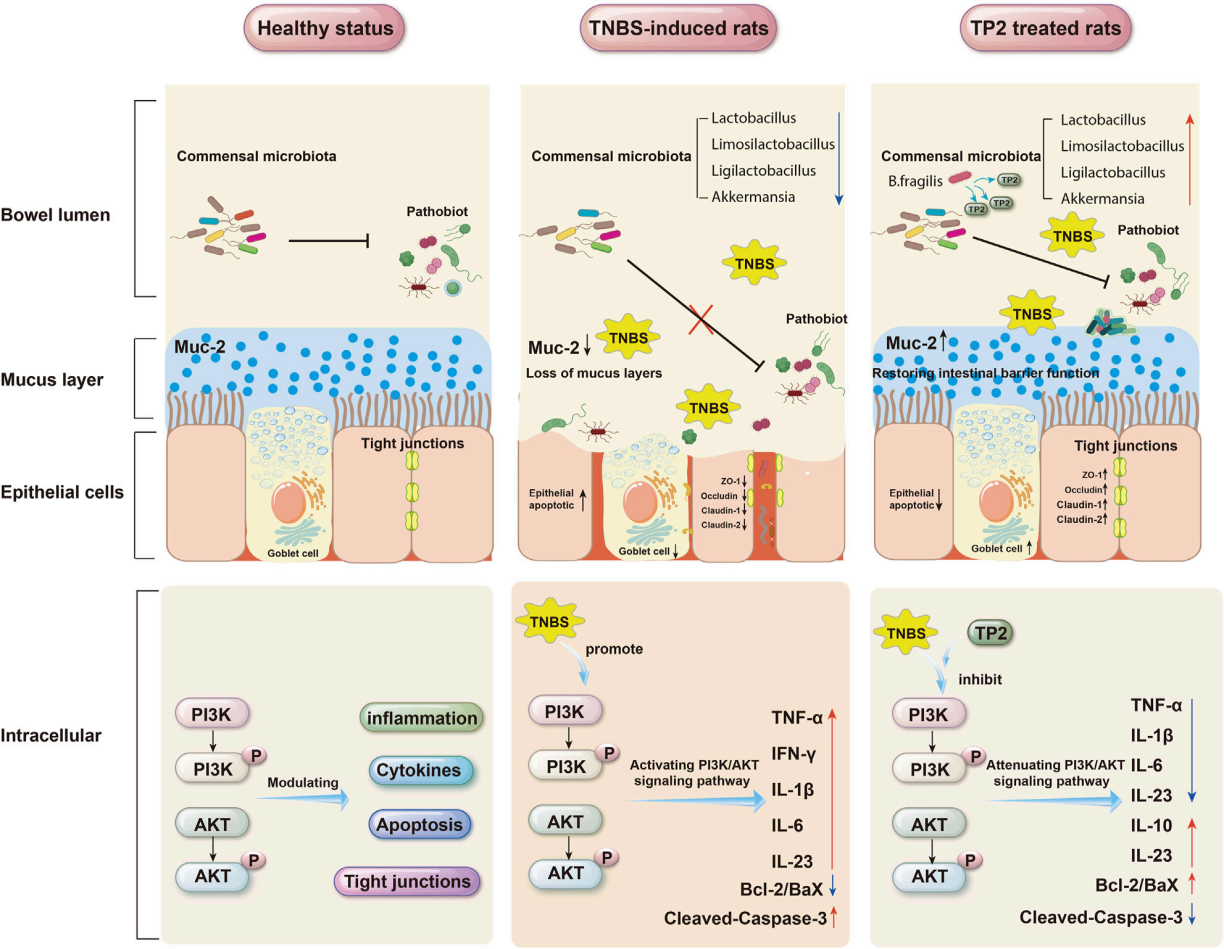
Mechanism of TP2 mitigating TNBS-induced UC.

TP2, derived from the *B. fragilis* strain *ZY-312*, is a capsular polysaccharide. Previous studies have provided preliminary evidence of TP2’s efficacy in alleviating UC. However, the precise mechanism by which TP2 exerts its therapeutic effects in colitis remains unclear. This study aimed to further validate the therapeutic potential of TP2 in a rat model of TNBS-induced ulcerative colitis and explore the underlying mechanisms, including its impact on intestinal barrier function, microbial composition, and inflammation. The experimental results confirmed a significant alleviation of TNBS-induced colitis in rats following TP2 treatment, indicating a robust therapeutic effect. Figure 8 illustrates the comprehensive mechanisms by which TP2 mitigates TNBS-induced ulcerative colitis, demonstrating its effects across various systems including the gut microbiota, mucus layer, epithelial cells, and intracellular signaling pathways. TP2 intervention effectively ameliorated clinical manifestations such as weight loss, increased DAI score, large-area ulcers, and damage to the intestinal epithelium in UC rats. Further analyses demonstrated that TP2 contributed to the restoration of the rat intestinal barrier’s integrity, suppression of intestinal inflammatory responses, and inhibition of apoptosis in intestinal epithelial cells. Notably, TP2 played a crucial role in reshaping the gut microbiota. Additionally, TP2 significantly downregulated the expression of the PI3K/AKT signaling pathway, showcasing excellent anti-inflammatory properties.

Intestinal epithelial cells, along with the mucous layer, form the physiological barrier of the intestine—a critical defense against the invasion of toxic substances (Kotla et al., 2022). The intestinal mucous layer isolates bacteria from epithelial cells, safeguarding them against bacterial invasion and exposure to toxic substances (Hansson, 2012). Goblet cells and intestinal tight junction proteins are essential components of the intestinal barrier. Goblet cells, as specialized epithelial cells, are responsible for producing and secreting mucus, forming the epithelial scaffold of the mucous layer (Gonzalez-Perez et al., 2021; Kang et al., 2022). Tight junction proteins serve as the tightest adhesion between epithelial cells and endothelial cells, selectively allowing ions and small molecules from the intercellular space to pass through (Günzel and Fromm, 2012). Key tight junction proteins, including ZO-1, Occludin, Claudin-1, and Claudin-2, play a role in influencing the development of intestinal inflammation through the regulation of mucosal homeostasis and intestinal permeability (Ulluwishewa et al., 2011; Buckley and Turner, 2018; Chelakkot et al., 2018). Our research findings indicate that rats displayed typical colitis symptoms after TNBS induction. The MC group exhibited a significant loss of goblet cells and reduced expression of tight junction proteins, suggesting that TNBS induction impaired the colonic barrier function in rats. When the colonic barrier function is compromised, intestinal permeability increases, enabling harmful bacteria and excessive toxic substances to enter the colon, leading to inflammation. Following TP2 treatment, there was a significant increase in the number of goblet cells in colonic tissues. Moreover, the expression levels of Muc-2 and tight junction proteins ZO-1, Occludin, Claudin-1, and Claudin-2 were markedly upregulated. These findings suggest that TP2 protects the integrity of the intestinal barrier by augmenting the number of goblet cells and enhancing the expression of tight junction proteins. This action inhibits the entry of pathogenic factors into colonic tissues, thereby alleviating inflammation.

Research also indicates a close association between the disruption of tight junctions in intestinal tissues and inflammatory responses (Luissint et al., 2016). Under inflammatory conditions, cytokine-mediated dysfunction of tight junction barrier function promotes the development of intestinal diseases (Lee, 2015). Elevated levels of pro-inflammatory cytokines, such as TNF-α, IL-1β, IL-6, and IL-23 in colonic tissues, further exacerbate colonic damage and recruit a large number of inflammatory cells into the inflamed tissue (McGuckin et al., 2009). These cytokines are also associated with complications of colitis, including cell apoptosis, rectal bleeding, and the formation of colitis-associated cancer (Neurath, 2014). We observed a significant increase in the expression of pro-inflammatory cytokines such as TNF-α, IL-1β, IL-6, and IL-23 in the MC group compared to the NC group, while TP2 treatment inhibited the expression of these cytokines. In contrast to the aforementioned cytokines, anti-inflammatory cytokines such as IL-10 and IL-22 can restore intestinal barrier defects by enhancing intestinal cell permeability and promoting the expression of tight junction protein Claudin-2, respectively (Sun et al., 2008; Keir et al., 2020). In this study, we found that TP2 significantly promoted the secretion of anti-inflammatory cytokines IL-10 and IL-22. These research results suggest a close correlation between TP2 alleviating colonic damage and suppressing inflammation.

Besides the impact of inflammatory factors on the integrity of intestinal tissues, abnormal apoptosis of intestinal epithelial cells can also disrupt the integrity and barrier function of the intestinal mucosa (Blander, 2016; Subramanian et al., 2020). Previous studies found a significant increase in the apoptosis rate of intestinal epithelial cells in UC patients (Garcia-Carbonell et al., 2019). Inhibiting apoptosis of intestinal epithelial cells is crucial for improving the impaired intestinal barrier function in UC patients. Bcl-2 and Bax, key members of the mitochondrial apoptosis pathway, determine the balance between cell proliferation and apoptosis (Adams and Cory, 1998). Also, Caspase-3 and its cleavage product Cleaved-Caspase-3 play a crucial regulatory role in cell apoptosis (Riedl and Shi, 2004). The PI3K/AKT pathway, an important signaling pathway associated with apoptosis, regulates extracellular signals, influencing downstream effector molecules such as Bcl-2, Bax, and Caspase-3, thereby impacting inflammation, cell apoptosis, and the proliferation and differentiation of immune cells (Cantley, 2002; Chen et al., 2017; Jalil et al., 2023). Abnormal activation of the PI3K/AKT pathway has been found to lead to immune system imbalance and is one of the pathogenic mechanisms of UC (Nunes-Santos et al., 2019). Inhibiting the PI3K/AKT signaling pathway has significant benefits for alleviating UC (Park et al., 2014; Li et al., 2022a; Zhou et al., 2023) and is considered a promising target for treating UC in humans (Tokuhira et al., 2015). In this study, we observed that TP2 inhibited the activation of the PI3K/AKT signaling pathway by reducing the expression levels of p-PI3K and p-AKT.

In recent years, increasing evidence suggests that the dysregulation of the gut microbiota plays a crucial role in the pathological process of colitis (Lane et al., 2017). The interaction between the gut microbial community and the host is essential for maintaining the normal function of the intestinal epithelial barrier, regulating host immune responses, and ensuring the physiological health of the host (Heiman and Greenway, 2016). Disruption of the gut microbial community can trigger host immune reactions, activate inflammatory signaling pathways, and consequently lead to the occurrence of colitis (Park et al., 2017). Differences in the gut microbiota between UC patients and healthy individuals mainly include reduced microbial diversity and diminished abundance of specific bacteria (Miyoshi and Chang, 2017). 16S rRNA sequencing results indicate that TNBS induction results in a significant decrease in the abundance and diversity of the gut microbial community compared to normal rats, and TP2 treatment significantly improves this condition. PCoA analysis shows a distinct separation between the MC group and TP2 group rats, and the TP2 group tends to cluster with the NC group, indicating that TP2 treatment modifies the TNBS-induced changes in the gut microbiota. At the genus level, TNBS induction decreases the abundance of *Lactobacillus*, *Limosilactobacillus*, *Ligilactobacillus*, and *Akkermansia*, while increasing the abundance of *unclassified_Ruminococcaceae* and *unclassified_Lachnospiraceae.* Prior research has indicated that *Lactobacillus* and *Limosilactobacillus* can enhance the intestinal mucosal barrier by promoting the expression of intestinal tight junction proteins through interaction with host intestinal epithelial cells. Additionally, they regulate the host immune system by modulating inflammatory factors and immune cells (He et al., 2022; Luo et al., 2023). *Ligilactobacillus* has been found to contribute to nutrient absorption and the production of short-chain fatty acids (Li et al., 2022b). *Akkermansia* has been found to be negatively correlated with several pro-inflammatory cytokine levels and positively correlated with the intestinal tight junction protein ZO-1 (Ye et al., 2018). These results suggest that TP2 may alleviate colitis in rats by reshaping the structure and composition of the intestinal microbiota, influencing the physiological functions of the host.

## 5 Conclusion

This study confirmed the therapeutic effect of the capsular polysaccharide A of *Bacteroides Fragilis* TP2 on TNBS-induced UC in Wistar rat models. Furthermore, it revealed that TP2 restores intestinal barrier integrity by regulating barrier-related cells and proteins, inhibiting cell apoptosis, modulating levels of inflammation-related cytokines, and controlling the abnormal activation of the PI3K/AKT signaling pathway. Additionally, TP2 alleviates rat UC by reshaping the ecological imbalance of the intestinal microbiota.

## Data Availability

The processed 16S rRNA data has is deposited in the NCBI database, BioProject accession number PRJNA1189676; available at: https://www.ncbi.nlm.nih.gov/bioproject/PRJNA1189676. Further inquiries can be directed to the corresponding authors.
