# Supplementary figures and images for: Bacteroides fragilis capsular polysaccharide A ameliorates ulcerative colitis in rat by recovering intestinal barrier integrity and restoring gut microbiota
Source: Front Pharmacol. 2024 Dec 24;15:1402465. doi: 10.3389/fphar.2024.1402465 (PMC11703662; doi:10.3389/fphar.2024.1402465)

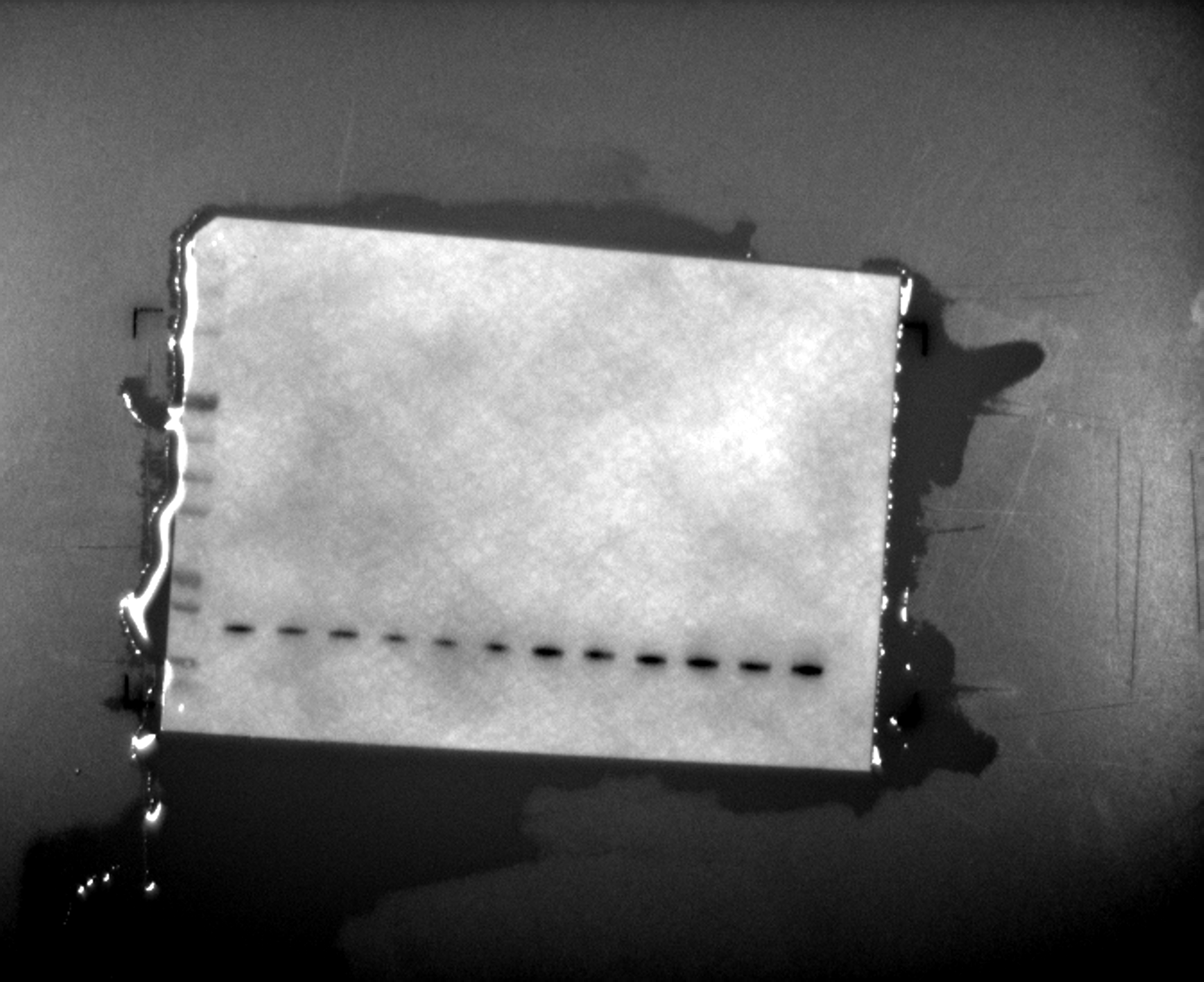

Supplement: Supplementary file 1 [file DataSheet3.ZIP › Figure 3/Claudin-1_Gel-1.Tif]

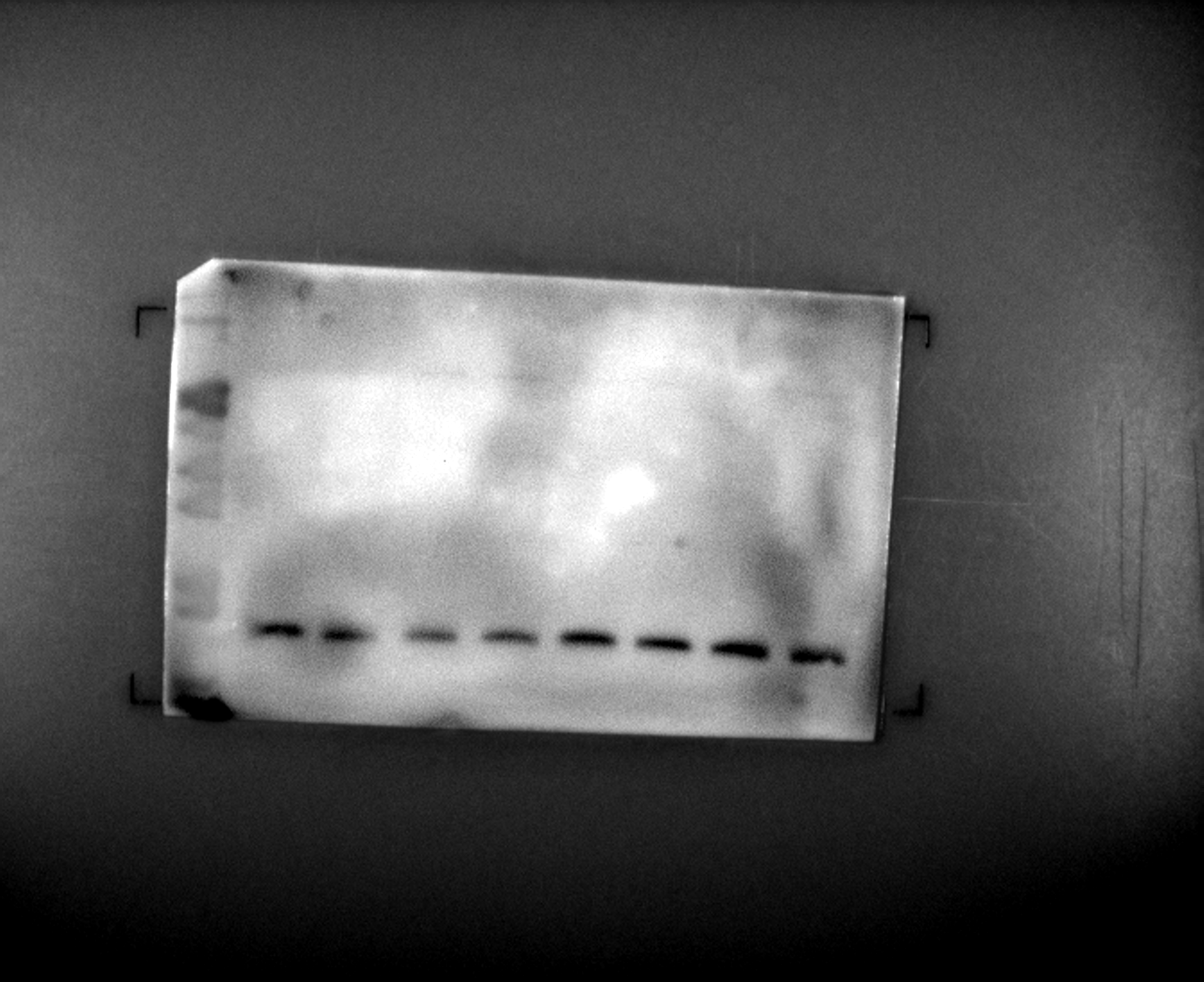

Supplement: Supplementary file 1 [file DataSheet3.ZIP › Figure 3/Claudin-1_Gel-2.Tif]

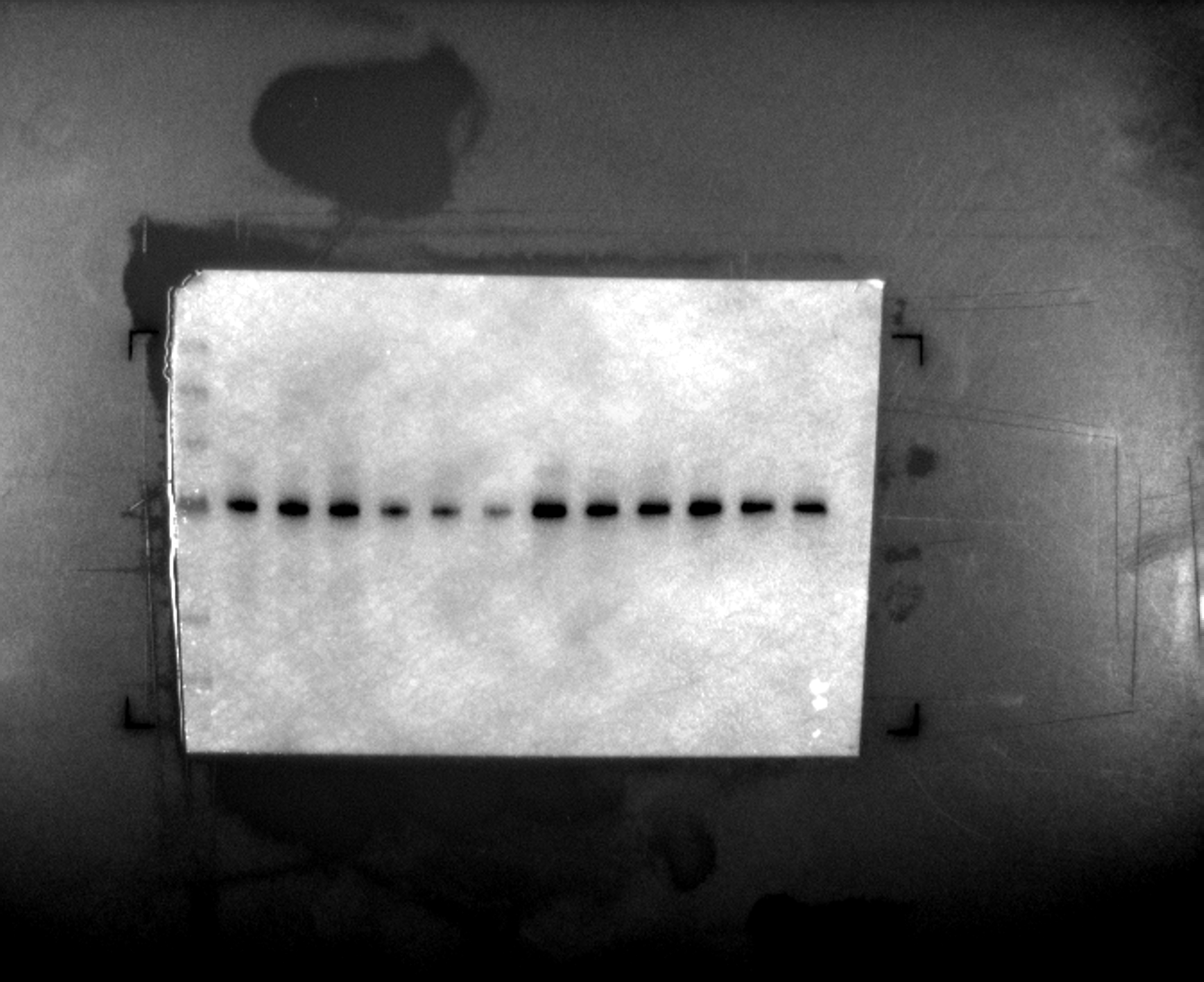

Supplement: Supplementary file 1 [file DataSheet3.ZIP › Figure 3/Claudin-2_Gel-1.Tif]

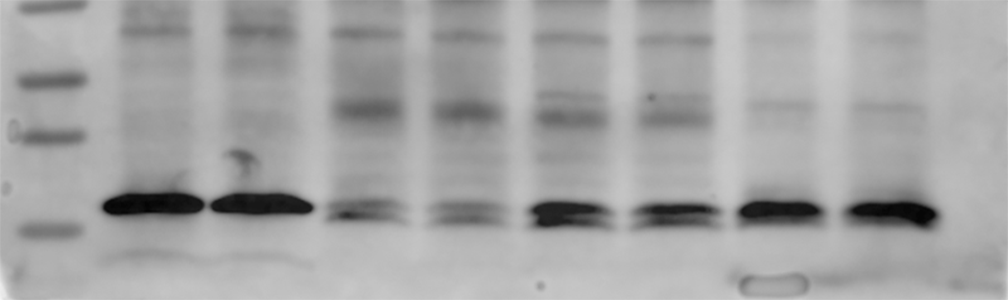

Supplement: Supplementary file 1 [file DataSheet3.ZIP › Figure 3/Claudin-2_Gel-2.tif]

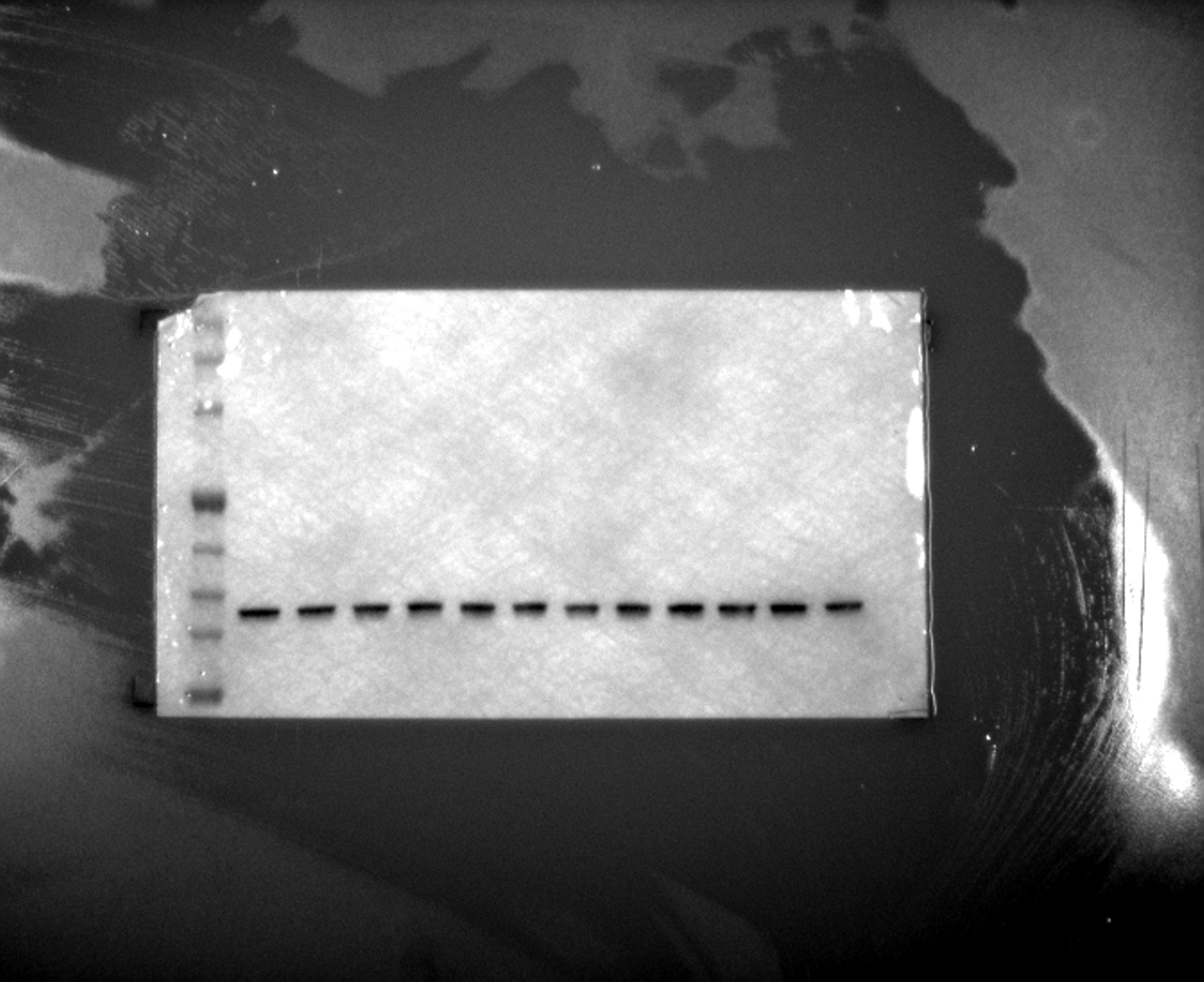

Supplement: Supplementary file 1 [file DataSheet3.ZIP › Figure 3/GAPDH_Gel-1.Tif]

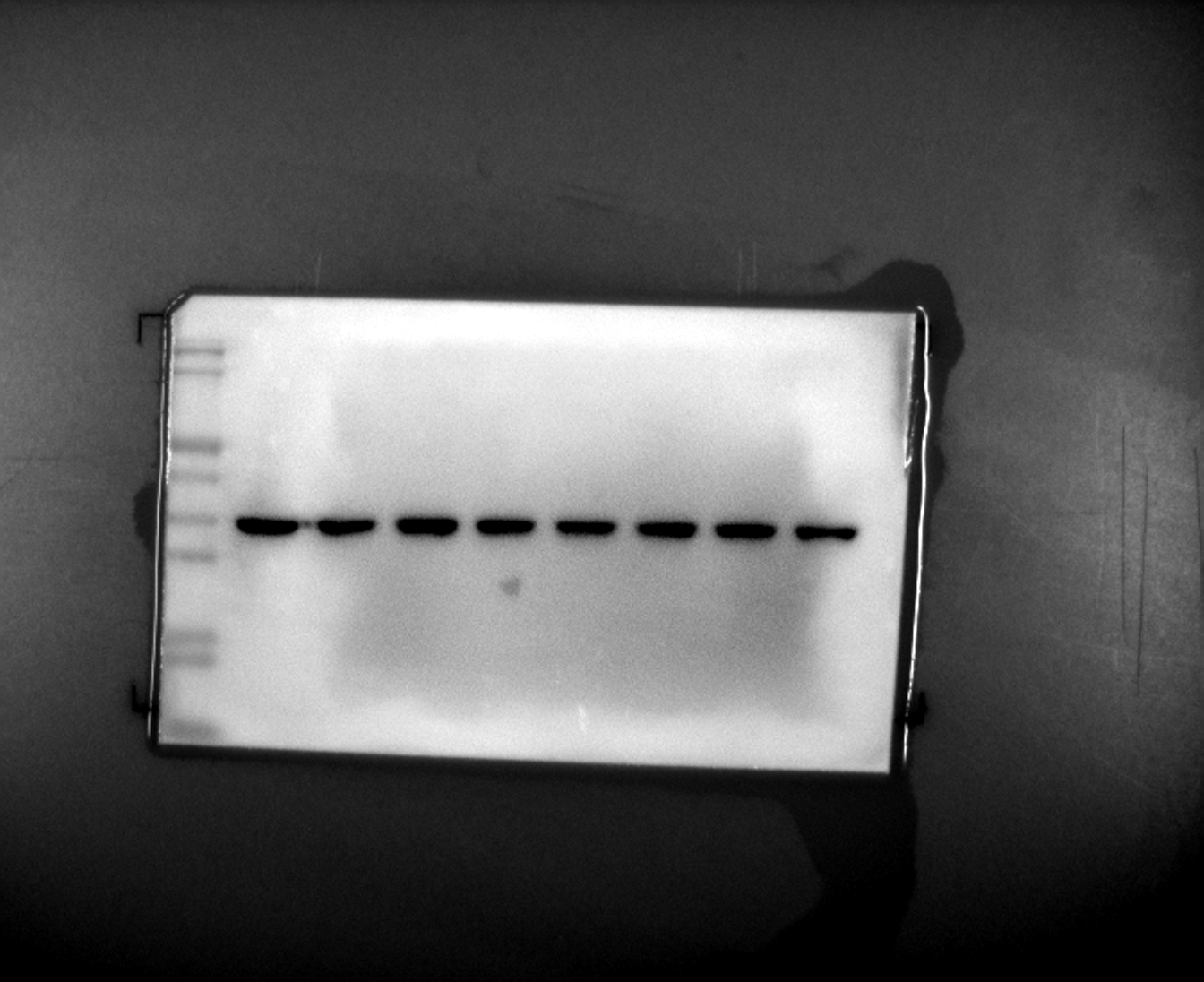

Supplement: Supplementary file 1 [file DataSheet3.ZIP › Figure 3/GAPDH_Gel-2.Tif]

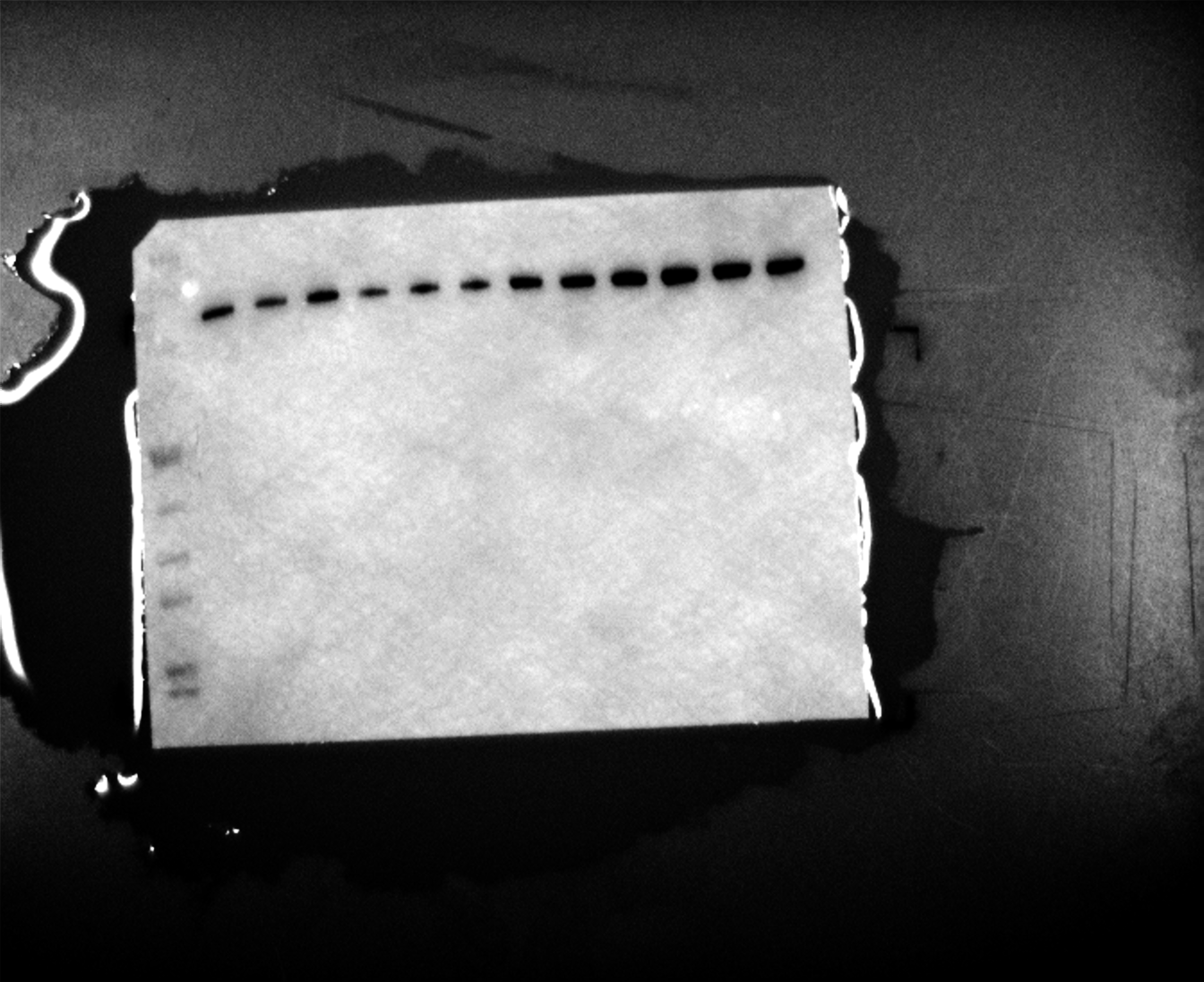

Supplement: Supplementary file 1 [file DataSheet3.ZIP › Figure 3/MUC-2_Gel-1.tif]

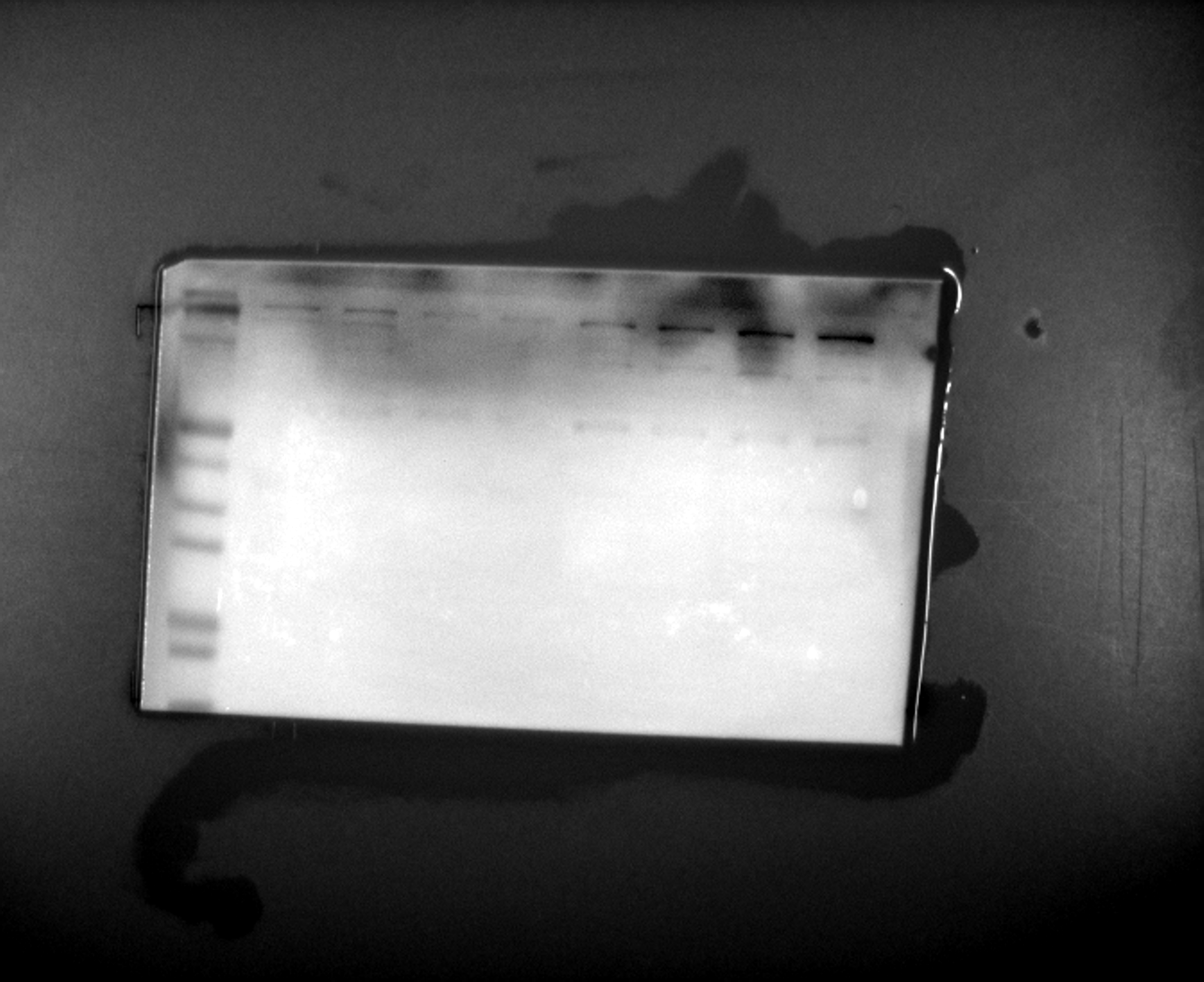

Supplement: Supplementary file 1 [file DataSheet3.ZIP › Figure 3/Muc-2_Gel-2.Tif]

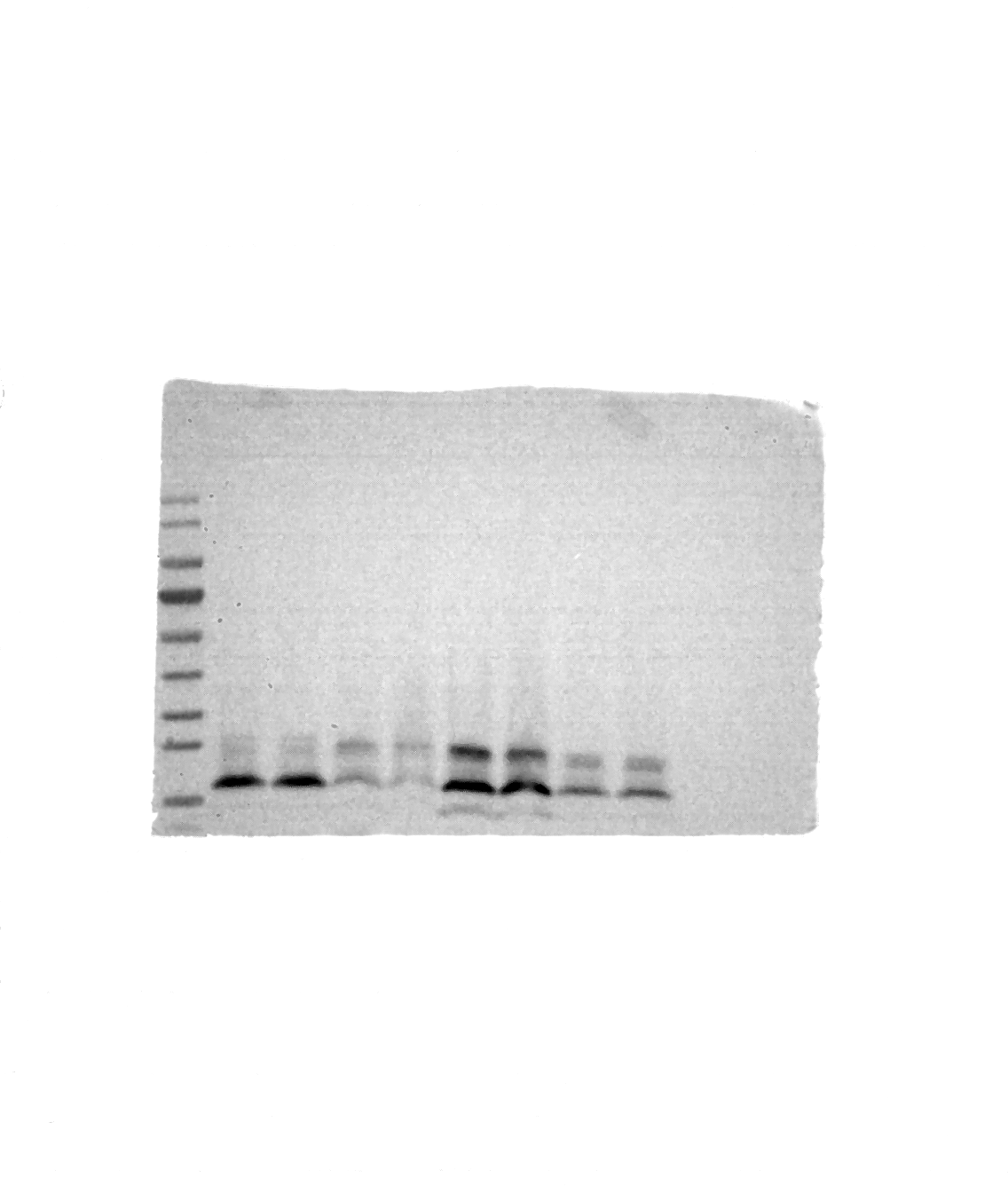

Supplement: Supplementary file 1 [file DataSheet3.ZIP › Figure 3/occludin-1_Gel-2.tif]

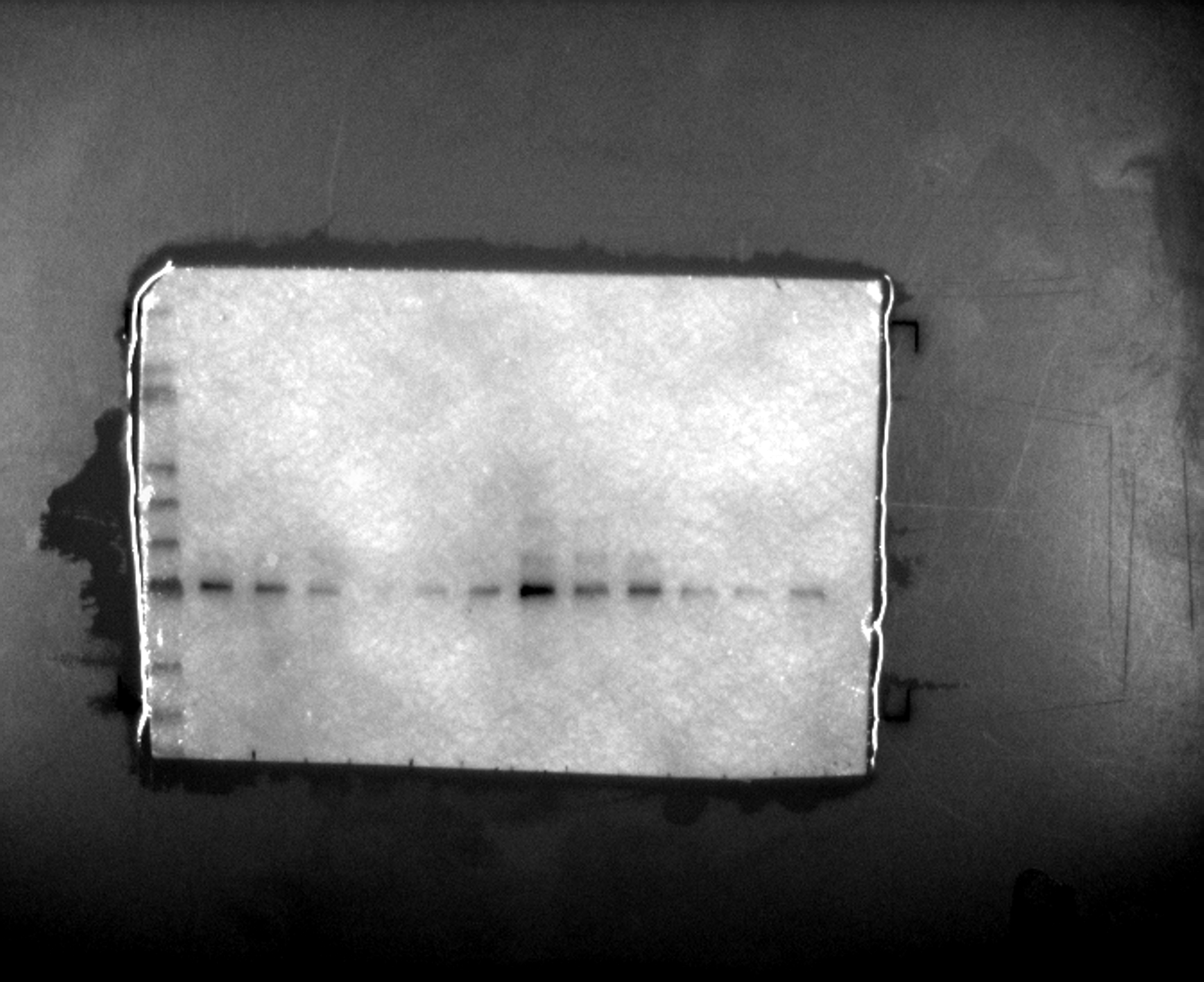

Supplement: Supplementary file 1 [file DataSheet3.ZIP › Figure 3/Occludin-2_Gel-1.Tif]

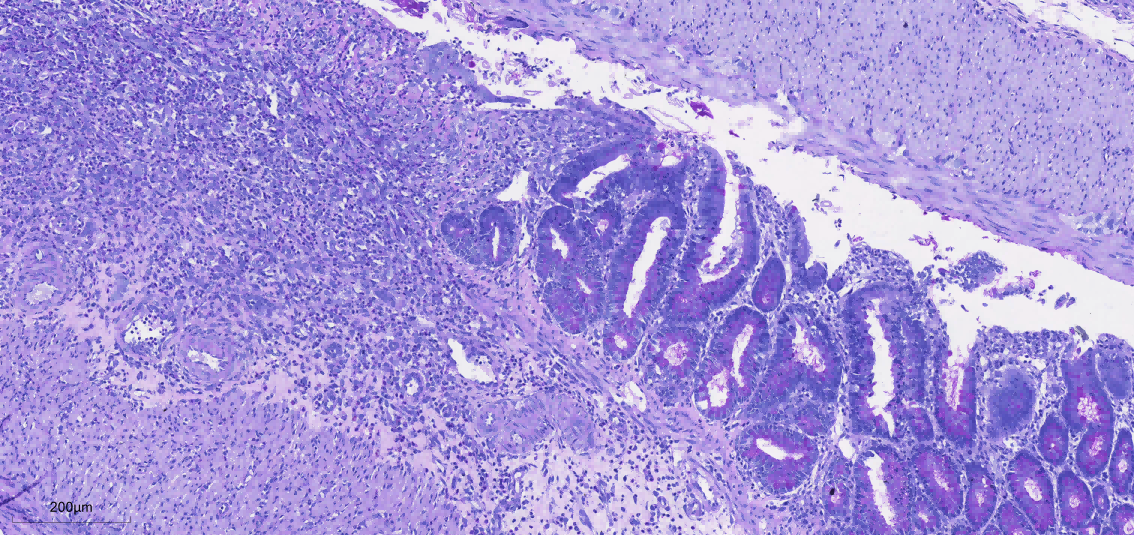

Supplement: Supplementary file 1 [file DataSheet3.ZIP › Figure 3/PAS Stain/HTP2 200a╠m.png]

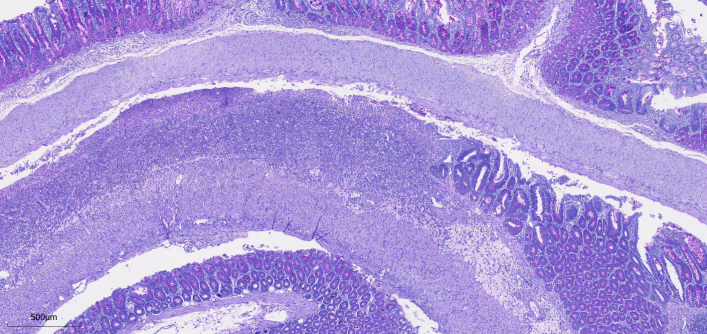

Supplement: Supplementary file 1 [file DataSheet3.ZIP › Figure 3/PAS Stain/HTP2 500a╠m.png]

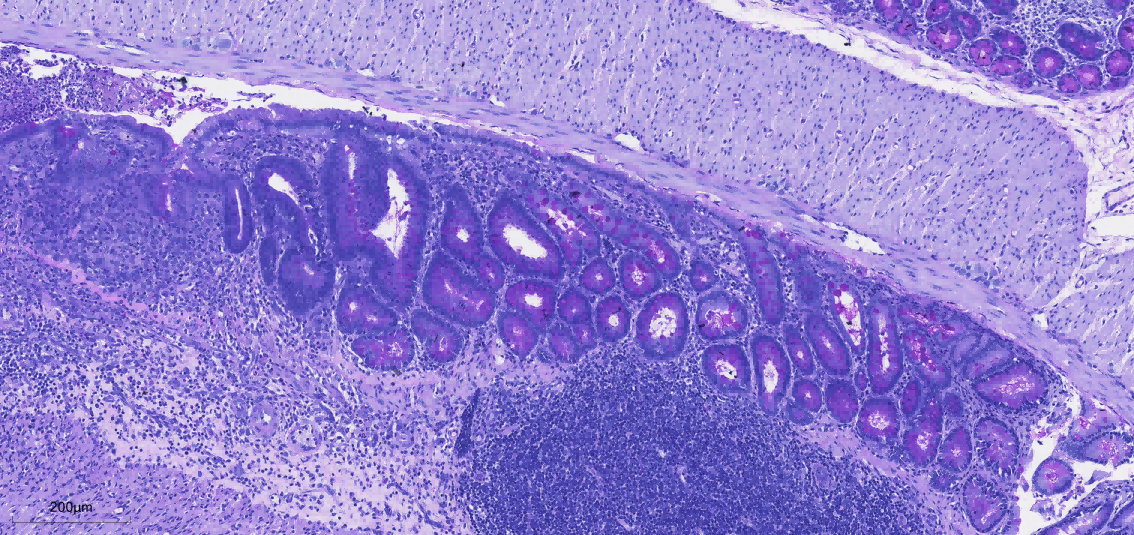

Supplement: Supplementary file 1 [file DataSheet3.ZIP › Figure 3/PAS Stain/LTP2 200a╠m.png]

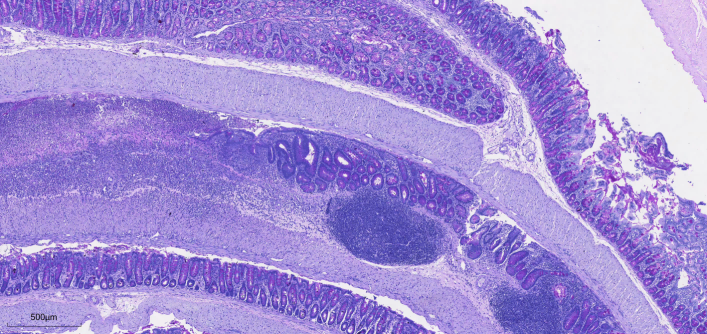

Supplement: Supplementary file 1 [file DataSheet3.ZIP › Figure 3/PAS Stain/LTP2 500a╠m.png]

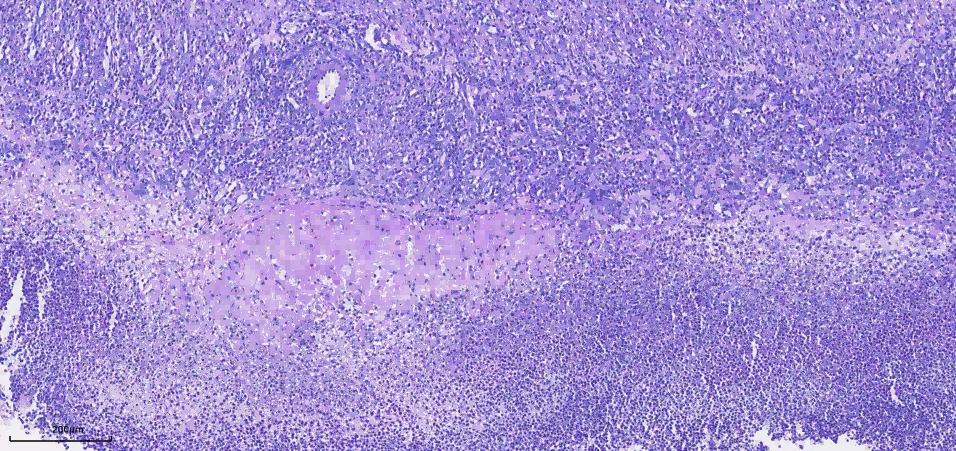

Supplement: Supplementary file 1 [file DataSheet3.ZIP › Figure 3/PAS Stain/MC 200a╠m.png]

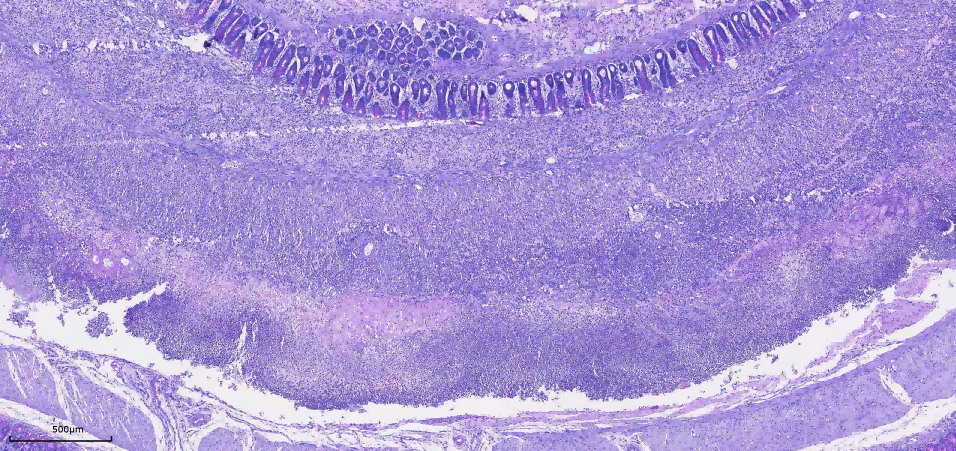

Supplement: Supplementary file 1 [file DataSheet3.ZIP › Figure 3/PAS Stain/MC 500a╠m.png]

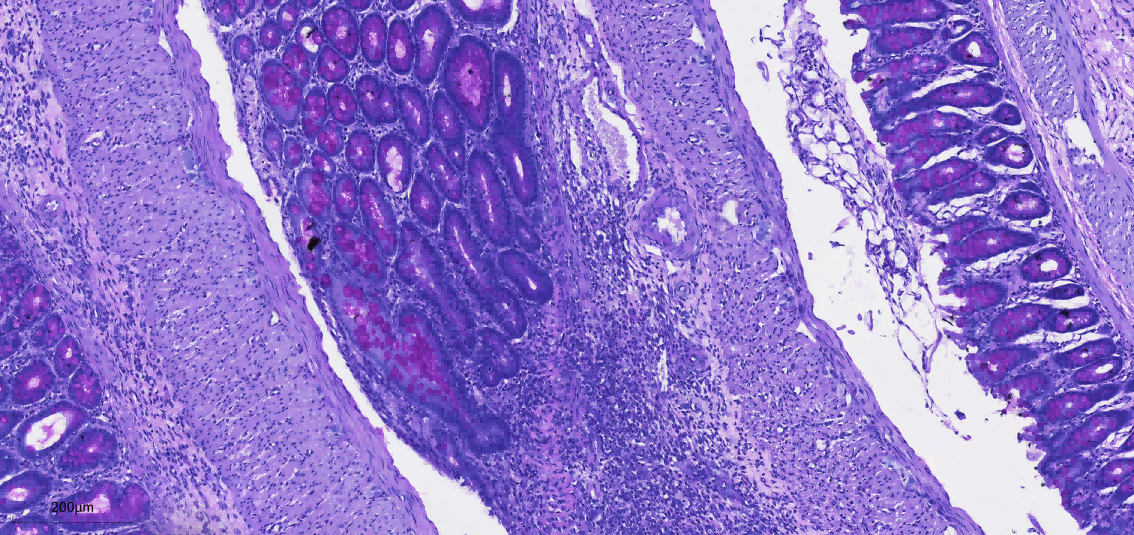

Supplement: Supplementary file 1 [file DataSheet3.ZIP › Figure 3/PAS Stain/MTP2 200a╠m.png]

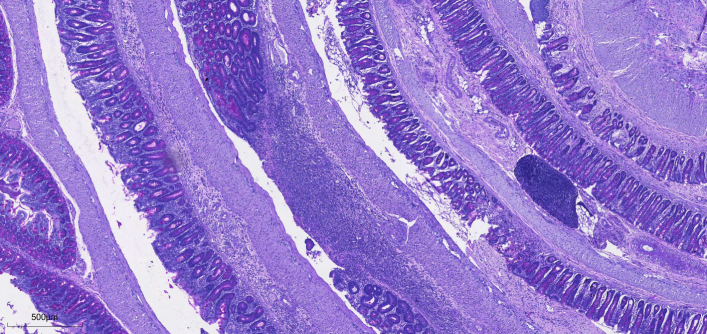

Supplement: Supplementary file 1 [file DataSheet3.ZIP › Figure 3/PAS Stain/MTP2 500a╠m.png]

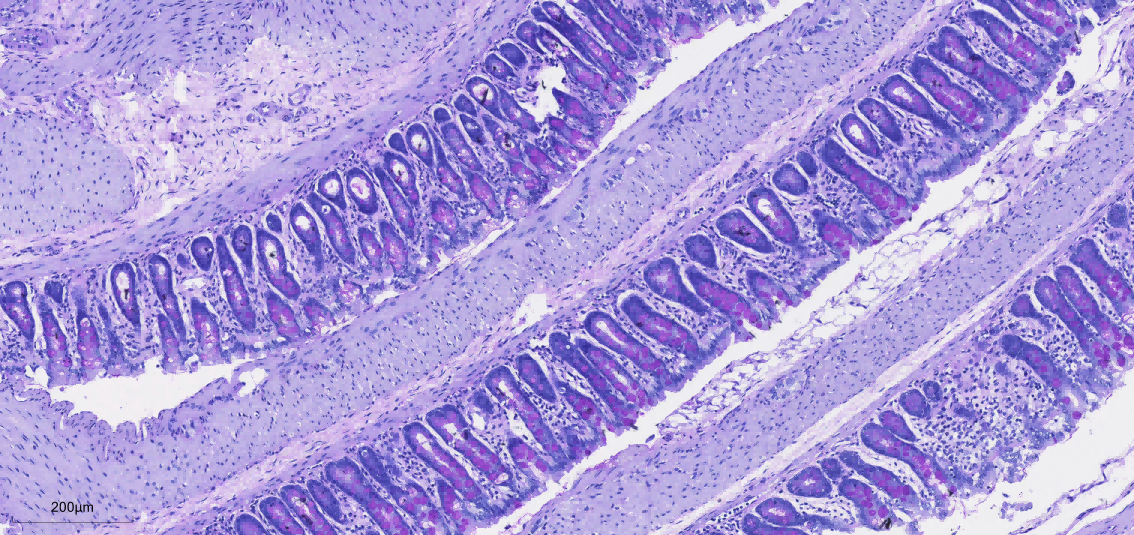

Supplement: Supplementary file 1 [file DataSheet3.ZIP › Figure 3/PAS Stain/NC 200a╠m.png]

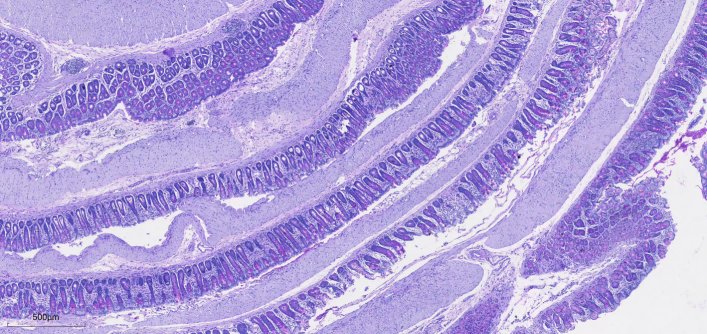

Supplement: Supplementary file 1 [file DataSheet3.ZIP › Figure 3/PAS Stain/NC 500a╠m.png]

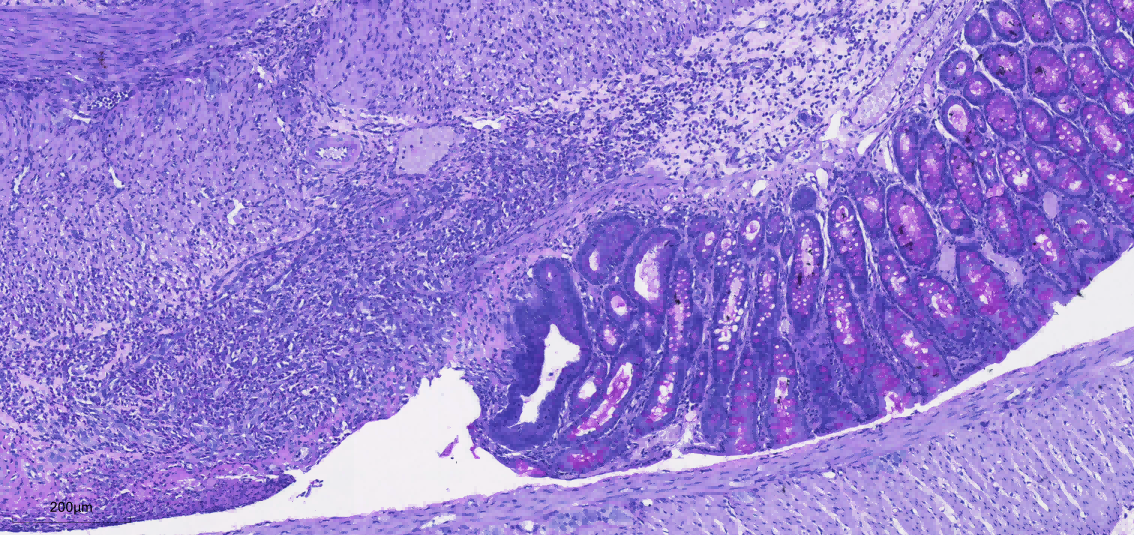

Supplement: Supplementary file 1 [file DataSheet3.ZIP › Figure 3/PAS Stain/PD 200a╠m.png]

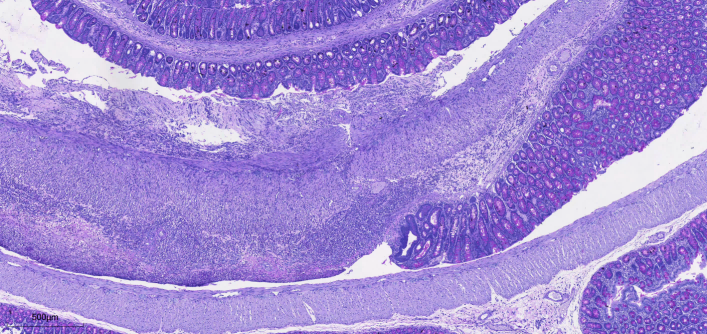

Supplement: Supplementary file 1 [file DataSheet3.ZIP › Figure 3/PAS Stain/PD 500a╠m.png]

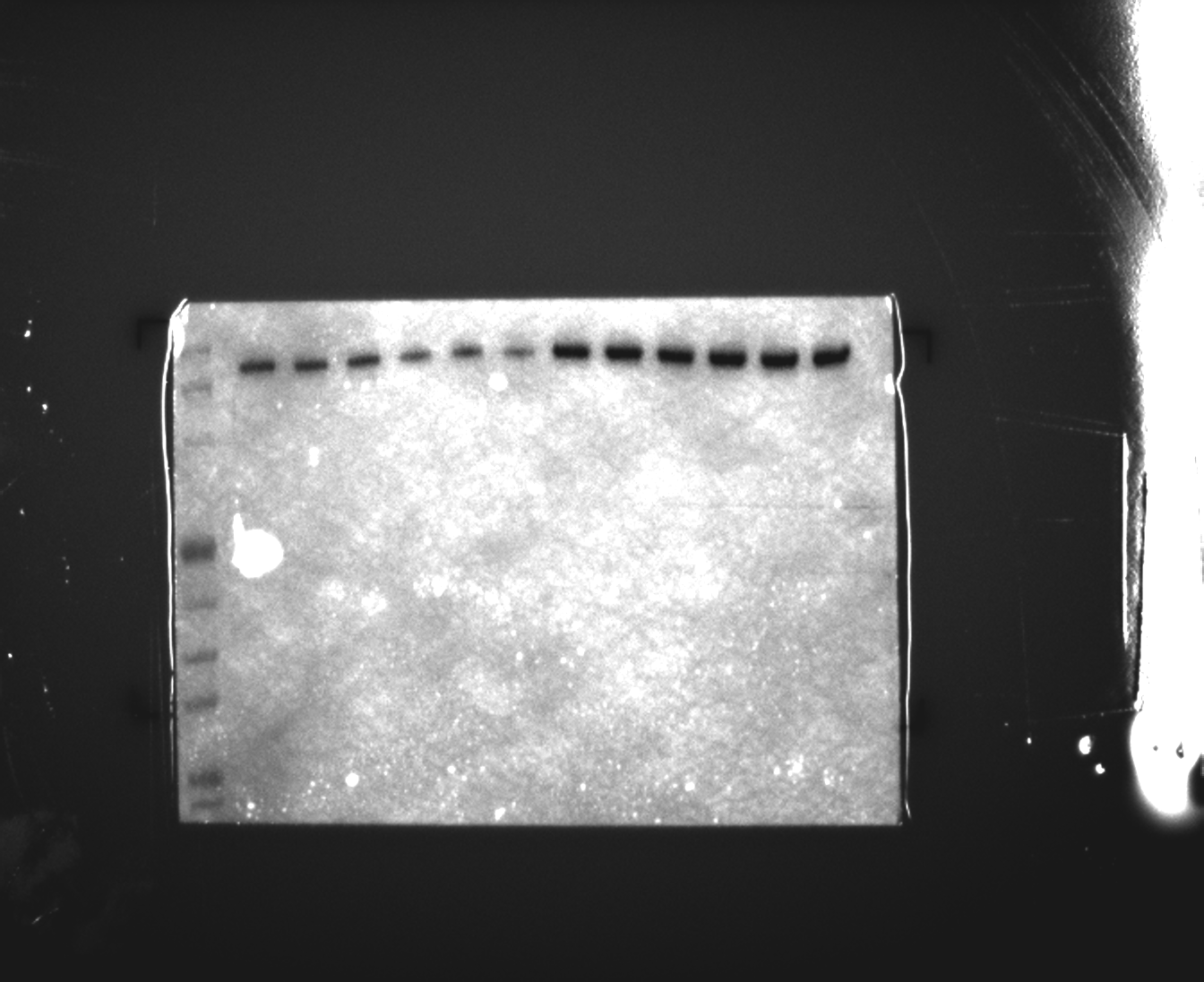

Supplement: Supplementary file 1 [file DataSheet3.ZIP › Figure 3/ZO-1_Gel-1.tif]

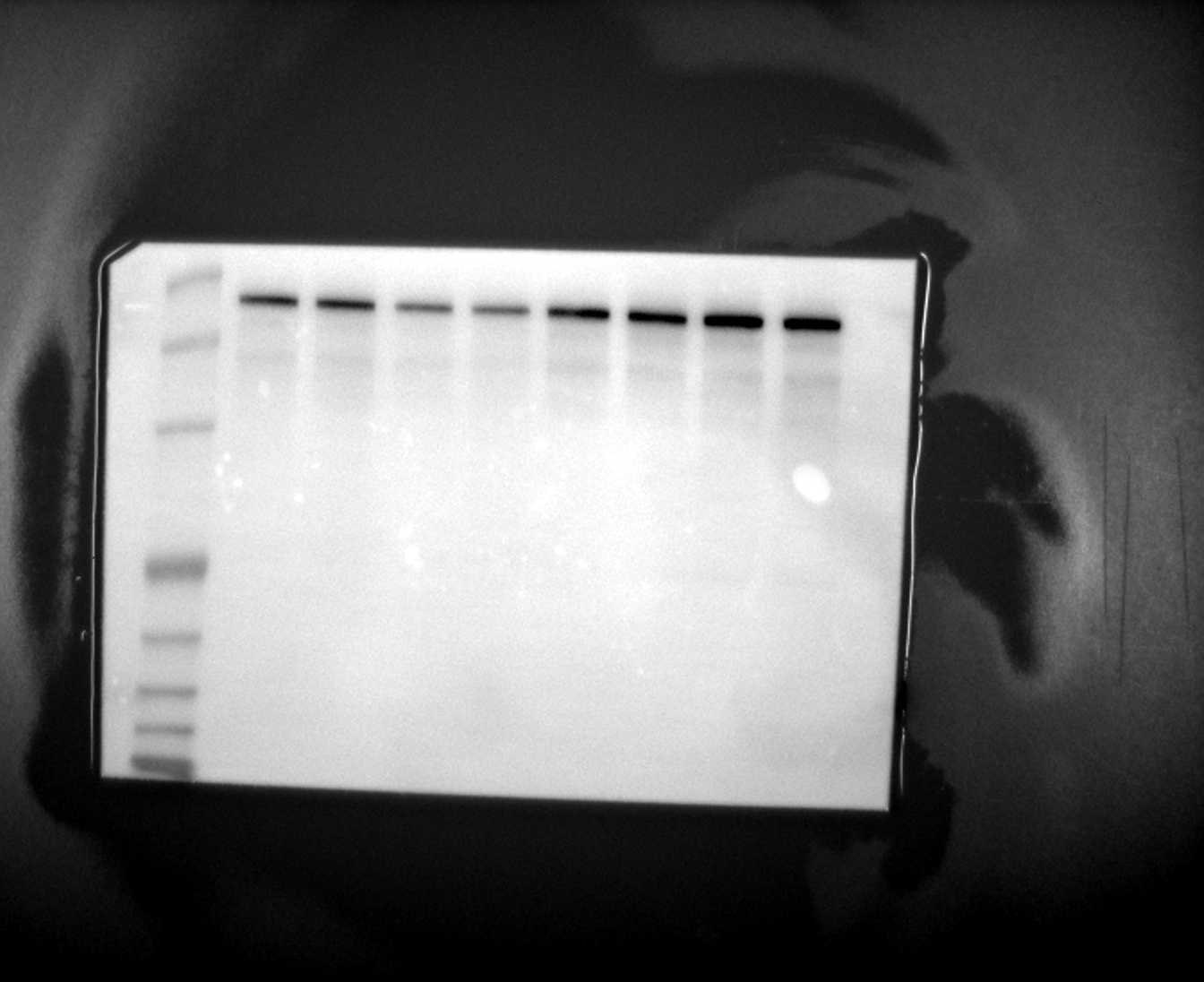

Supplement: Supplementary file 1 [file DataSheet3.ZIP › Figure 3/ZO-1_Gel-2.Tif]

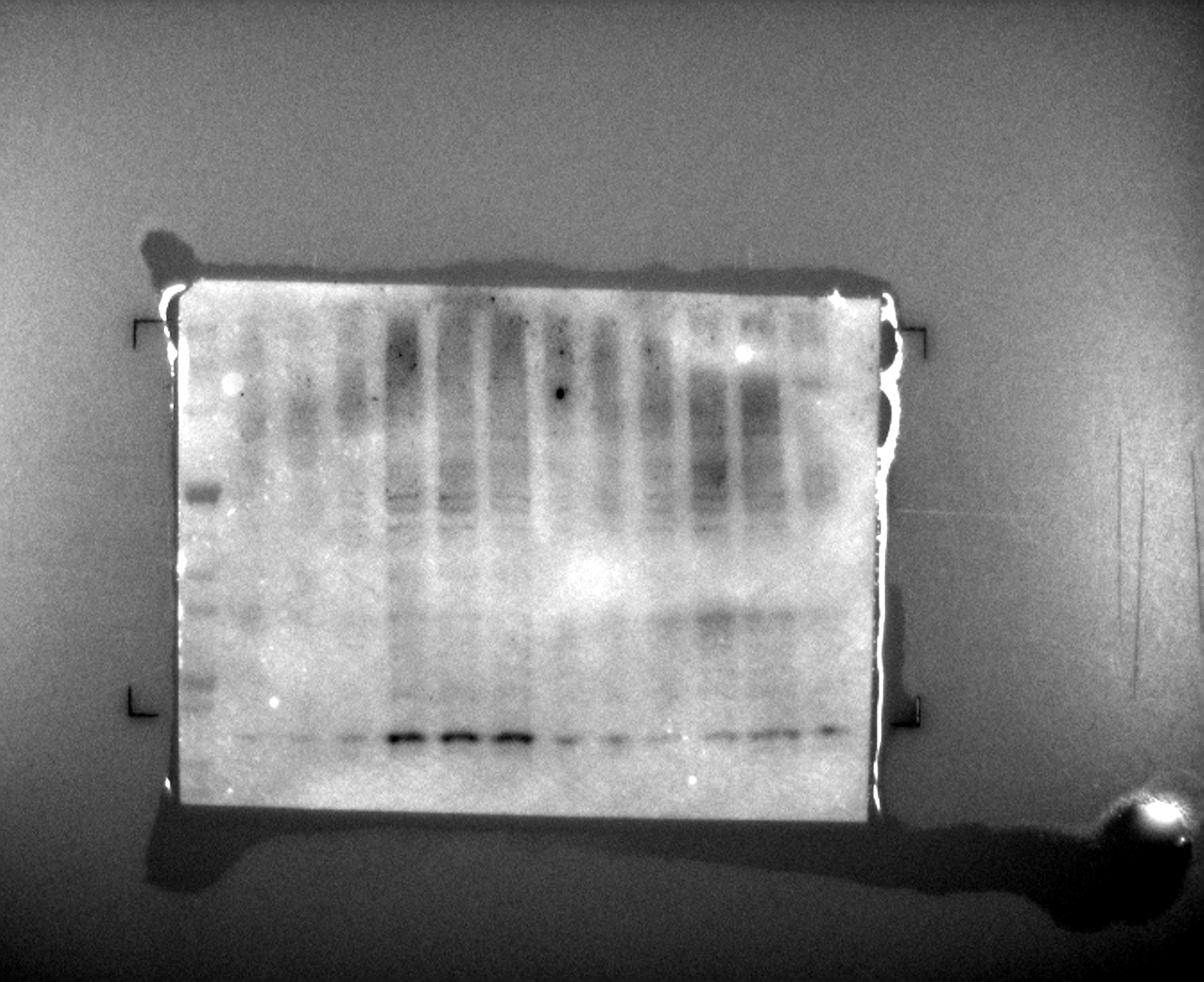

Supplement: Supplementary file 2 [file DataSheet4.ZIP › Figure 4/BAX_Gel-1.Tif]

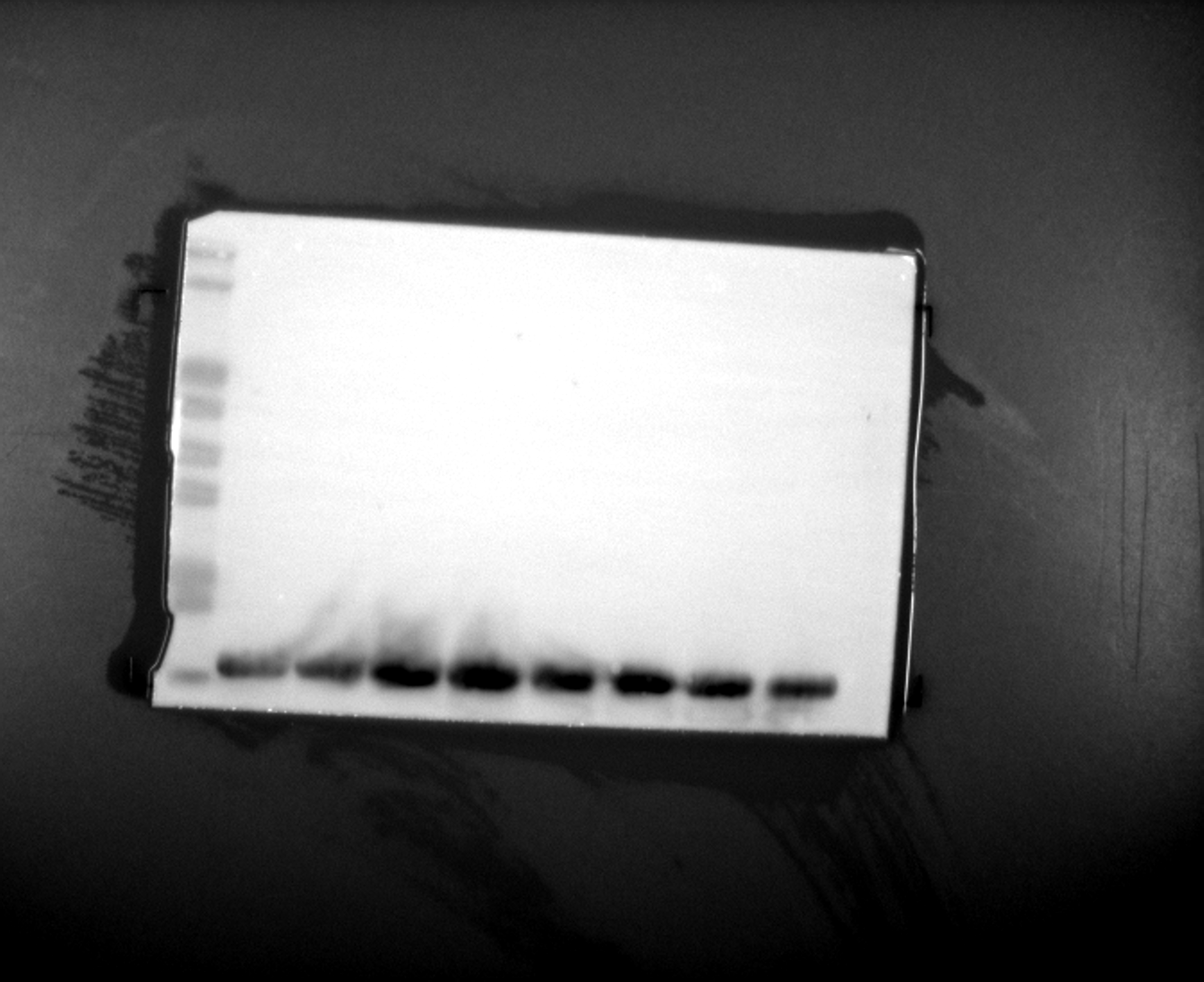

Supplement: Supplementary file 2 [file DataSheet4.ZIP › Figure 4/BAX_Gel-2.Tif]

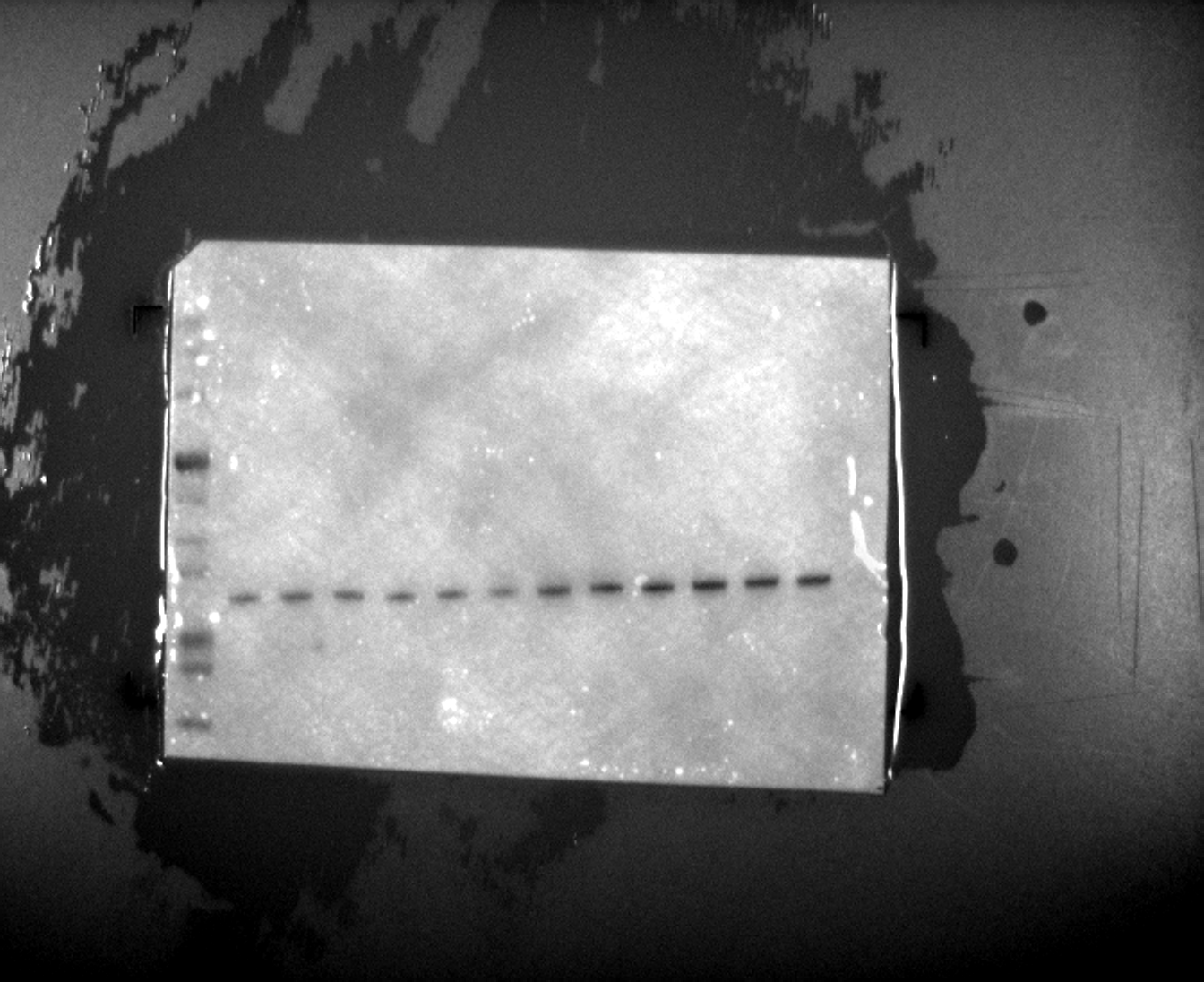

Supplement: Supplementary file 2 [file DataSheet4.ZIP › Figure 4/Bcl-2_Gel-1.Tif]

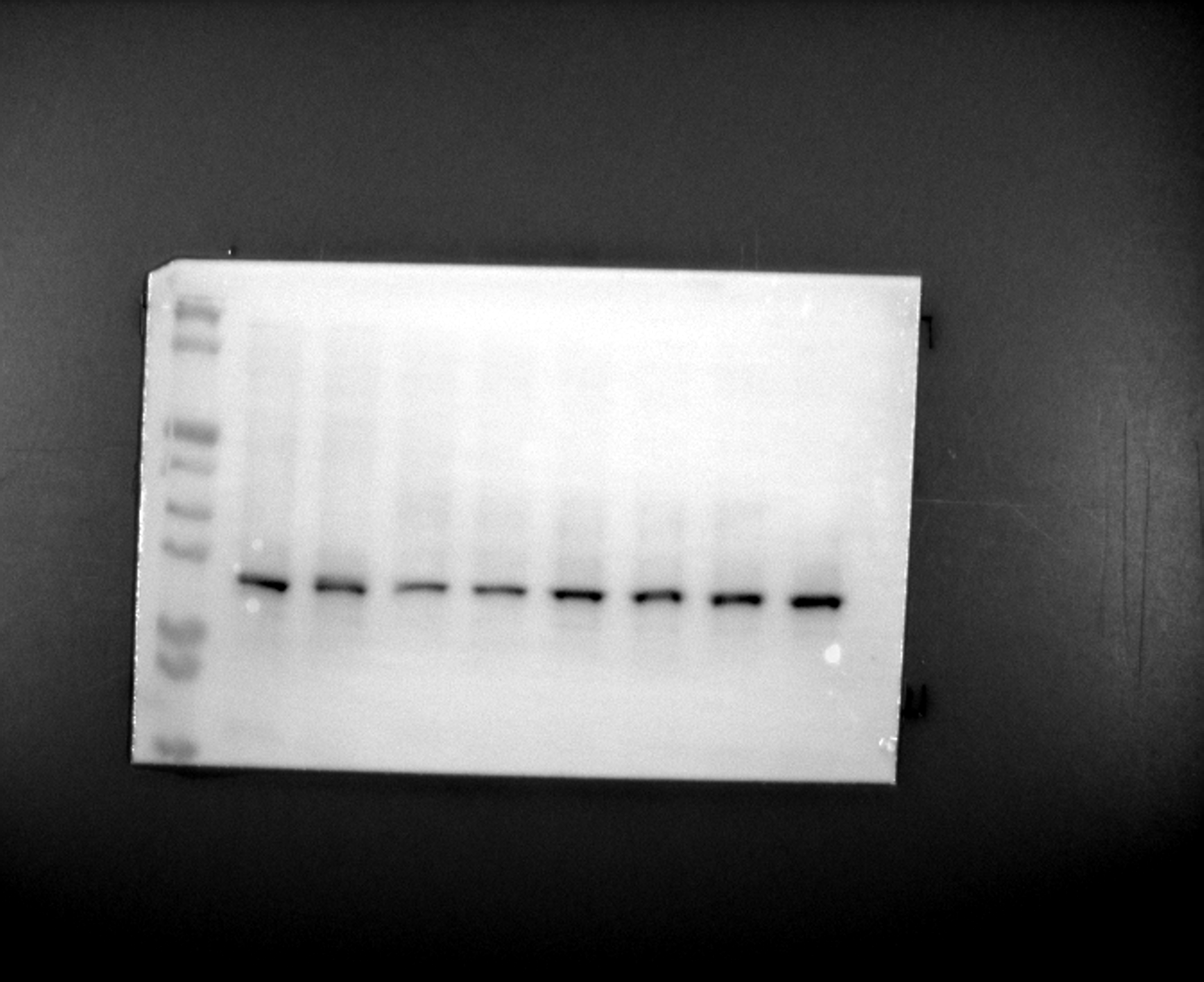

Supplement: Supplementary file 2 [file DataSheet4.ZIP › Figure 4/Bcl-2_Gel-2.Tif]

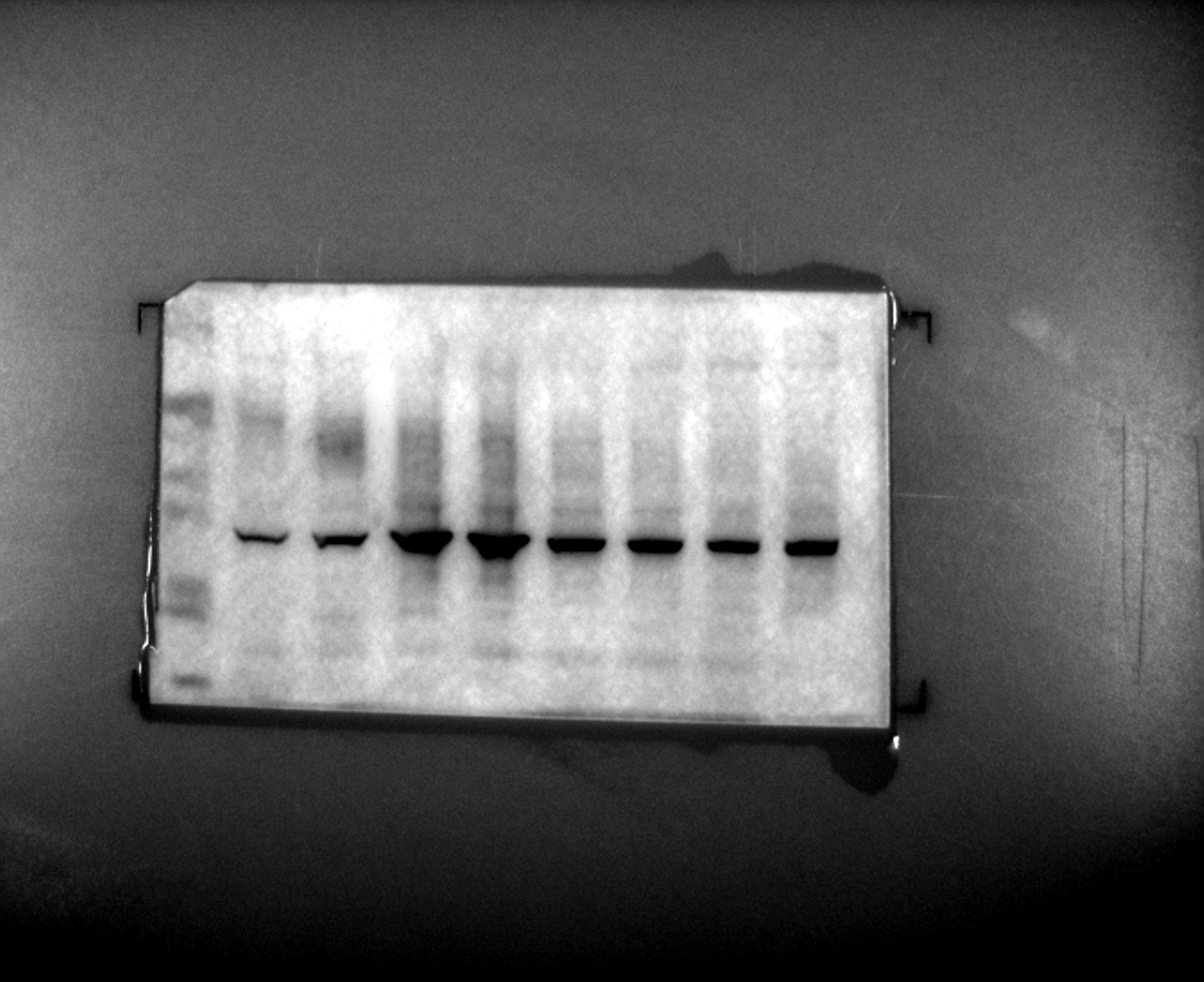

Supplement: Supplementary file 2 [file DataSheet4.ZIP › Figure 4/Cleaved Caspase-3_Gel-2.Tif]

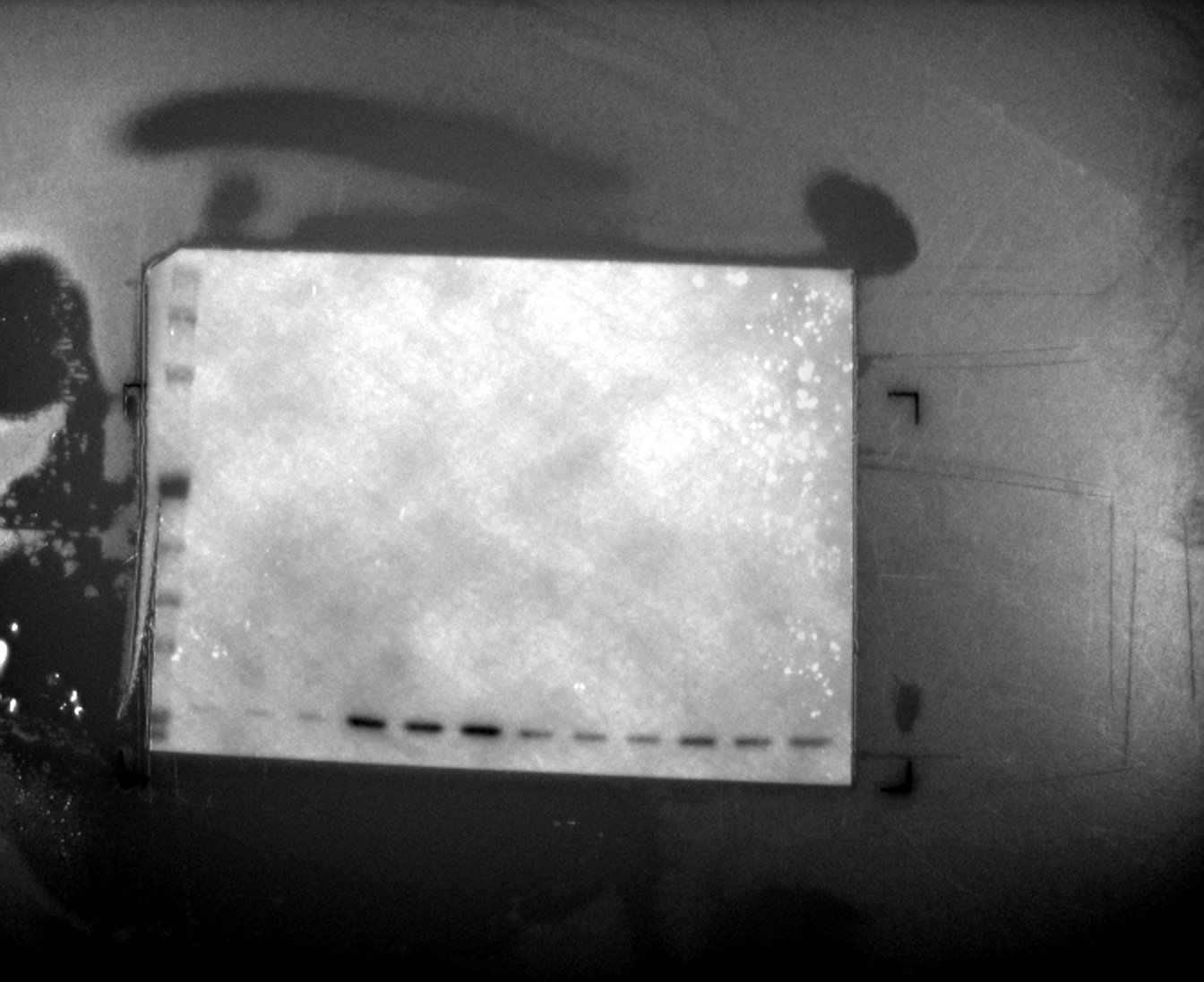

Supplement: Supplementary file 2 [file DataSheet4.ZIP › Figure 4/Cleaved-Caspase-3_Gel-1.Tif]

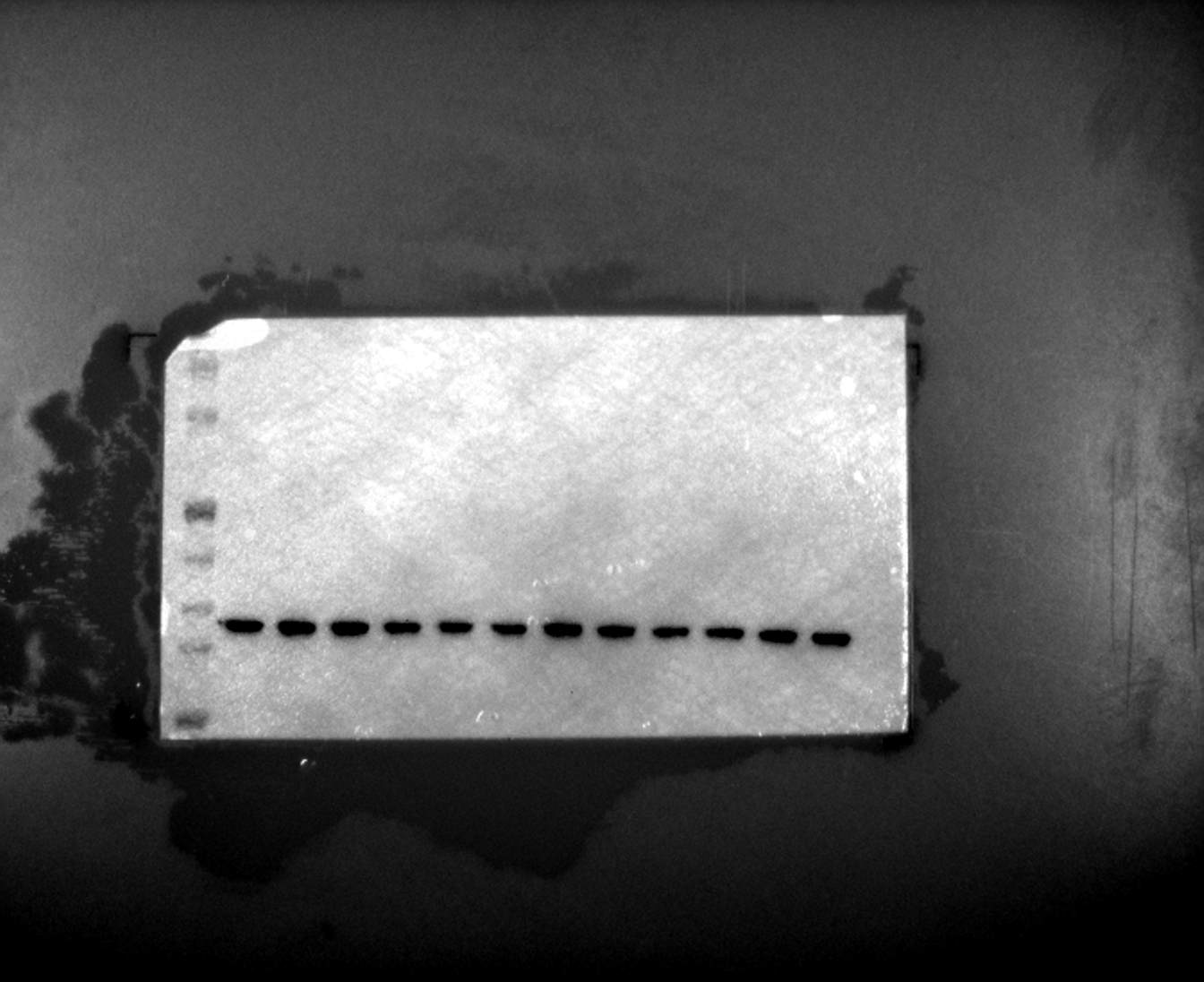

Supplement: Supplementary file 2 [file DataSheet4.ZIP › Figure 4/GAPDH_Gel-1.Tif]

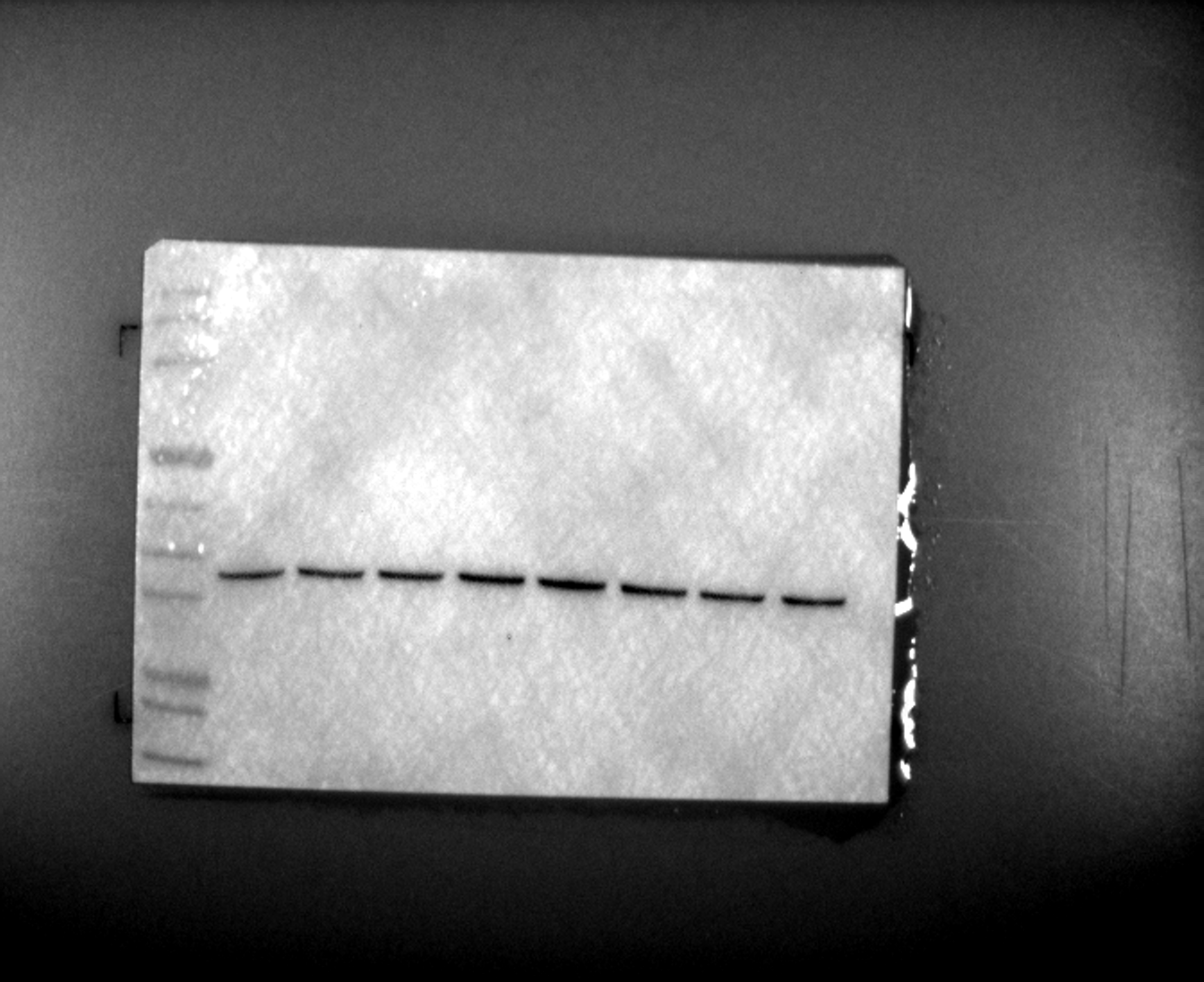

Supplement: Supplementary file 2 [file DataSheet4.ZIP › Figure 4/GAPDH_Gel-2.Tif]

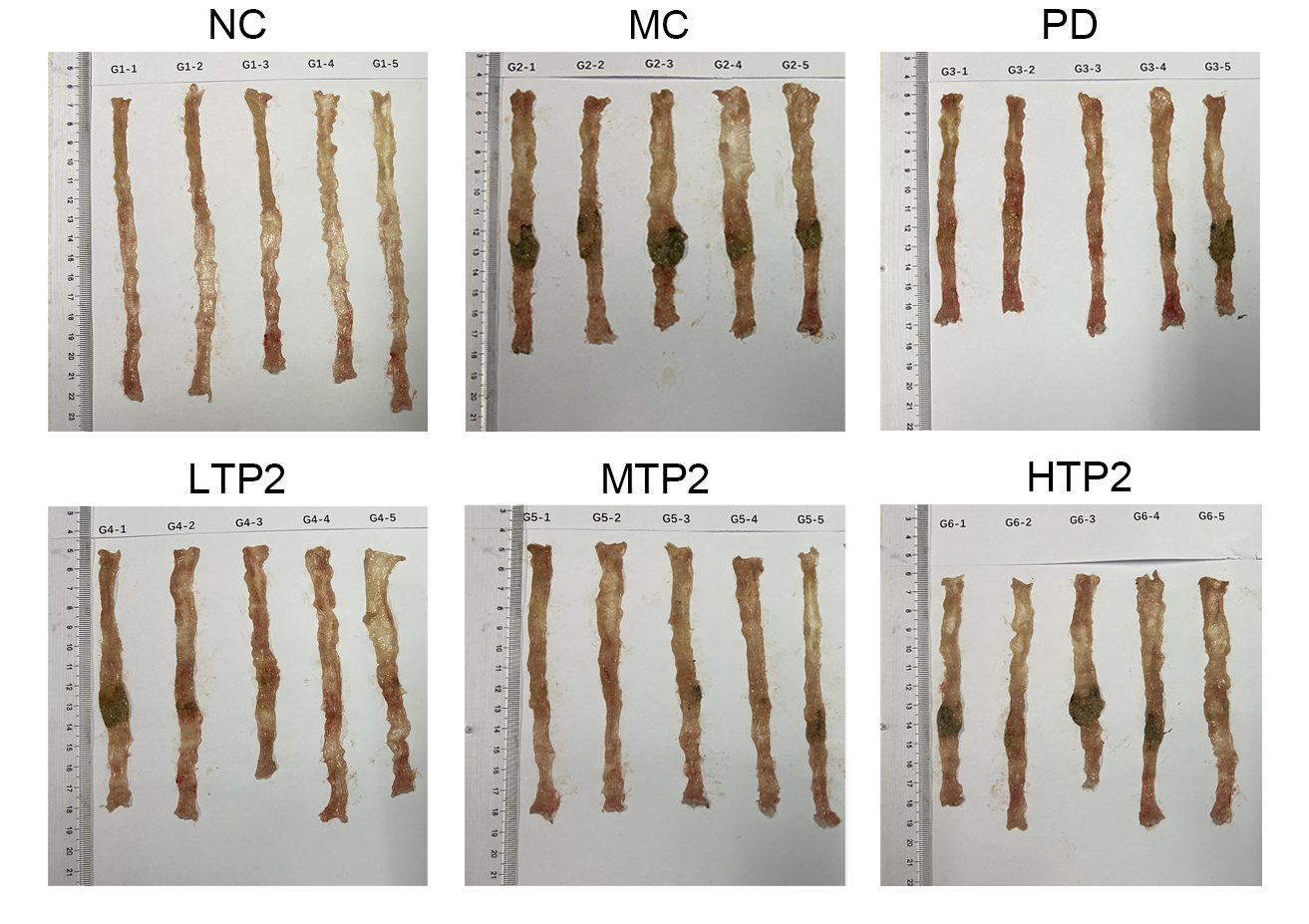

Supplement: Supplementary file 3 [file DataSheet1.ZIP › Figure 1/Ulcer Photography.tif]

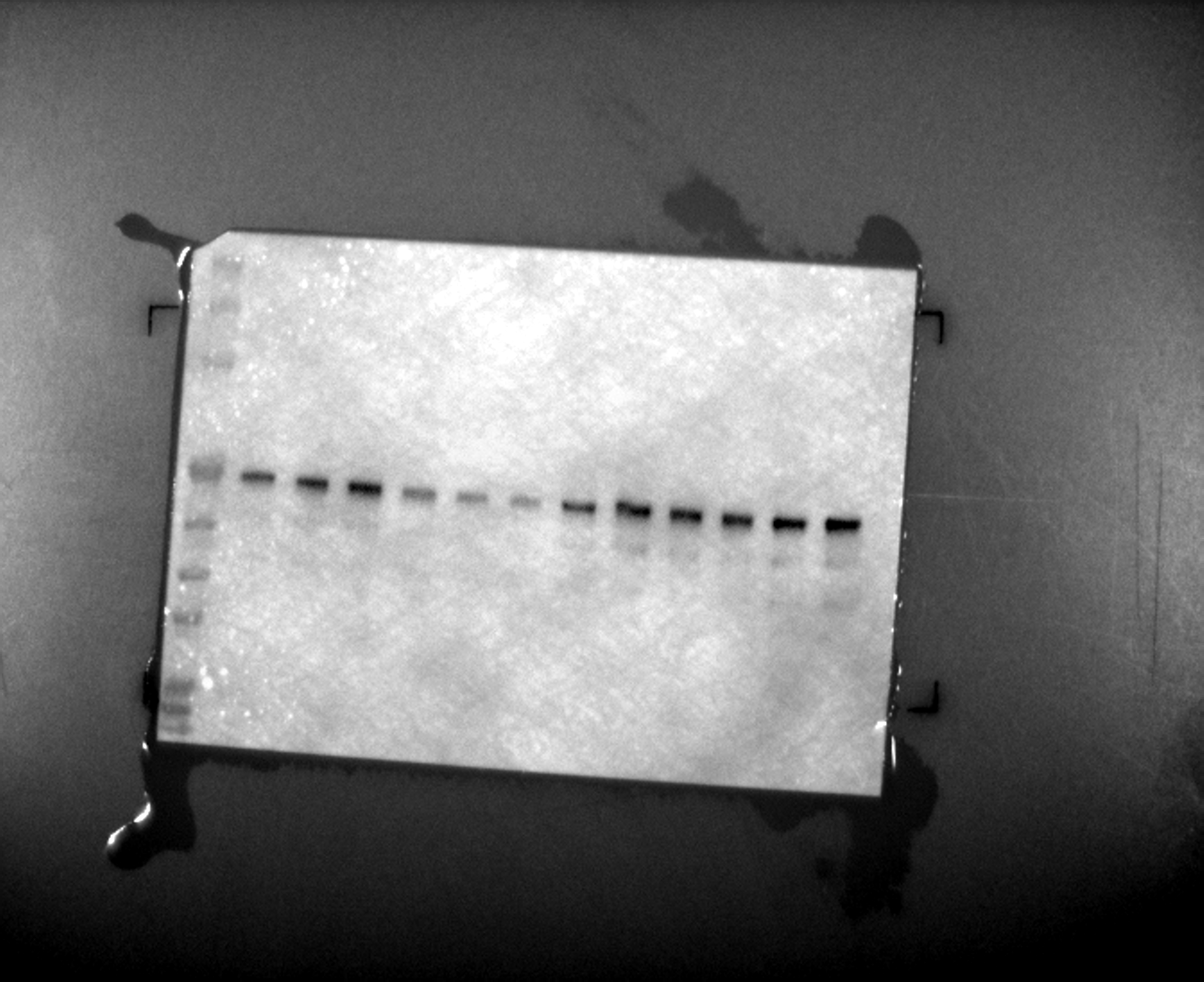

Supplement: Supplementary file 4 [file DataSheet6.ZIP › Figure 6/AKT_Gel-1.Tif]

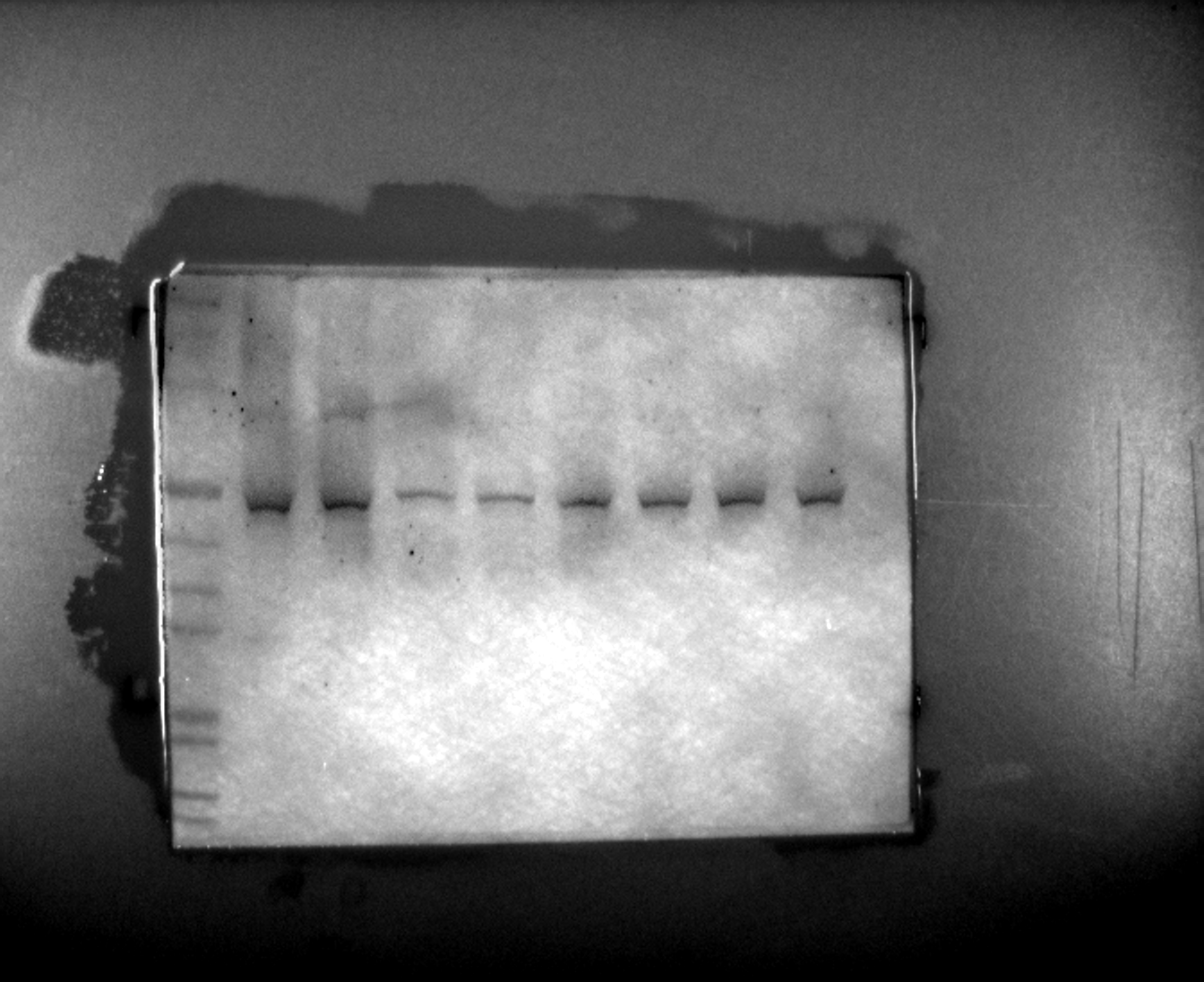

Supplement: Supplementary file 4 [file DataSheet6.ZIP › Figure 6/AKT_Gel-2.Tif]

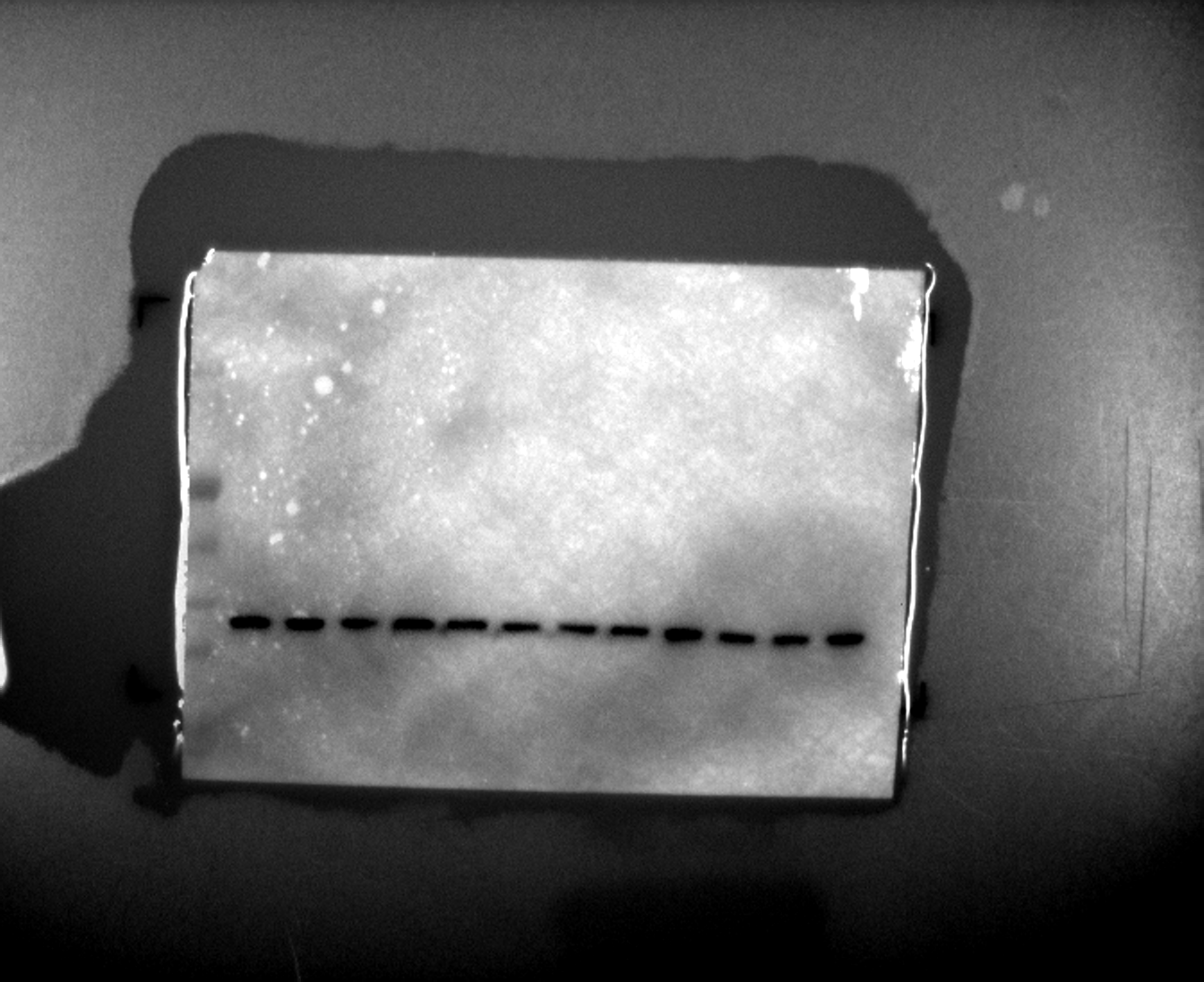

Supplement: Supplementary file 4 [file DataSheet6.ZIP › Figure 6/GAPDH_Gel-1.Tif]

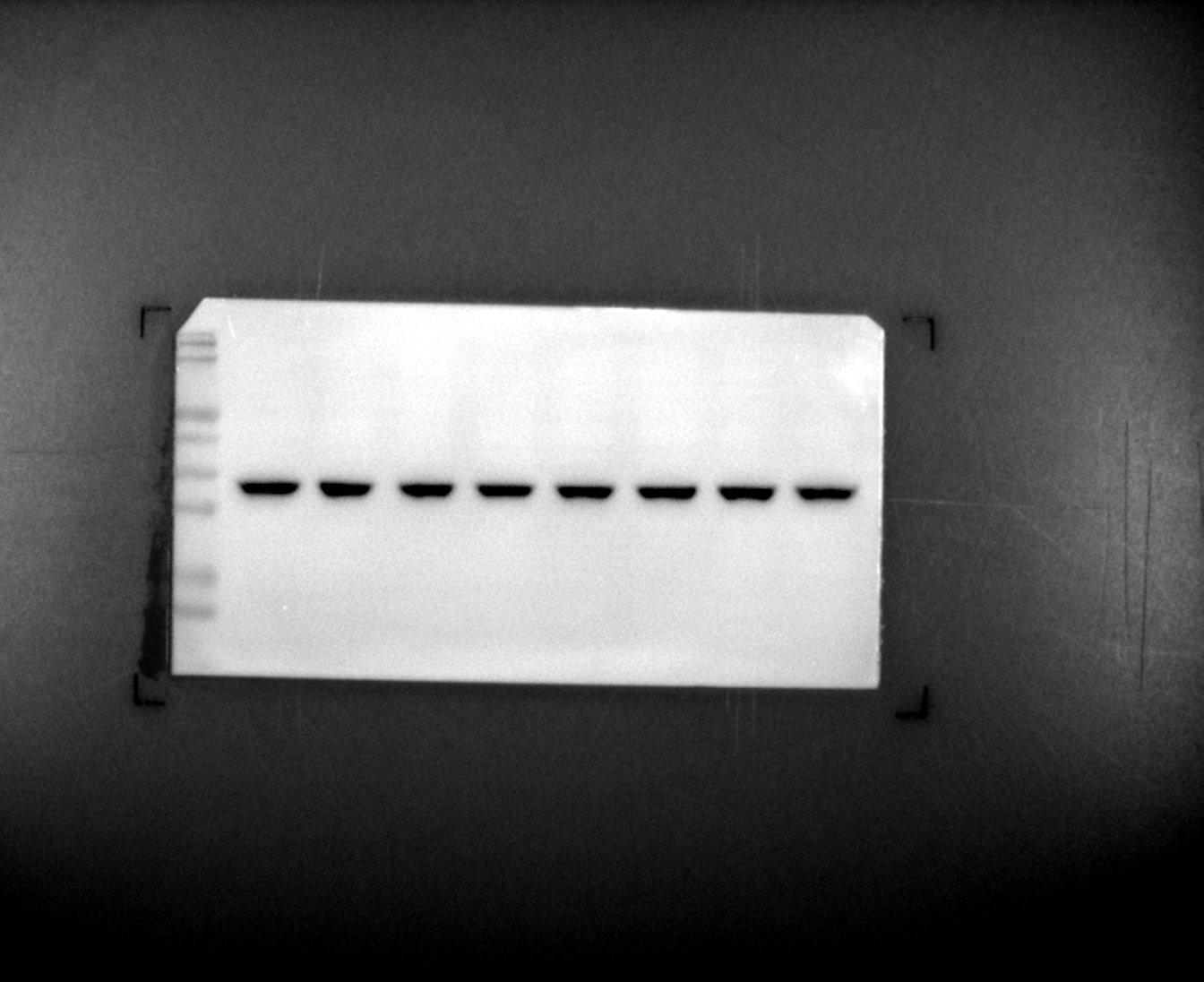

Supplement: Supplementary file 4 [file DataSheet6.ZIP › Figure 6/GAPDH_Gel-2.Tif]

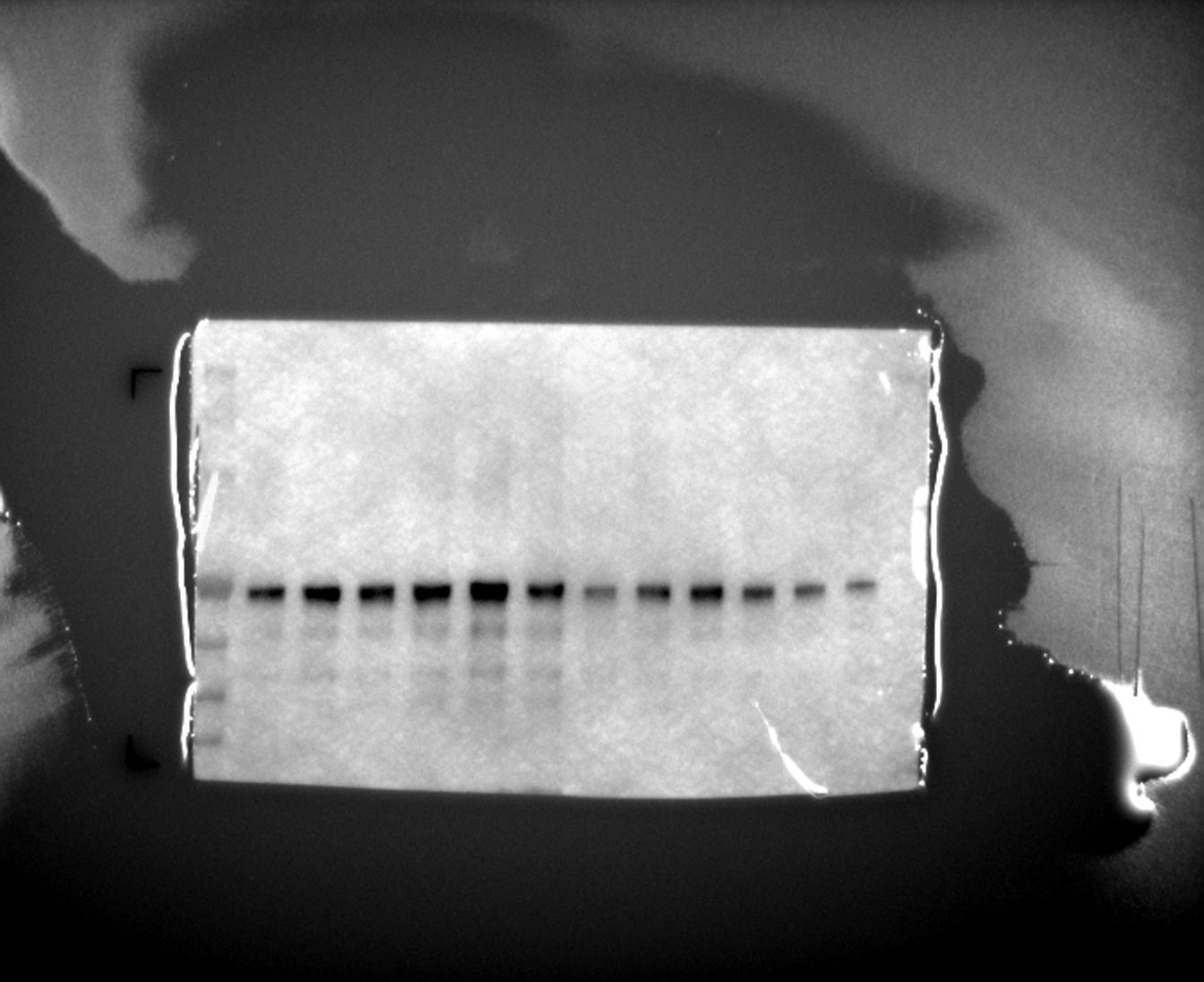

Supplement: Supplementary file 4 [file DataSheet6.ZIP › Figure 6/p-AKT_Gel-1.Tif]

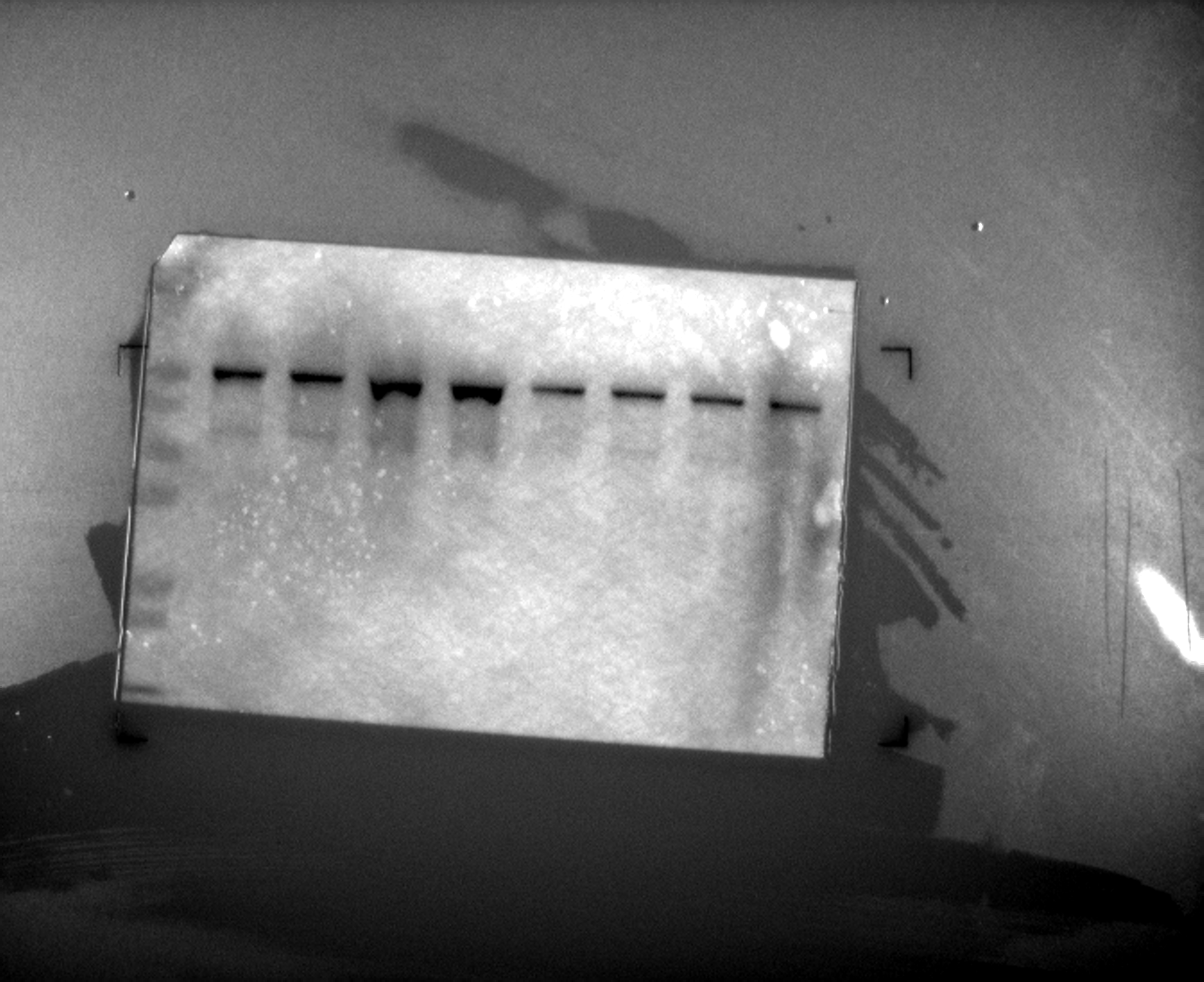

Supplement: Supplementary file 4 [file DataSheet6.ZIP › Figure 6/p-AKT_Gel-2.Tif]

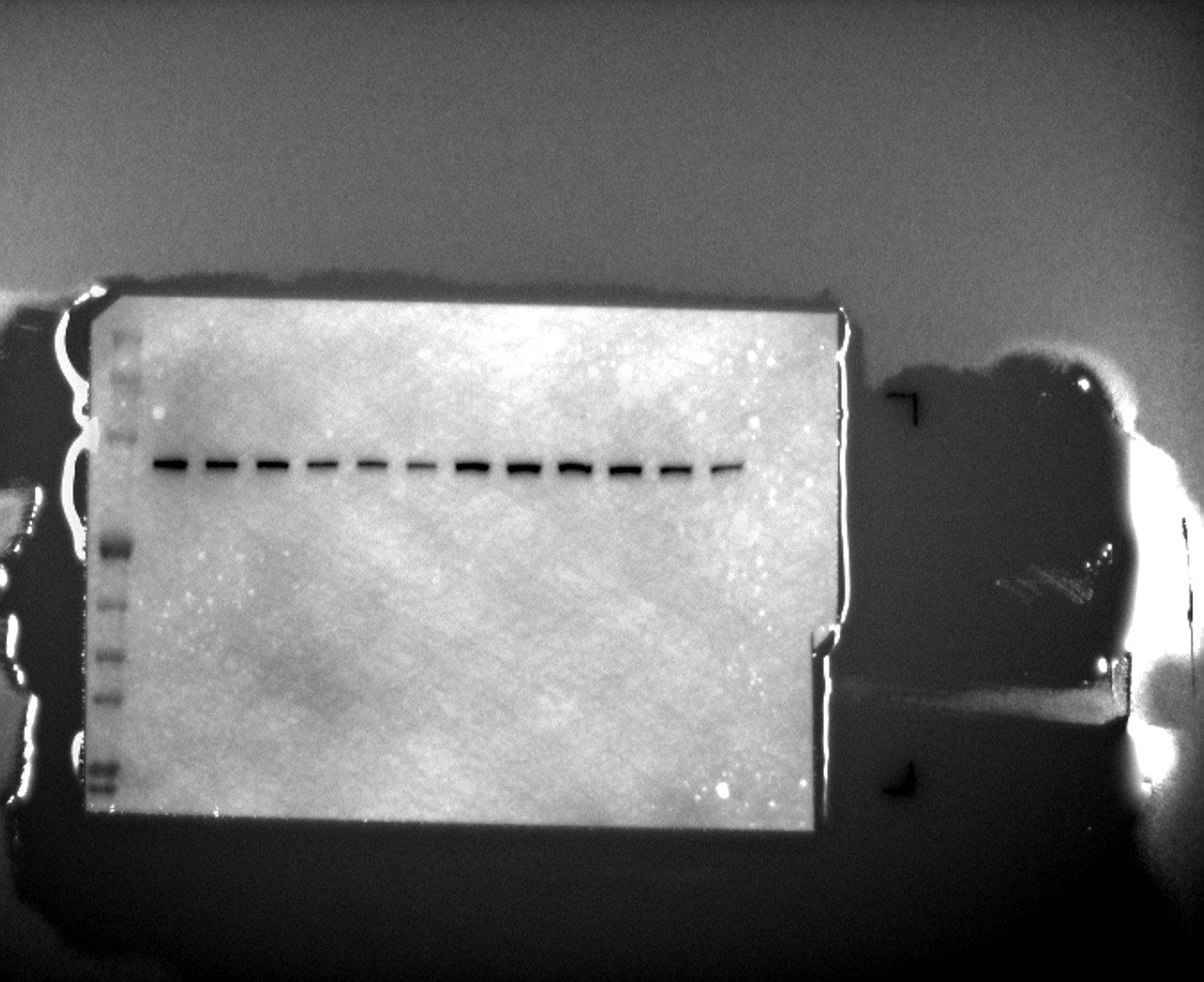

Supplement: Supplementary file 4 [file DataSheet6.ZIP › Figure 6/PI3K_Gel-1.Tif]

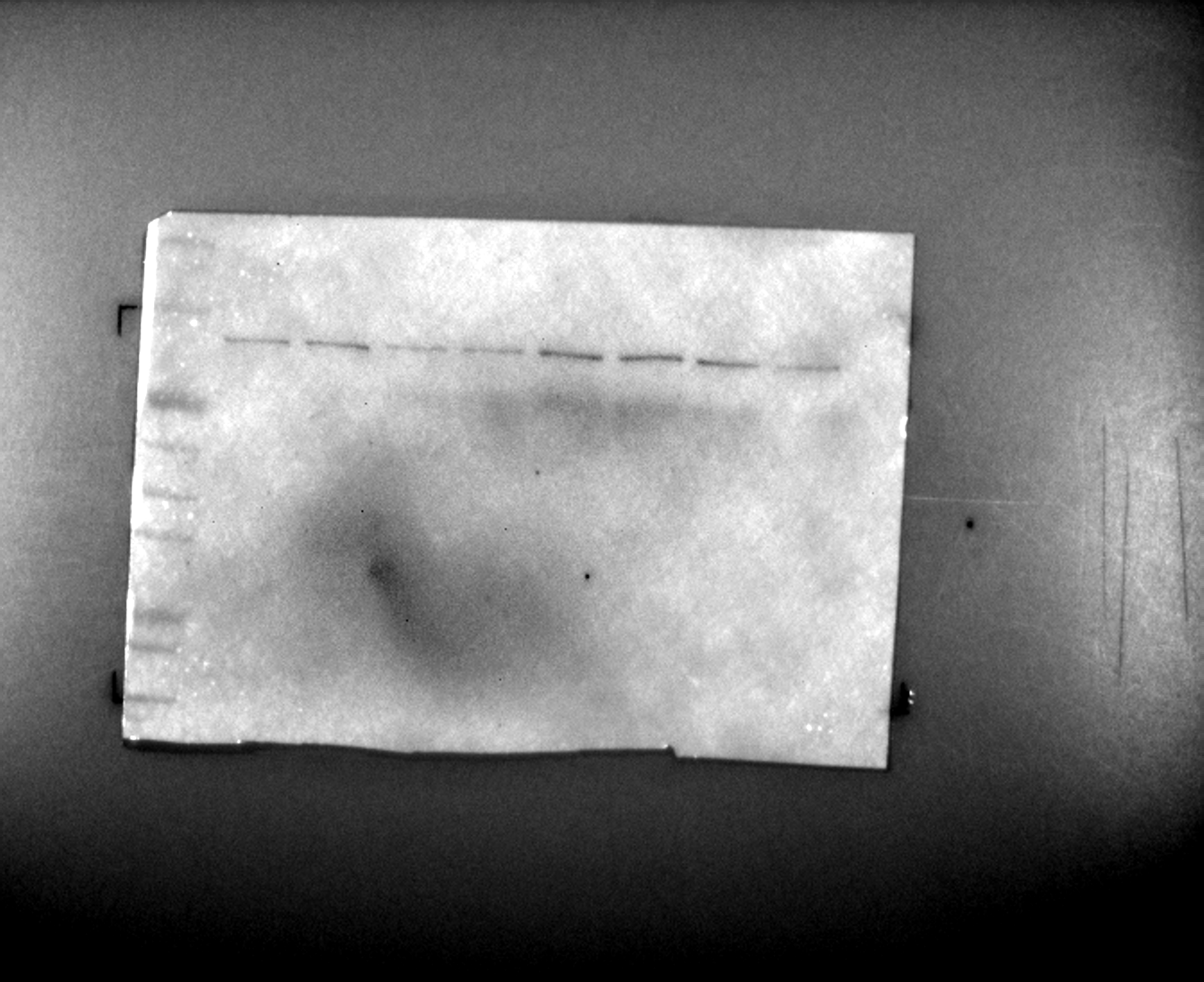

Supplement: Supplementary file 4 [file DataSheet6.ZIP › Figure 6/PI3K_Gel-2.Tif]

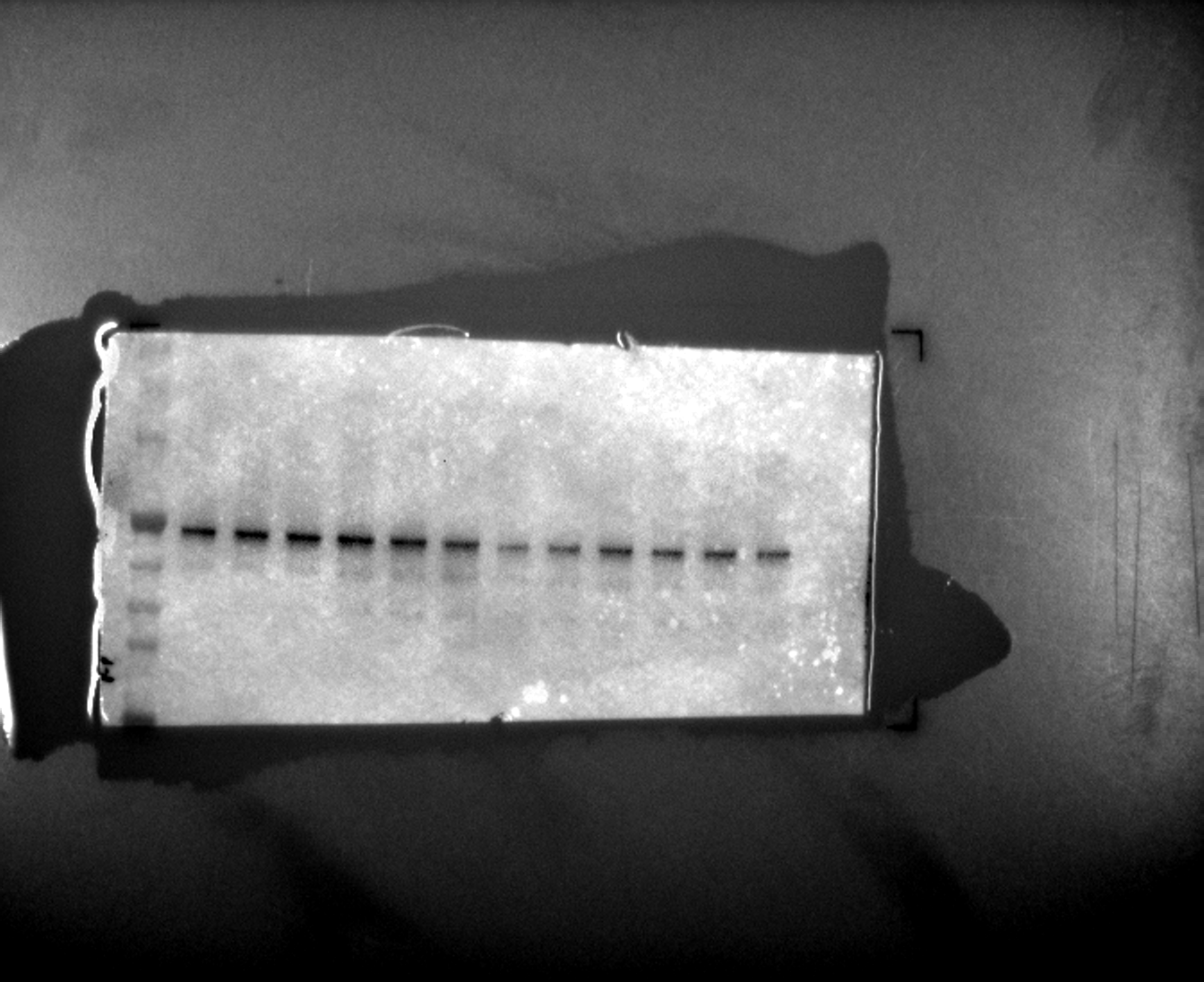

Supplement: Supplementary file 4 [file DataSheet6.ZIP › Figure 6/p-PI3K_Gel-1.Tif]

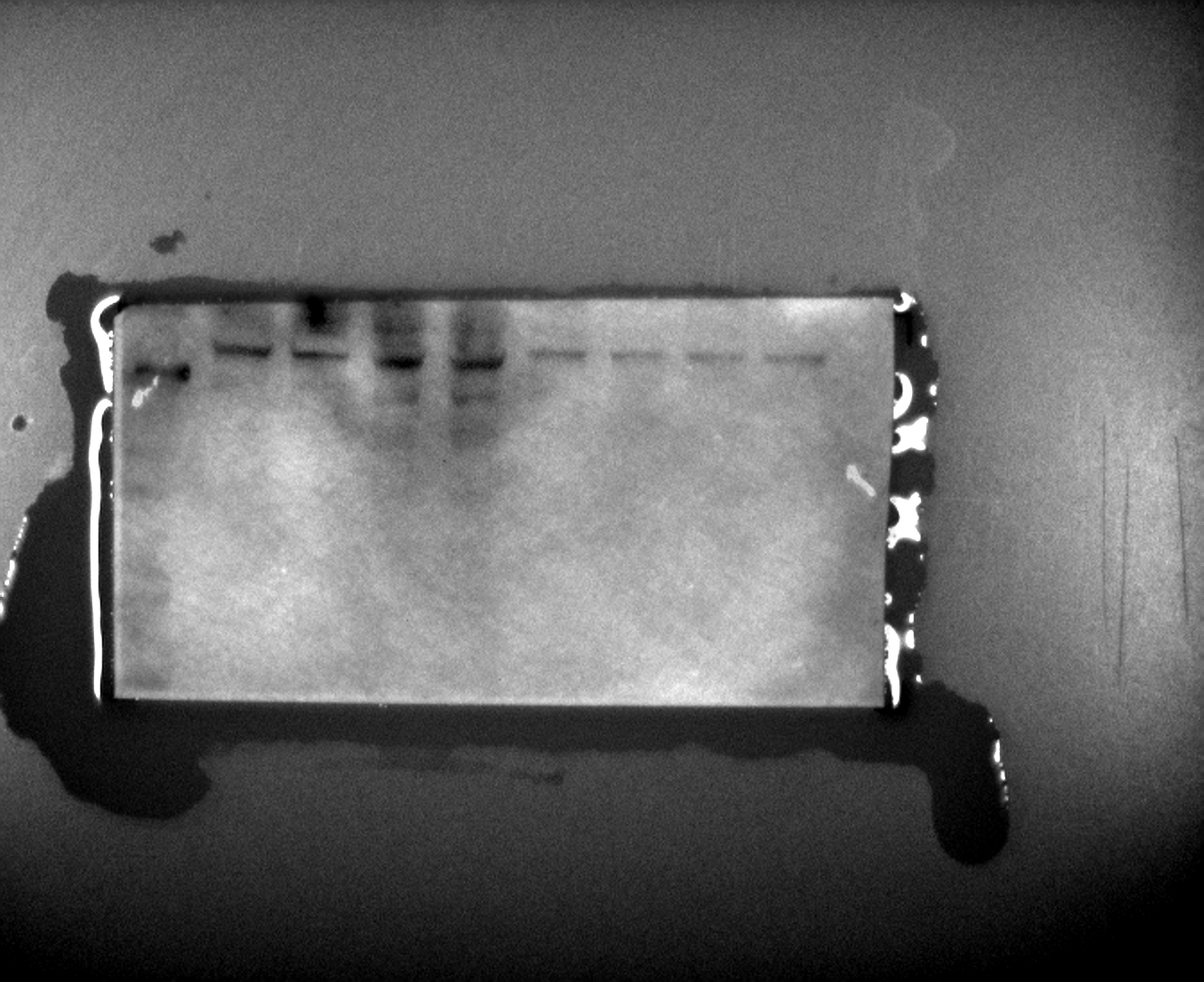

Supplement: Supplementary file 4 [file DataSheet6.ZIP › Figure 6/p-PI3K_Gel-2.Tif]

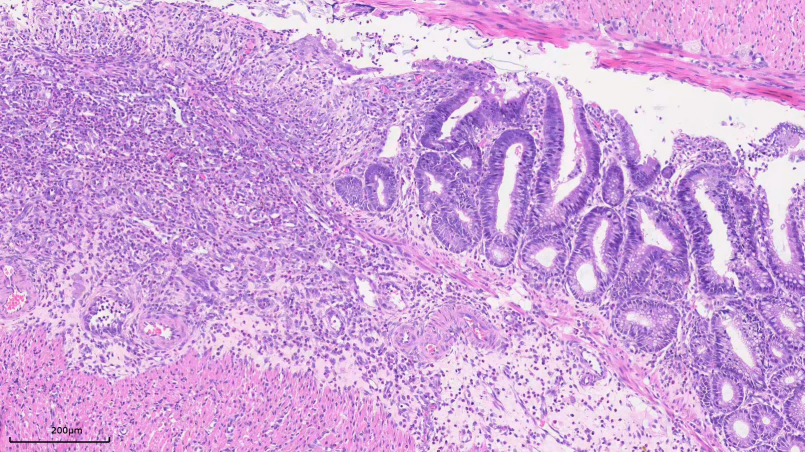

Supplement: Supplementary file 5 [file DataSheet2.ZIP › Figure 2/He Stain/HTP2 200a╠m.png]

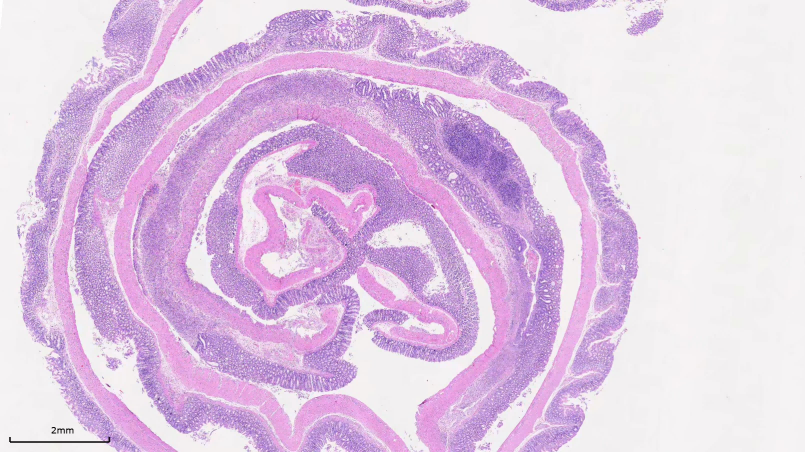

Supplement: Supplementary file 5 [file DataSheet2.ZIP › Figure 2/He Stain/HTP2 2mm.png]

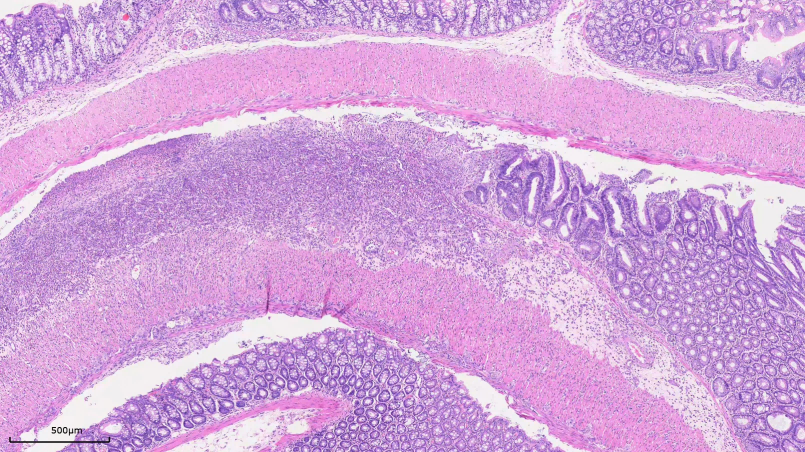

Supplement: Supplementary file 5 [file DataSheet2.ZIP › Figure 2/He Stain/HTP2 500a╠m.png]

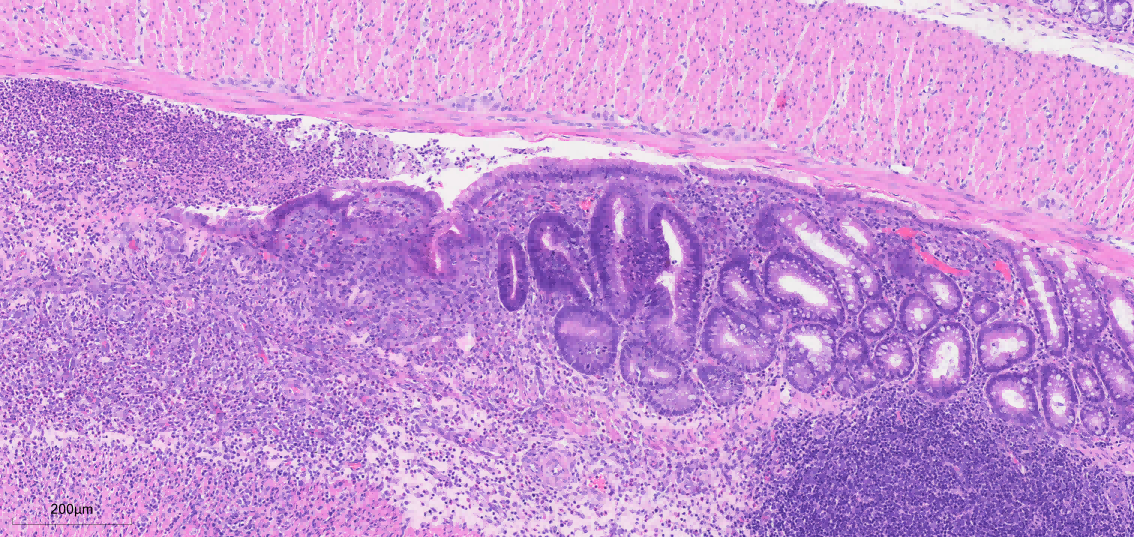

Supplement: Supplementary file 5 [file DataSheet2.ZIP › Figure 2/He Stain/LTP2 200a╠m.png]

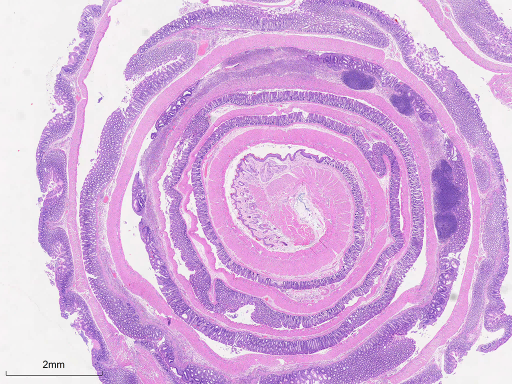

Supplement: Supplementary file 5 [file DataSheet2.ZIP › Figure 2/He Stain/LTP2 2mm.png]

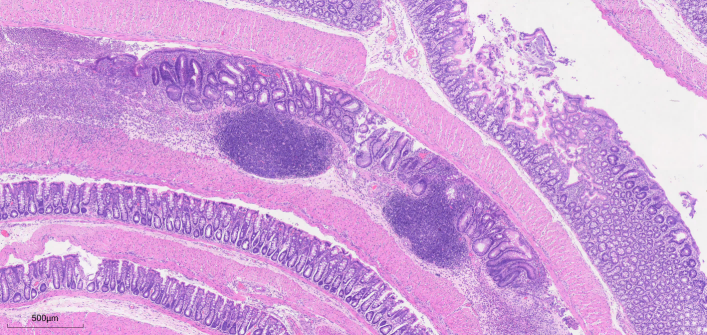

Supplement: Supplementary file 5 [file DataSheet2.ZIP › Figure 2/He Stain/LTP2 500a╠m.png]

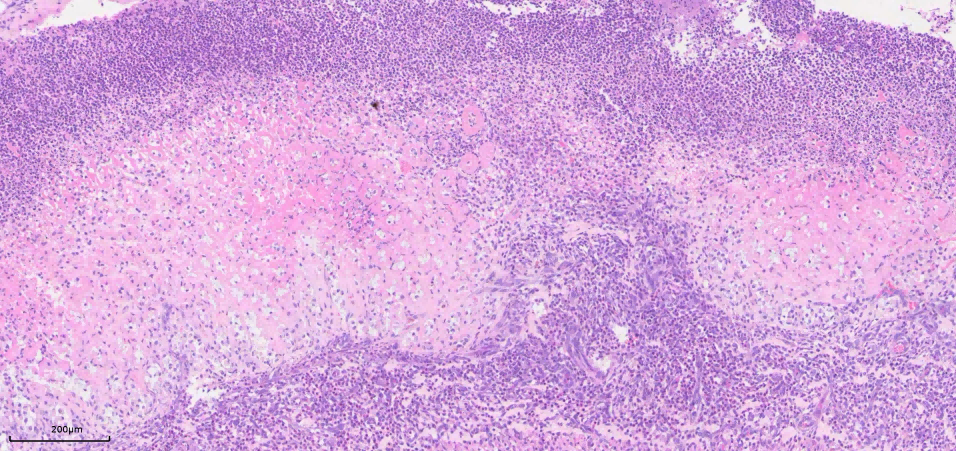

Supplement: Supplementary file 5 [file DataSheet2.ZIP › Figure 2/He Stain/MC 200a╠m.png]

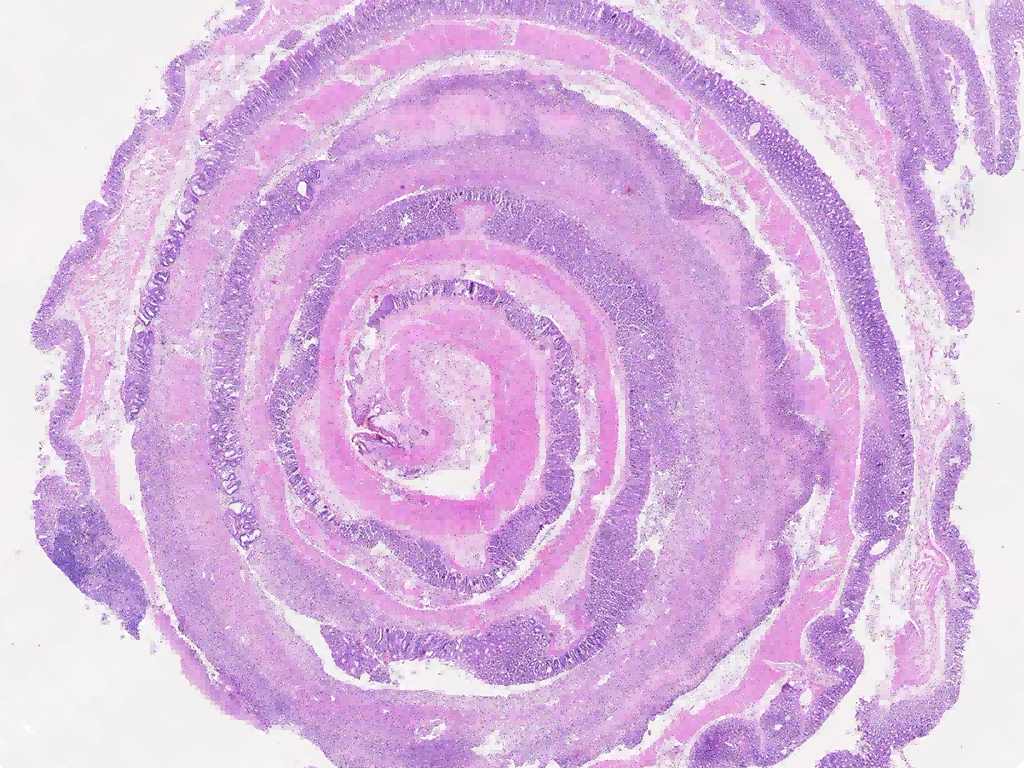

Supplement: Supplementary file 5 [file DataSheet2.ZIP › Figure 2/He Stain/MC 2mm.png]

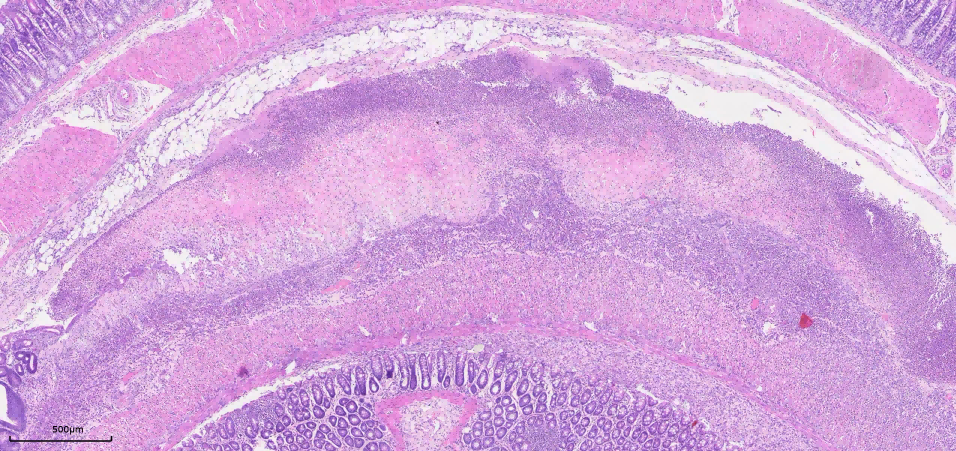

Supplement: Supplementary file 5 [file DataSheet2.ZIP › Figure 2/He Stain/MC 500a╠m.png]

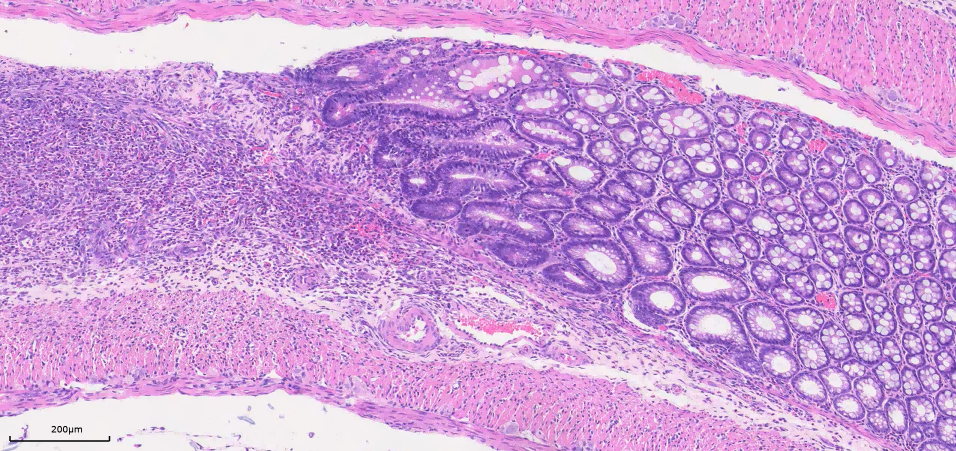

Supplement: Supplementary file 5 [file DataSheet2.ZIP › Figure 2/He Stain/MTP2 200a╠m.png]

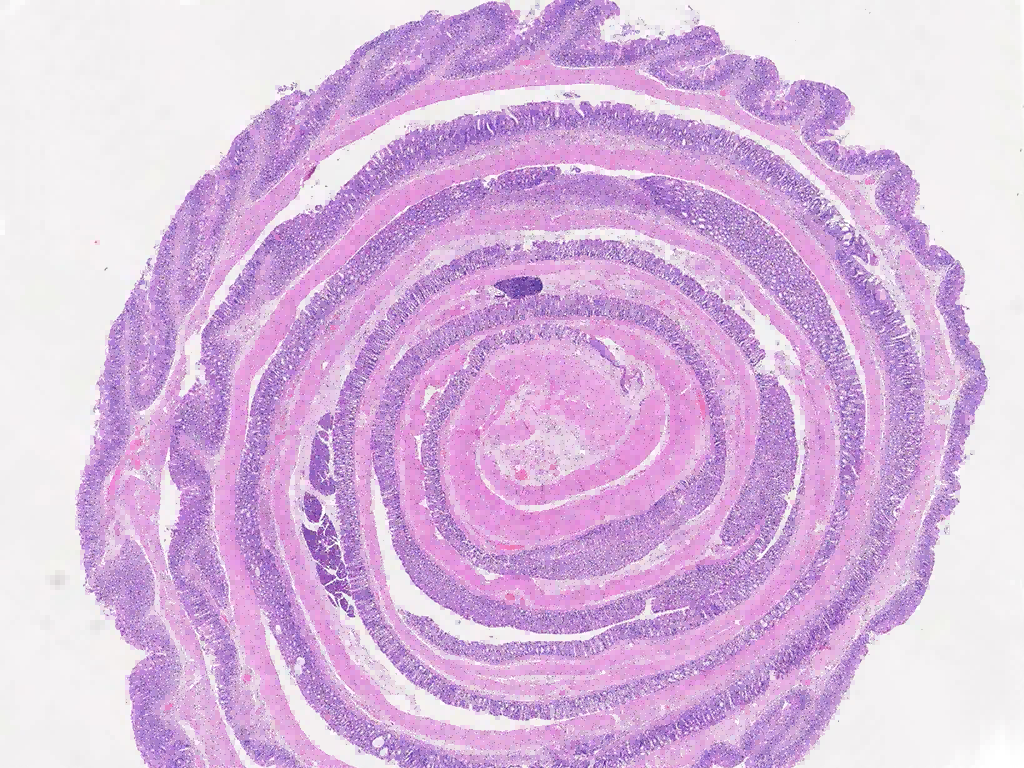

Supplement: Supplementary file 5 [file DataSheet2.ZIP › Figure 2/He Stain/MTP2 2mm.png]

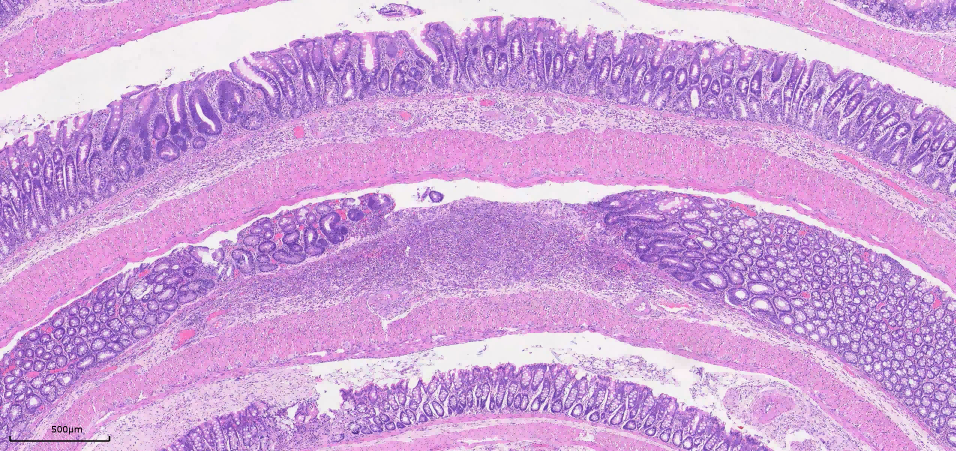

Supplement: Supplementary file 5 [file DataSheet2.ZIP › Figure 2/He Stain/MTP2 500a╠m.png]

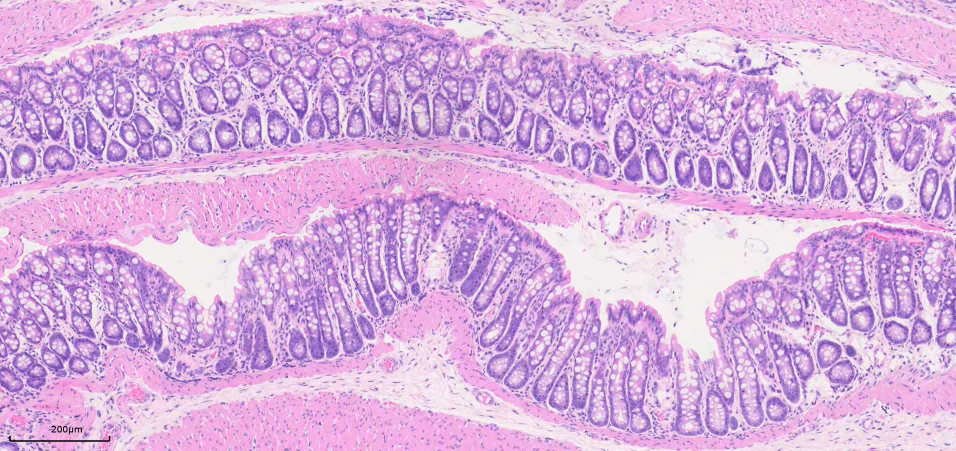

Supplement: Supplementary file 5 [file DataSheet2.ZIP › Figure 2/He Stain/NC 200a╠m.png]

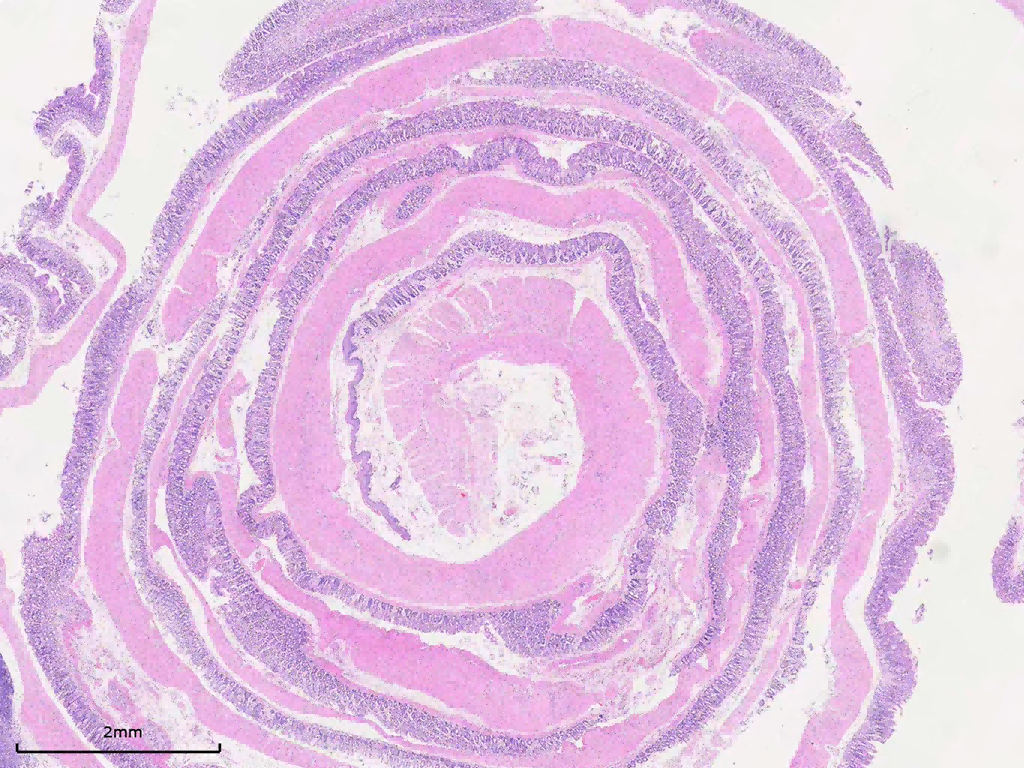

Supplement: Supplementary file 5 [file DataSheet2.ZIP › Figure 2/He Stain/NC 2mm.png]

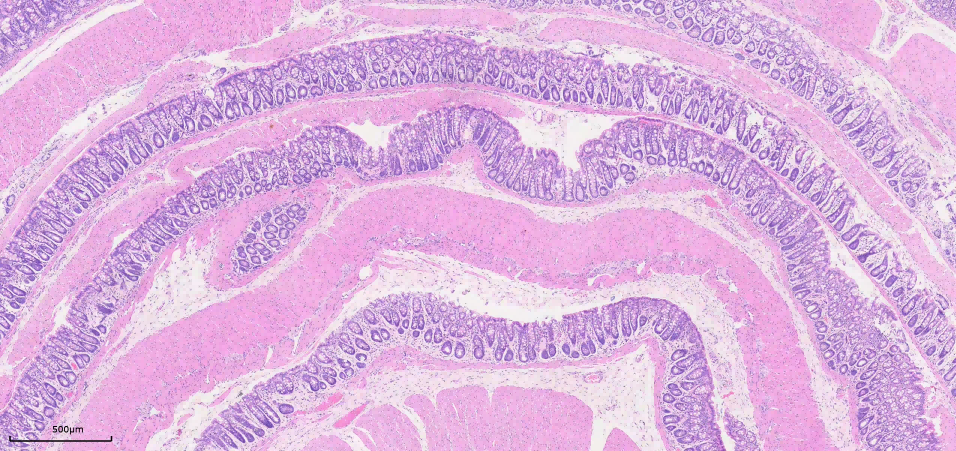

Supplement: Supplementary file 5 [file DataSheet2.ZIP › Figure 2/He Stain/NC 500a╠m.png]

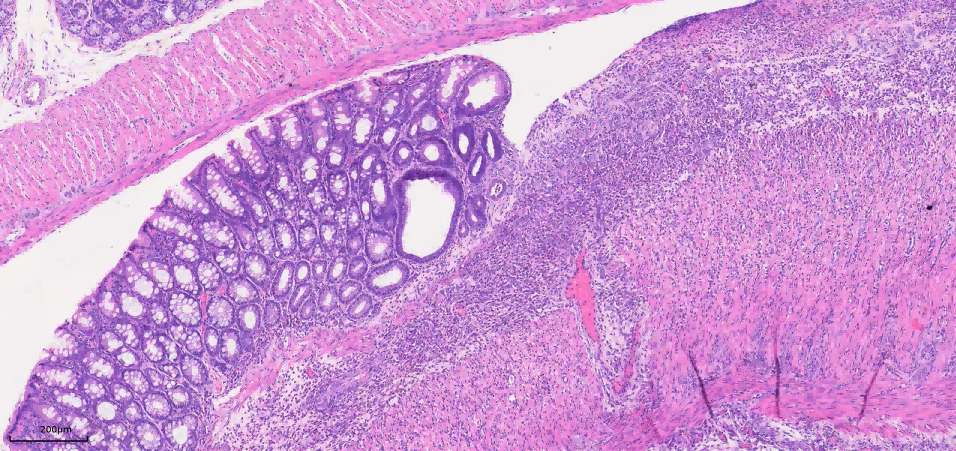

Supplement: Supplementary file 5 [file DataSheet2.ZIP › Figure 2/He Stain/PD 200a╠m.png]

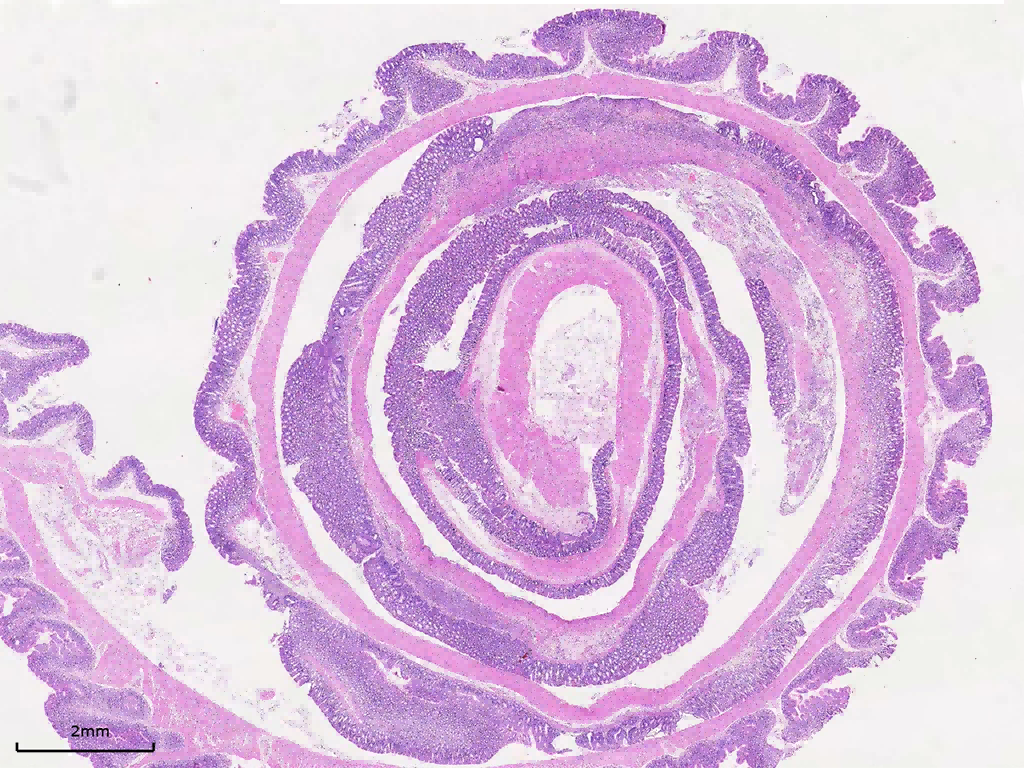

Supplement: Supplementary file 5 [file DataSheet2.ZIP › Figure 2/He Stain/PD 2mm.png]

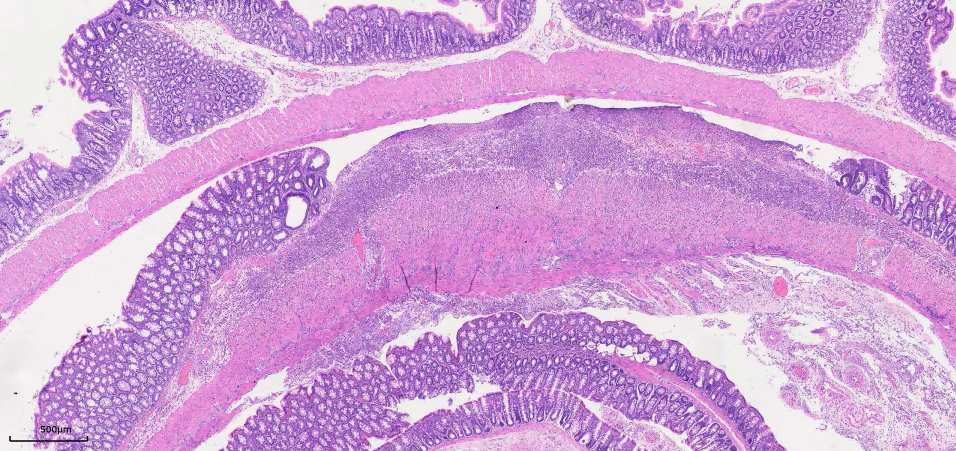

Supplement: Supplementary file 5 [file DataSheet2.ZIP › Figure 2/He Stain/PD 500a╠m.png]
